# Topologically robust programmable processing arrays using light and matter skyrmions

**DOI:** 10.1038/s41467-026-78249-7

**Published:** 2026-10-08

**Authors:** Ruofu Liu, Yunqi Zhang, Yuxi Cai, Yihan Liu, Zhenglin Li, Tianxin Wang, Yifei Ma, Zimo Zhao, Runchen Zhang, Zhi-Kai Pong, Stephen M. Morris, Chao He

**Affiliations:** 1 Department of Engineering Science, University of Oxford, Oxford, UK; 2 Tsinghua University, Haidian District, Beijing, China

**Keywords:** Optical techniques, Applied optics

## Abstract

Photonic computing offers a low-power, high-bandwidth paradigm for information processing; however, the analog nature of conventional architectures means that intrinsic noise and fabrication imperfections greatly impact performance, thereby severely limiting scalability. Recent work on optical skyrmions offers a route to overcoming these limitations by exploiting perturbation-resilient topological invariants of the optical field for computation. Crucially, owing to its relative novelty, an architectural perspective on integrating individual components that manipulate topological charge into a functional system remains an important open challenge. In this paper, we take concrete steps toward system-level design by introducing a platform-independent architecture for skyrmion-based processing, built around a modular library of topologically robust optical primitives, including generators, converters, registers, and adders. This framework enables the synthesis and arithmetic manipulation of topological numbers within a unified programmable architecture. We then experimentally validate this approach using multichannel arrays, demonstrating accurate charge readout and robustness against perturbations in practical optical systems. These results provide a scalable foundation for topologically robust programmable processing arrays, paving the way for compact and integrated photonic processing circuits.

## Abstract (web summary)

Optical skyrmions offer a route to scalable photonic computing via perturbation-resilient topological invariants of the optical field. Here, authors introduce a platform-independent architecture for skyrmion-based processing arrays, built around a library of topologically robust optical primitives.

## Introduction

Topological textures, including optical skyrmions^1–6^ and related structures such as merons^7–9^, are spatially varying fields characterized by a quantized topological charge that is preserved under continuous deformations. Recent work on optical Stokes skyrmions in complex media has directly confirmed this robustness^10–12^, showing that, provided boundary conditions are preserved, the associated topological charge remains highly stable even in the presence of realistic polarization aberrations and noise. This inherent stability implies that topological indices such as the skyrmion number provide a discrete and robust label for a continuous optical field, motivating the use of skyrmions as information carriers for new forms of optical processing^13,14^.

An essential step toward skyrmion-based optical information processing is the generation of optical skyrmions, which has seen rapid advances. In the near field, surface plasmon polariton interference has produced skyrmion lattices that have been imaged with phase-resolved near-field microscopy^15,16^. In free space, tunable skyrmionic beams and related polarization textures have also been engineered^17^ via a variety of different techniques including liquid crystal droplets^18^, spatial light modulators^3,19^ and gradient index systems^20,21^. Additionally, metasurface-assisted surface-emitting lasers point to compact sources^22^, and advances in meta-optics offer rich control of polarization states along the optical path^23–25^. Lastly, q-plates, originally introduced for optical vortex generation, have also recently been extended to skyrmion generation^26,27^.

Despite these advances, from a functional perspective, most studies still focus on generating specific types of optical skyrmions, such as fixed Néel-type^15,28^ or Bloch-type^29^ textures, or focus on complex beams themselves as input and output^19^. A sophisticated framework for matter field design that prescribes how to generate textures of arbitrary type and order remains lacking. Generation alone, moreover, is not enough: to use skyrmions as information carriers one must also manipulate the generated textures and perform programmable operations on their topological charge. Recent works have begun to do this, securing topological resilience in paraxial classical^10^ and non-local quantum^30,31^ skyrmions, exploiting skyrmionic textures for noise-tolerant information transfer and readout^11,12^, generalizing the skyrmion number to higher-dimensional integer tuples to increase encoding capacity^32^, and, most recently, modifying the skyrmion number in a perturbation-resilient way without external energy to realize integer arithmetic^13^. In each, however, the skyrmion mostly serves as a single device-level resource, with each function realized on its own; how such functions can be organized, reused, and scaled has been less explored. In particular, multichannel, parallel processing remains an important open goal for a scalable architecture.

Taken together, these considerations motivate the central question addressed in this paper: how can we devise a broadly applicable framework for the programmable manipulation of polarization textures, encompassing both their generation and a library of topological processing operations, and ultimately extend these concepts to multichannel, parallel architectures? In this work, we aim to address this by developing an architecture-level framework for topological processing. Here, we adopt the notion of an arbitrary retarder^19^—here referring to spatially varying, tunable elliptical retarder based matter fields—to achieve this. Note that retarder fields are inherently device-agnostic, with a designed field capable of being implemented on multiple platforms, including liquid crystal devices and metasurfaces^33–35^ to realize the desired textures, establishing a flexible and broadly applicable framework for skyrmion manipulation. By cascading multiple retarder fields^36^, this framework naturally accommodates both generation and topological processing, with each subsequent field acting on the textures produced upstream to execute required operations.

**Fig. 1:**
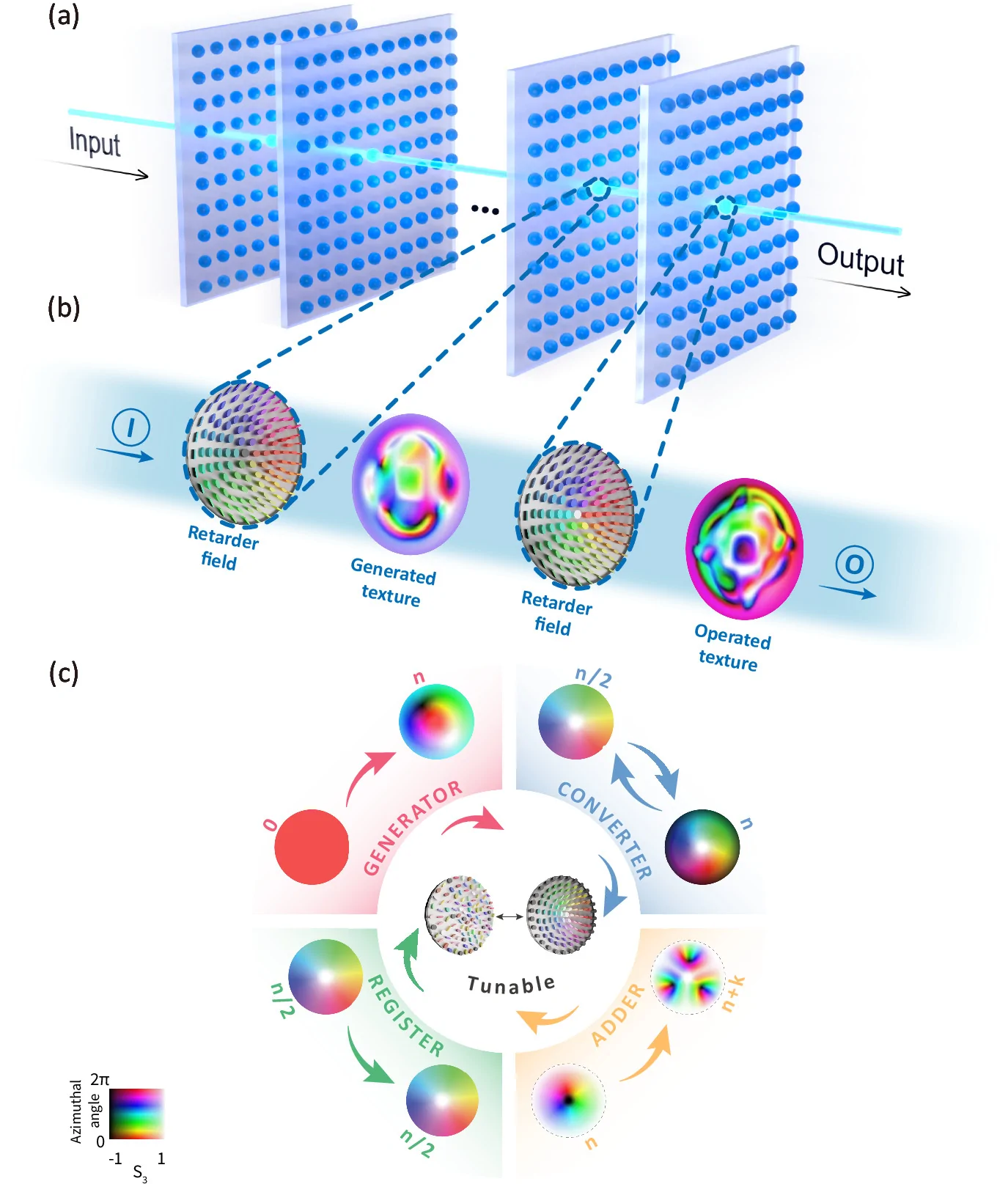
Concept. **a** A multichannel array capable of simultaneously performing a large number of distinct topological processing operations, thereby enabling practical skyrmion-based optical information processing. **b** Each unit of the array consists of a designed, spatially varying retarder selected from a library of distinct units. These units support both generation and processing operations, and can be cascaded to realize complex cascaded operations. Throughout this work, Stokes fields are depicted using color to represent the azimuthal angle on the Poincaré sphere and saturation to represent height, whereas retarder fields are depicted using polarization ellipses to indicate fast-axis orientation. The input field is denoted by **I**, while the output field is denoted by **O**. **c** The four primitives introduced in this work, forming a minimal toolkit for skyrmion-based processing: generators, converters, registers, and adders.

Building on these ideas, we present a platform-independent operational framework for manipulating topological polarization textures based on cascades of retarder fields, as depicted in Fig. 1a, b. The framework adopts merons as the minimal units and consists of four essential optical primitives (Fig. 1c), each responsible for a specific conversion between topological textures: a generator which converts uniform fields (e.g., from light sources) to target polarization textures, a skyrmion-meron interconverter, a meron-to-meron register, and a skyrmion-to-skyrmion adder. Together, these four optical primitives form a minimal operational toolkit for topological processing, whose roles are as follows:

1. The generator synthesizes skyrmions and merons with arbitrary topological charge.
2. The skyrmion-meron interconverter (henceforth the converter) transforms skyrmions into merons and vice versa.
3. The meron-to-meron register (henceforth the register, used here as an architectural abstraction for a topology-preserving meron relay rather than as a temporal storage element in the conventional sense) is devoted exclusively to transporting merons without changing their boundary states and topological charges.
4. The skyrmion-to-skyrmion adder (henceforth the adder) performs arithmetic operations on skyrmions.

With this basic set of primitives, skyrmion fields serve as computational states while meron fields serve as transport states. Apart from practical considerations relating to device complexity (see Results), this distinction between the roles played by skyrmions and merons serves as an important abstraction that simplifies the architecture: a skyrmion is converted into a meron when it is not in use, and converted back into a skyrmion when required. We also note that each primitive exhibits strong topological robustness and the underlying retarder field can be arbitrarily deformed without affecting its operation, provided that the boundary of the primitive remains unchanged (see Results).

To demonstrate the practical feasibility of our framework, we implement programmable retarder fields realized with liquid crystal spatial light modulators (LC-SLMs), and observe accurate charge readout under the aberrations and noise characteristic of practical operation, consistent with our theoretical predictions. Beyond single-channel operation, we also realize multichannel, parallel implementations built from the same library of primitives. The work presented in this paper paves the way for the development of topologically robust programmable processing arrays using optical skyrmions.

## Results

As discussed in the Introduction, the key property that enables topological textures to function as information carriers is the presence of a stable, well-defined topological charge associated with the field. In the case of optical skyrmions, the relevant topological index is the integer-valued skyrmion number (see Supplementary Notes 1 and 2 for detailed definitions), which is derived from the classical notion of the degree of a map and can be computed via the integral

1 $${N}_{{{\rm{sk}}}}=\frac{1}{4{{\rm{\pi }}}}\iint {{\bf{S}}}\cdot \left(\frac{\partial {{\bf{S}}}}{\partial x}\times \frac{\partial {{\bf{S}}}}{\partial y}\right){dxdy}.$$

Note, however, that integer quantization of the above expression requires the field to satisfy specific boundary conditions, often referred to as compactifiability^10^. Without these boundary conditions, the equation above can no longer be identified with the degree of a map, but the field nonetheless exhibits topological character in the sense that the fractional part of ${N}_{{{\rm{sk}}}}$ depends only on the boundary condition of the field^10^.

Exploiting this property, we adopt both skyrmions and merons (for which the field is non-compactifiable and ${N}_{{{\rm{sk}}}}$ lies in ${\mathbb{Z}}/2$) as basic units for information processing. Such an approach offers two key advantages. First, from a design perspective, having two distinct units allows them to be assigned different roles, which in our case correspond to information processing (skyrmions) and information transport (merons). Second, increasing ${N}_{{{\rm{sk}}}}$, whether integer or fractional, leads to increasing complexity of the underlying polarization field. This, in turn, results in practical engineering limits due to limited resolution of the underlying matter field arising from finite pixel size. Adopting merons and skyrmions together thus helps overcome these real-world issues. For any given maximum topological charge, three times as many distinct units become available. The same operation can then be realized with only a third of the maximum charge, which reduces the overall system complexity. Naturally, other fractional units could be employed to further increase the number of computational units, and we leave a detailed investigation of their design as a possible extension of this work.

The key approach for designing primitives that convert between different units relies on precise manipulation of the field boundary (see Supplementary Note 3). As a result, the functionality of each unit depends only on how the incident field’s boundary curve deforms as the field propagates through the device. This gives rise to strong topological robustness, similar to that established in ref.^13^, whereby perturbations to the underlying retarder field that do not act on the boundary do not affect device functionality. In addition to relaxing fabrication and engineering constraints, this property is well suited to cascaded architectures, since the operation of each primitive depends only on the boundary curve of the field exiting the preceding primitive.

Adopting the approach outlined above, we begin by introducing the first of our four primitives, namely generators, which convert uniform incident light into skyrmions and merons of arbitrary order. The underlying retarder field features a fast-axis distribution corresponding to an order-*n* meron, together with a radial retardance profile $\delta \left(r\right)={{\rm{\pi }}}r$ for skyrmion generation and $\delta \left(r\right)={{\rm{\pi }}}r/2$ for meron generation (see Supplementary Note 3). These generate skyrmions and merons of order *n*.

**Fig. 2:**
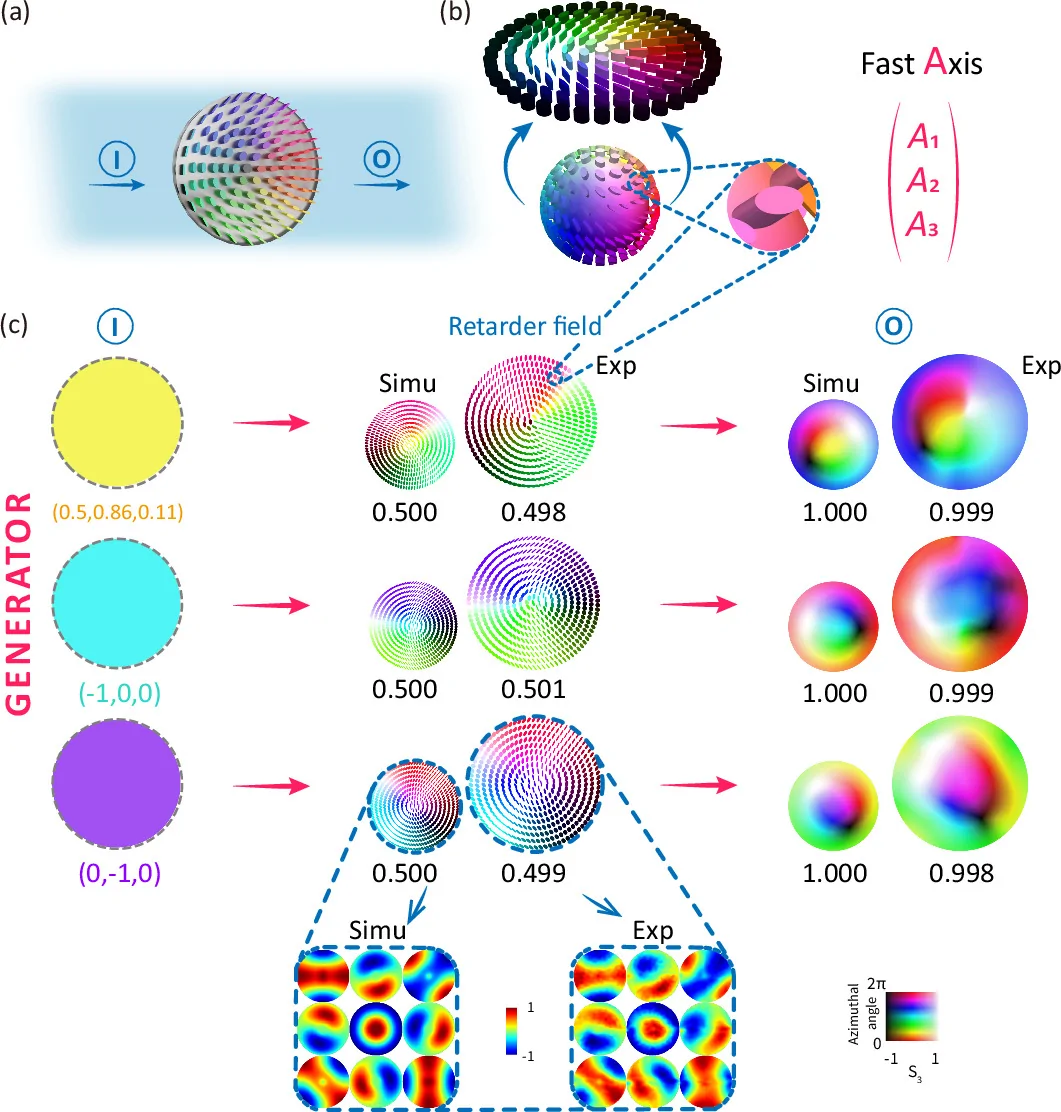
Skyrmion generator. **a** Input field passes through an arbitrary retarder field, and the resulting output field forms Stokes skyrmions. **b** A magnified view of a single element within the fast-axis distribution is shown, which depicts the vector ${{\bf{A}}}={({A}_{1},{A}_{2},{A}_{3})}^{{{\rm{\top }}}}$ (see Supplementary Note 5). The color of the ellipse represents the azimuthal orientation of the fast axis, while its ellipticity represents the height. **c** Theoretical and experimentally measured Stokes fields generated by various cases. The input fields are uniform, while the output fields form skyrmions with a topological charge of 1. The retarder field of each case with a topological charge of 0.5 is also shown, depicted through the fast-axis distribution. Lastly, the simulated and experimentally measured Mueller matrix corresponding to the case of −45° polarized incident field is given.

To demonstrate the feasibility of the framework, we realize the retarder field using a cascade of three liquid crystal spatial light modulators (LC-SLMs), as shown in Fig. 2a (see Supplementary Note 4 for experimental details and phase decomposition). In parallel, we characterize the retarder field via Mueller matrix polarimetry^37–43^ and extract its fast-axis orientation by Lu–Chipman decomposition^44^ (see Supplementary Note 5).

Figure 2c presents experimentally generated Stokes fields^45,46^ for various skyrmion generators designed using the framework above, together with the measured fast-axis distributions of the corresponding primitives. Simulated results are also shown for comparison. Notice from the figure that since our primitives are designed to perform a specific operation on boundary curves, the resultant Stokes fields are not confined to any single type (see Supplementary Notes 6 and 7). The numerically computed topological charge, evaluated using Eq. (1), is also shown, confirming that the primitives generate skyrmions with the correct topological charge. We emphasize that this charge is mainly determined by the boundary of the field rather than by the pointwise interior texture, so that local deviations in the interior texture between measurement and simulation rarely affect the measured skyrmion number, consistent with the topological protection established in Supplementary Notes 3 and 8. Lastly, Supplementary Note 7 presents additional experimental results, with examples of meron generation.

Next, we elevate the platform to topological processing. To achieve this, we introduce a second retarder field downstream. In this stage, the generated Stokes fields are manipulated via three primitives: the converter, the register, and the adder (Fig. 1c). An important feature of our design is the reusability of different matter fields. More specifically, in converters, the underlying retarder field is the same as that used for the meron generator, while in adders and registers it is the same as that used for the skyrmion generator (in the case of registers, the underlying retarder field exhibits a boundary fast-axis distribution aligned with the incident field and may adopt an arbitrary retardance profile; $\delta (r)={{\rm{\pi }}}r$ is used in this work for consistency). This demonstrates the multifunctionality of our approach, which is crucial for performing different computations using the same architecture, and underscores the modular nature of the designed optical primitives. In each case, the underlying matter field is similarly a meron-like field, with a detailed description presented in Supplementary Note 3.

**Fig. 3:**
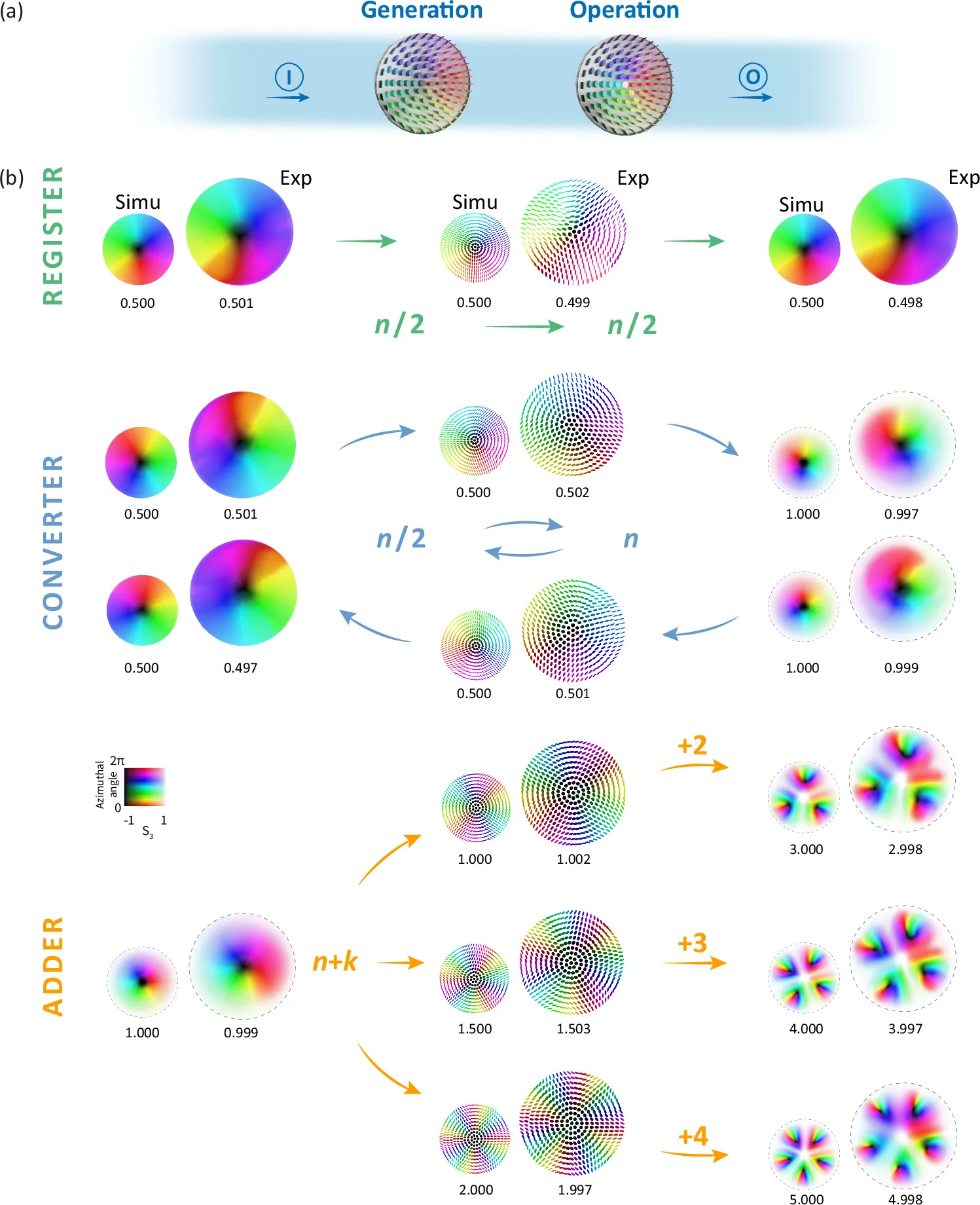
Primitives for topological processing. **a** Schematic illustrating the cascaded architecture used to perform processing operations sequentially. **b** Simulated and experimentally measured Stokes fields of the input and output fields for each primitive. The corresponding fast-axis distribution for each primitive is also shown. (Top) A register that converts an order-1 meron into another order-1 meron. (Middle) A converter which switches between an order-1 meron and an order-1 skyrmion. (Bottom) Adders of orders 2, 3, and 4 acting on a skyrmion of order-1.

With the primitives defined, Fig. 3b presents experimentally measured Stokes fields and corresponding simulations for representative examples. Specifically, results are shown for the converter, register, and adders of orders 2, 3, and 4. In each case, the fast-axis distributions extracted from the Mueller matrices are also presented. The agreement is excellent, as the measured Stokes fields closely match the simulations and the charge readout reproduces the design within experimental uncertainty. Importantly, note that while only a single operation is demonstrated here, the primitives introduced can be further cascaded to achieve complex composed operations (see Supplementary Note 3).

**Fig. 4:**
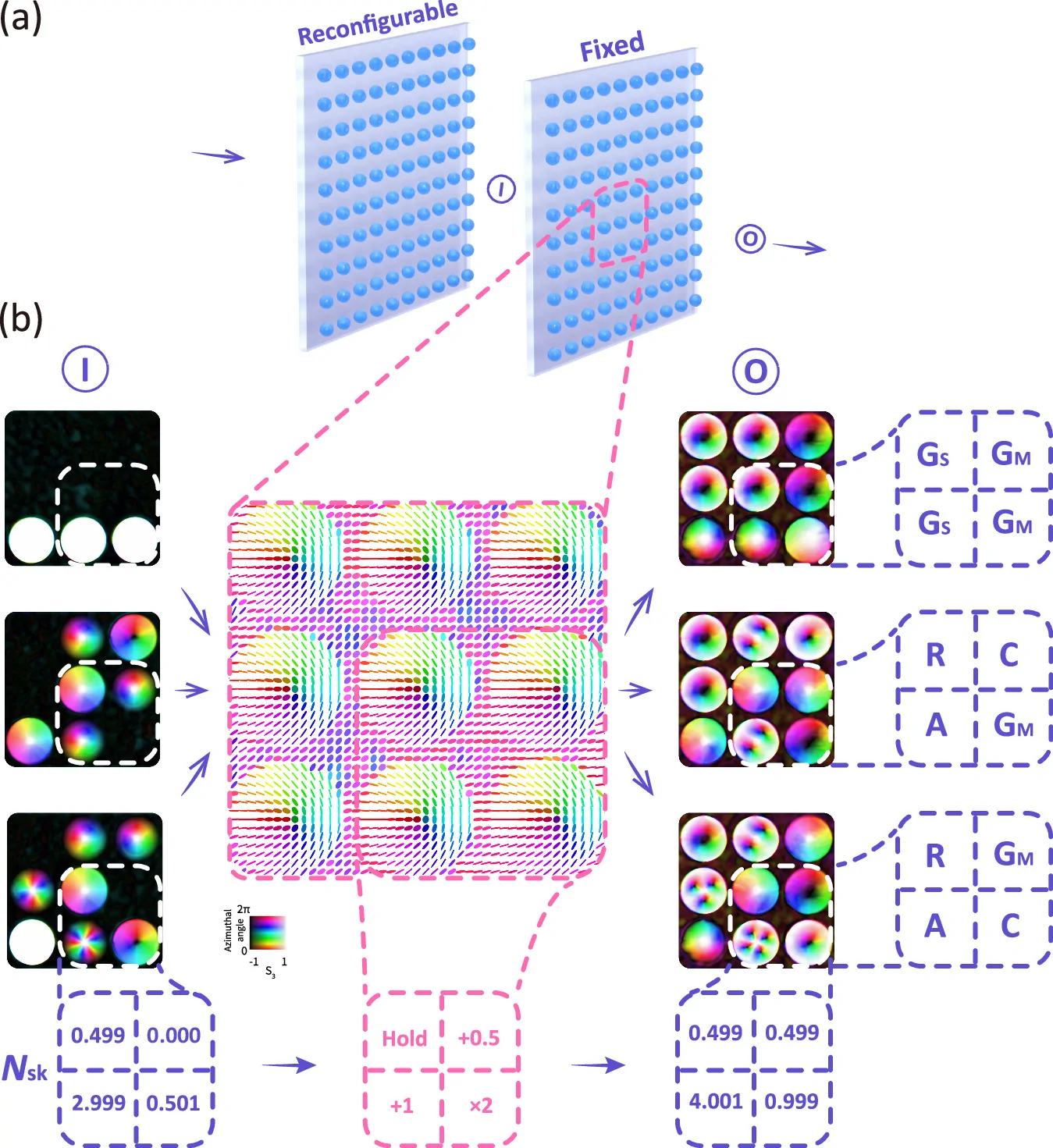
Multichannel array and functional reusability. **a** A reconfigurable stage for state preparation followed by a fixed processing stage to demonstrate architectural reusability. **b** A multichannel array is used to demonstrate the simultaneous performance of distinct processing operations and the reusability of the architecture. Three different sets of 3 × 3 incident arrays passed through the same 3 × 3 retarder field array to produce various output configurations, illustrating that a single reconfigurable platform can be repurposed for multiple functions by modulating the input state. The specific operations performed by the bottom-right 2 × 2 area are annotated: G_S_ (skyrmion generator), G_M_ (meron generator), R (register), C (converter), and A (adder). For the third (bottom) set of results, the experimentally measured skyrmion numbers ${N}_{{{\rm{sk}}}}$ are provided alongside their corresponding arithmetic operations.

Here, to demonstrate parallel operation and architectural reusability, we implement a multichannel array utilizing a dual-stage configuration (Fig. 4a). More specifically, we employ a reconfigurable layer for state preparation followed by a fixed processing layer for operation. Here, we focus on a representative 3 × 3 array in which the various operations which change according to input can be clearly observed (see Supplementary Note 9 for a higher-order 10 × 10 implementation).

As shown in Fig. 4b, within the annotated bottom-right 2 × 2 area of the array, different functionalities including skyrmion and meron generation (G_S_ and G_M_), as well as information relay (R), conversion (C), and addition (A) are achieved when different input fields are changed. Moreover, accurate skyrmion numbers derived from the experimental Stokes fields (bottom panel) not only validate the execution of these operations but also serve as quantitative confirmation of independent channel configuration, with the per-channel deviation $|{N}_{{{\rm{sk}}},\exp }-{N}_{{{\rm{sk}}},{{\rm{simu}}}}|$ not exceeding 0.003 across all 9 channels (full statistics in Supplementary Note 10 and Supplementary Data). Crucially, these results show that a single reconfigurable stage (in comparison to the two reconfigurable stages used in previous demonstrations) can be used to perform varied and complex operations, offering significant real-world advantages, as reconfigurable components are often larger, more costly, and difficult to fabricate.

## Discussion

In this work, we present and experimentally validate a platform-independent operational framework for manipulating the topological charge of polarization textures, enabling skyrmion-based optical processing encompassing generation, conversion, transport, and arithmetic. Our approach uses a library of pre-designed topologically robust optical primitives, which modularizes the design of topological processing and enables systematic composition, reuse, and scaling of complex operations. An important aspect of our proposed approach is that distinct operations can be realized with the same underlying primitive. This enables a single reconfigurable generation layer to act as a way of selecting between different operations implemented with a fixed processing layer, thereby achieving operation-level reconfigurability using only a single dynamic stage. Crucially, this layered structure extends naturally to the array level, as demonstrated by the 3 × 3 and 10 × 10 parallel implementations reported here (see Supplementary Note 9 for the 10 × 10 array). As such, we anticipate that the framework presented in this paper will provide a practical foundation for scalable, reconfigurable skyrmion-based optical information processing.

This framework builds on a natural division of labor between skyrmions and merons. Beyond the information-density argument introduced in the Results, the skyrmion–meron role separation provides further advantages. The main advantage is reuse through unification: the generator, converter, and adder share a common pattern, a meron fast-axis distribution derived in Supplementary Note 3, differing only in the scalar retardance *δ*. A meron register naturally keeps this shared pattern, whereas a skyrmion register would require a skyrmion fast-axis distribution that breaks it. Thus, using a meron for transport keeps all four primitives in one matter-design family and enables field reuse. Furthermore, because the primitives differ only in *δ*, a tunable-retardance device^34^ could in principle collapse the whole library onto a single element. We also note that a further advantage arises in confined-mode platforms such as waveguides, where the natural propagating state is often a meron rather than a skyrmion^7^. Treating merons as equal carriers to skyrmions thus matches what these platforms support.

From an engineering perspective, the topological nature of our approach implies robustness against the disorder in practical optical systems (see Supplementary Note 8), a key property that is not only highly relevant for optical communications and interconnections^7^, but also not present in existing optical computing strategies^47–49^. Indeed, our experiments confirm that the operations remain reliable despite a certain level of practical imperfections inevitably present in our setup, including misalignment between cascaded devices, retardance and angular errors, the temperature dependence of liquid crystal devices, and ambient mechanical vibrations, which fall within the regime where topological resilience is maintained. Beyond reliable operation, this robustness also lowers the fabrication demands of each platform, such as liquid crystal polymers^50,51^, femtosecond-laser-written birefringent glasses^52,53^, and dielectric metasurfaces^54^, making the framework easier to implement across different technologies (see Supplementary Note 11). Such platform flexibility means the approach can move toward more compact, integrated photonic processing circuits.

Before concluding, several important extensions of this work are worth discussing. First, while the present work operates in free space and keeps each layer in an imaging configuration via 4f relays, moving toward more integrated implementations requires relaying the field between elements through free-space propagation or confined media such as waveguides^7^, where preserving the topological charge becomes the key question. Addressing this needs engineering the inter-element relay and/or developing advanced topological frameworks^32^ that remain robust under propagation, making the preservation of topological charge an important extension of this work. Second, while we consider only the conventional skyrmion number in this work, it is possible to extend these operations to the generalized skyrmion number^32^, which has the potential to further enhance data density. Third, while topological protection provides robustness against perturbations within each operation, the quality of the boundary polarization states remains an important challenge. This motivates the development of cutting-edge vectorial adaptive optics strategies that focus on compensating such errors^55,56^. Fourth, a promising direction is to align this optical framework with the full digital logic abstraction of conventional electronics, such as Boolean operations and fan-in/fan-out routing. One route to multi-input fan-in and multi-output fan-out is to introduce a diffractive element into the optical path to couple otherwise independent channels^12,57^; bridging toward Boolean logic^58–60^ is also a further step along this direction. Together, these point to a wide space for future topological processing architectures.

To conclude, despite growing interest in photonic computing as a low-power, high-bandwidth approach for specialized information processing^48,49,61–63^, robustness to noise remains a central challenge for scalability^47–49^. Optical skyrmions address this challenge by providing topologically protected information carriers that are intrinsically resilient to noise; however, the novelty of this approach means that high-level architectural design remains an open challenge. In this paper, we introduce a set of primitives that form the building blocks of a higher-level architectural framework for skyrmion-based optical information processing, providing a concrete starting point for system-level design. As such, we believe these primitives offer a practical route toward scalable photonic architectures that exploit topological robustness for reliable information processing.

## Supplementary information

Supplementary Information

Transparent Peer Review file

## Data Availability

The data supporting the findings of this study are available within the article and its Supplementary Information files. The complete per-channel skyrmion number measurements for the 3 × 3 and 10 × 10 arrays and the full numerical datasets for the three noise-perturbation experiments are provided as Supplementary Data. Additional data are available from the corresponding author upon reasonable request.

## References

[1] Shen, Y. et al. Optical skyrmions and other topological quasiparticles of light. *Nat. Photonics* **18**, 15–25 (2024).

[2] Zhang, Y. et al. Skyrmions based on optical anisotropy for topological encoding. *Light Sci. Appl.* **15**, 254 (2026). 10.1038/s41377-026-02307-4 PMC13213056 42192097

[3] Wu, H., Zhou, W., Zhu, Z. & Shen, Y. Optical skyrmion lattices accelerating in a free-space mode. *APL Photonics* **10**, 050804 (2025).

[4] Ma, Y. et al. Using optical skyrmions to assess vectorial adaptive optics capabilities in the presence of complex aberrations. *Sci. Adv.* **11**, eadv7904 (2025). 10.1126/sciadv.adv7904 PMC12494033 41042885

[5] Kam, A. et al. Quantum skyrmions and high dimensional entanglement mediated by nanophotonics. *eLight* **6**, 13 (2026).

[6] Liu, Y. et al. Broadband coloured skyrmions generated by on-chip ferroelectric spherulites. *eLight* **6**, 18 (2026).

[7] Wang, A. A. et al. Optical Skyrmions in Waveguides. Preprint at https://arxiv.org/abs/2505.06735 (2025).

[8] Yessenov, M. et al. Ultrafast space-time optical merons in momentum-energy space. *Nat. Commun.* **16**, 8592 (2025). 10.1038/s41467-025-63641-6 PMC12480155 41022730

[9] Król, M. et al. Observation of second-order meron polarization textures in optical microcavities. *Optica* **8**, 255 (2021).

[10] Wang, A. A. et al. Topological protection of optical skyrmions through complex media. *Light Sci. Appl.* **13**, 314 (2024). 10.1038/s41377-024-01659-z PMC11582597 39572554

[11] Peters, C., Everts, K., Kleine, T., Ornelas, P. & Forbes, A. Seeing through randomness with topological light. Preprint at https://arxiv.org/abs/2508.12305 (2025).

[12] Bezuidenhout, H., Peters, C., Kumar, R., Forbes, A. & Nape, I. Deep diffractive optical neural networks for detecting Skyrmionic topologies of light. Preprint at https://arxiv.org/abs/2512.11115 (2025). 10.1038/s41467-026-77376-5 PMC13646250 42844263

[13] Wang, A. A. et al. Perturbation-resilient integer arithmetic using optical skyrmions. *Nat. Photonics* **19**, 1367–1375 (2025).

[14] He, C., Shen, Y. & Forbes, A. Towards higher-dimensional structured light. *Light Sci. Appl.* **11**, 205 (2022). 10.1038/s41377-022-00897-3 PMC9256673 35790711

[15] Tsesses, S. et al. Optical skyrmion lattice in evanescent electromagnetic fields. *Science* **361**, 993–996 (2018). 10.1126/science.aau0227 30026318

[16] Zhou, Y. et al. Generating one-dimensional plasmonic arrays by laser-driven self-organization. *Light Adv. Manuf.* **6**, 57 (2025).

[17] Zhu, J., Liu, S., Zhang, Y.-S., Li, C.-F. & Guo, G.-C. Synthesis and observation of optical skyrmionic structure in free space. *Phys. Rev. A* **110**, 043522 (2024).

[18] Kamal, W. et al. On-demand pitch tuning of printed chiral nematic liquid crystal droplets. *Mater. Today Adv.* **19**, 100416 (2023).

[19] He, C. et al. A reconfigurable arbitrary retarder array as complex structured matter. *Nat. Commun.* **16**, 4902 (2025). 10.1038/s41467-025-59846-4 PMC12117100 40425556

[20] He, C. et al. Complex vectorial optics through gradient index lens cascades. *Nat. Commun.* **10**, 4264 (2019). 10.1038/s41467-019-12286-3 PMC6753074 31537802

[21] Shen, Y. et al. Topologically controlled multiskyrmions in photonic gradient-index lenses. *Phys. Rev. Appl.* **21**, 024025 (2024).

[22] Shitrit, N. Surface-emitting lasers meet metasurfaces. *Light Sci. Appl.* **13**, 37 (2024). 10.1038/s41377-023-01369-y PMC10831054 38296950

[23] Dorrah, A. H., Rubin, N. A., Zaidi, A., Tamagnone, M. & Capasso, F. Metasurface optics for on-demand polarization transformations along the optical path. *Nat. Photonics* **15**, 287–296 (2021).

[24] Li, X., Lan, T.-H., Tien, C.-H. & Gu, M. Three-dimensional orientation-unlimited polarization encryption by a single optically configured vectorial beam. *Nat. Commun.* **3**, 998 (2012). 10.1038/ncomms2006 PMC4354277 22893122

[25] Gu, T., Kim, H. J., Rivero-Baleine, C. & Hu, J. Reconfigurable metasurfaces towards commercial success. *Nat. Photonics* **17**, 48–58 (2023).

[26] Hakobyan, V., Shen, Y. & Brasselet, E. Unitary spin-orbit optical-skyrmionic wave plates. *Phys. Rev. Appl.* **22**, 054038 (2024).

[27] Hakobyan, V. & Brasselet, E. Q-Plates: from optical vortices to optical skyrmions. *Phys. Rev. Lett.* **134**, 083802 (2025). 10.1103/PhysRevLett.134.083802 40085861

[28] Lei, X. et al. Photonic spin lattices: symmetry constraints for skyrmion and meron topologies. *Phys. Rev. Lett.* **127**, 237403 (2021). 10.1103/PhysRevLett.127.237403 34936800

[29] Gao, S. et al. Paraxial skyrmionic beams. *Phys. Rev. A* **102**, 053513 (2020).

[30] Ornelas, P., Nape, I., de Mello Koch, R. & Forbes, A. Non-local skyrmions as topologically resilient quantum entangled states of light. *Nat. Photonics* **18**, 258–266 (2024).

[31] Ornelas, P., Nape, I., de Mello Koch, R. & Forbes, A. Topological rejection of noise by quantum skyrmions. *Nat. Commun.* **16**, 2934 (2025). 10.1038/s41467-025-58232-4 PMC11937260 40133306

[32] Wang, A. A. et al. Redefining topological robustness in optical polarization fields through a generalized skyrmion number. Nat. Commun. **17**, 8539 (2026). 10.1038/s41467-026-75120-7 PMC13483919 42431895

[33] Rubin, N. A., Shi, Z. & Capasso, F. Polarization in diffractive optics and metasurfaces. *Adv. Opt. Photonics* **13**, 836 (2021).

[34] Shaltout, A. M., Shalaev, V. M. & Brongersma, M. L. Spatiotemporal light control with active metasurfaces. *Science* **364**, eaat3100 (2019). 10.1126/science.aat3100 31097638

[35] Dorrah, A. H. & Capasso, F. Tunable structured light with flat optics. *Science* **376**, eabi6860 (2022). 10.1126/science.abi6860 35446661

[36] Wang, A. A., Cai, Y., Ma, Y., Salter, P. S. & He, C. Resolving topological obstructions to vectorial structured field control. Preprint at https://arxiv.org/abs/2601.11410 (2026).

[37] Azzam, R. M. A. Photopolarimetric measurement of the Mueller matrix by Fourier analysis of a single detected signal. *Opt. Lett.* **2**, 148 (1978). 10.1364/ol.2.000148 19684668

[38] Goldstein, D. H. Mueller matrix dual-rotating retarder polarimeter. *Appl. Opt.* **31**, 6676 (1992). 10.1364/AO.31.006676 20733896

[39] Azzam, R. M. A. Stokes-vector and Mueller-matrix polarimetry. *J. Opt. Soc. Am. A* **33**, 1396 (2016). 10.1364/JOSAA.33.001396 27409699

[40] He, C. et al. Full Poincaré polarimetry enabled through physical inference. *Optica* **9**, 1109 (2022).

[41] Zhang, Z. et al. Analysis and optimization of aberration induced by oblique incidence for in vivo tissue polarimetry. *Opt. Lett.* **48**, 6136 (2023). 10.1364/OL.501365 38039210

[42] Deng, L. et al. Influence of hematoxylin and eosin staining on linear birefringence measurement of fibrous tissue structures in polarization microscopy. *J. Biomed. Opt.* **28**, 102909 (2023). 10.1117/1.JBO.28.10.102909 PMC10541683 37786544

[43] Deng, L. et al. A dual-modality imaging method based on polarimetry and second harmonic generation for characterization and evaluation of skin tissue structures. *Int. J. Mol. Sci.* **24**, 4206 (2023). 10.3390/ijms24044206 PMC9966533 36835613

[44] Lu, S.-Y. & Chipman, R. A. Interpretation of Mueller matrices based on polar decomposition. *J. Opt. Soc. Am. A* **13**, 1106 (1996).

[45] Cai, Y., Wang, A. A. & He, C. Rethinking conditioning in polarimetry: a new framework beyond ℓ2-based metrics. Preprint at https://arxiv.org/abs/2512.23689 (2025).

[46] He, C. et al. Polarisation optics for biomedical and clinical applications: a review. *Light Sci. Appl.* **10**, 194 (2021). 10.1038/s41377-021-00639-x PMC8458371 34552045

[47] Zhou, W. et al. In-memory photonic dot-product engine with electrically programmable weight banks. *Nat. Commun.* **14**, 2887 (2023). 10.1038/s41467-023-38473-x PMC10199927 37210411

[48] Feldmann, J. et al. Parallel convolutional processing using an integrated photonic tensor core. *Nature* **589**, 52–58 (2021). 10.1038/s41586-020-03070-1 33408373

[49] Shen, Y. et al. Deep learning with coherent nanophotonic circuits. *Nat. Photonics* **11**, 441–446 (2017).

[50] Jiang, M. et al. Low *f*-number diffraction-limited Pancharatnam–Berry microlenses enabled by plasmonic photopatterning of liquid crystal polymers. *Adv. Mater.* **31**, 1808028 (2019). 10.1002/adma.201808028 30907480

[51] Tartan, C. C. et al. Generation of 3-dimensional polymer structures in liquid crystalline devices using direct laser writing. *RSC Adv.* **7**, 507–511 (2017).

[52] Beresna, M., Gecevičius, M., Kazansky, P. G. & Gertus, T. Radially polarized optical vortex converter created by femtosecond laser nanostructuring of glass. *Appl. Phys. Lett.* **98**, 201101 (2011).

[53] Allison, J. et al. Laser writing in glass for dense, fast and efficient archival data storage. *Nature* **650**, 606–612 (2026). 10.1038/s41586-025-10042-w PMC12916313 41709004

[54] Chen, H.-T., Taylor, A. J. & Yu, N. A review of metasurfaces: physics and applications. *Rep. Prog. Phys.* **79**, 076401 (2016). 10.1088/0034-4885/79/7/076401 27308726

[55] He, C., Antonello, J. & Booth, M. J. Vectorial adaptive optics. *eLight* **3**, 23 (2023).

[56] He, C. & Booth, M. J. Vectorial adaptive optics: correction of polarization and phase. In *Imaging and Applied Optics Congress 2022 (3D, AOA, COSI, ISA, pcAOP)* OTh3B.4 10.1364/AOA.2022.OTh3B.4 (Optica Publishing Group, 2022).

[57] Lin, X. et al. All-optical machine learning using diffractive deep neural networks. *Science* **361**, 1004–1008 (2018). 10.1126/science.aat8084 30049787

[58] Zhang, X., Ezawa, M. & Zhou, Y. Magnetic skyrmion logic gates: conversion, duplication and merging of skyrmions. *Sci. Rep.* **5**, 9400 (2015). 10.1038/srep09400 PMC4371840 25802991

[59] Sisodia, N. et al. Robust and programmable logic-in-memory devices exploiting skyrmion confinement and channeling using local energy barriers. *Phys. Rev. Appl.* **18**, 014025 (2022).

[60] Yu, D., Yang, H., Chshiev, M. & Fert, A. Skyrmions-based logic gates in one single nanotrack completely reconstructed via chirality barrier. *Natl. Sci. Rev.* **9**, nwac021 (2022). 10.1093/nsr/nwac021 PMC9874028 36713589

[61] Hua, S. et al. An integrated large-scale photonic accelerator with ultralow latency. *Nature* **640**, 361–367 (2025). 10.1038/s41586-025-08786-6 PMC11981923 40205213

[62] Ahmed, S. R. et al. Universal photonic artificial intelligence acceleration. *Nature* **640**, 368–374 (2025). 10.1038/s41586-025-08854-x 40205212

[63] Sotirova, A. S. et al. Low cross-talk optical addressing of trapped-ion qubits using a novel integrated photonic chip. *Light Sci. Appl.* **13**, 199 (2024). 10.1038/s41377-024-01542-x PMC11335750 39164255

