# Supplementary material for: Topologically robust programmable processing arrays using light and matter skyrmions
Source: Nat Commun. 2026 Oct 8;17:11470. doi: 10.1038/s41467-026-78249-7 (PMC13740274; doi:10.1038/s41467-026-78249-7)
Supplement: Supplementary file 1 [file 41467_2026_78249_MOESM1_ESM.pdf]

Supplementary Information

Topologically robust programmable processing arrays using light and matter skyrmions

Ruofu Liu[1], Yunqi Zhang[1], Yuxi Cai[1], Yihan Liu[2], Zhenglin Li[1], Tianxin Wang[1], Yifei Ma[1], Zimo Zhao[1], Runchen Zhang[1], Zhi-Kai Pong[1], Stephen M. Morris[1] and Chao He[1*]

[1]Department of Engineering Science, University of Oxford, Parks Road, Oxford, OX1 3PJ, UK
[2]Tsinghua University, Haidian District, Beijing 100084, China
[*]Corresponding author: chao.he@eng.ox.ac.uk

Supplementary Note 1. The definition of Stokes skyrmions and merons

Let $\mathbf{S}(r,\theta) = (S_1, S_2, S_3)^\top$ denote the normalized Stokes field. A skyrmion with order $n \in \mathbb{Z}$ is specified by

$$\begin{bmatrix} S_1(r,\theta) \\ S_2(r,\theta) \\ S_3(r,\theta) \end{bmatrix} = \begin{bmatrix} \sqrt{1-\sigma_3(r)^2}\ \cos(n\theta) \\ \sqrt{1-\sigma_3(r)^2}\ \sin(n\theta) \\ \sigma_3(r) \end{bmatrix}, 0 \le r \le 1, 0 \le \theta < 2\pi, \qquad (1)$$

where $\sigma_3(r)$ is any smooth radial profile satisfying the boundary conditions $\sigma_3(0) = 1$ and $\sigma_3(1) = -1$. This configuration yields a topological charge of $N_{\mathrm{sk}} = n$.

To define a meron, we introduce a modified radial profile $g(r) = \frac{1+\sigma_3(r)}{2}$, which maps the domain $[0,1]$ to the range $[1,0]$, satisfying $g(0) = 1$, $g(1) = 0$. A meron of order $n \in \mathbb{Z}$ is then specified by

$$\begin{bmatrix} S_1(r,\theta) \\ S_2(r,\theta) \\ S_3(r,\theta) \end{bmatrix} = \begin{bmatrix} \sqrt{1-g(r)^2}\ \cos(n\theta) \\ \sqrt{1-g(r)^2}\ \sin(n\theta) \\ g(r) \end{bmatrix}, 0 \le r \le 1, 0 \le \theta < 2\pi. \qquad (2)$$

This texture results in a half-integer topological charge $N_{\mathrm{sk}} = n/2$.

Supplementary Note 2. The definition of skyrmion number

We follow the standard topological framework for maps between compact, connected, oriented manifolds of equal dimension. Let $f: X \to Y$ be smooth; with Y equipped with a top form ω of non-zero integral, the topological degree $\deg f$ is defined by

$$\int_X f^* \omega = (\ \deg f\ ) \int_Y \omega\,. \tag{3}$$

For optical skyrmions the target manifold is the two-sphere S^2, identified with the Poincaré sphere. The field is the normalized Stokes 3-vector

$$\mathbf{S}(x, y) \in S^2, \mathbf{S} = (S_1, S_2, S_3)^\top, \tag{4}$$

obtained from the measured Stokes parameters of a fully polarized beam.

Here, let $U \subset \mathbb{R}^2$ be a closed unit disk. We say $\mathbf{S}: U \to S^2$ is compactifiable if there exists a compact, connected, oriented manifold X and a smooth map $\phi: U \to X$ such that

(i) ϕ is an orientation-preserving diffeomorphism of U onto its image.
(ii) $\phi(U)$ is a dense subset in X with full measure.
(iii) $\mathbf{S} \circ \phi^{-1}$ extends continuously to a smooth map $\tilde{\mathbf{S}}: X \to S^2$.

Denote $\tilde{\mathbf{S}}$ as the compactification of $\mathbf{S}$. We then define the skyrmion number of $\mathbf{S}$ by $\deg \mathbf{S} := \deg \tilde{\mathbf{S}}$.

In coordinates (x, y) on U this yields the skyrmion integral

$$\deg \mathbf{S} = \int_X \tilde{\mathbf{S}}^* \omega = \int_U \phi^* (\tilde{\mathbf{S}}^* \omega) = \int_U (\tilde{\mathbf{S}} \circ \phi)^* \omega = \int_U \mathbf{S}^* \omega = \frac{1}{4\pi} \iint_U \mathbf{S} \cdot \left(\frac{\partial \mathbf{S}}{\partial x} \times \frac{\partial \mathbf{S}}{\partial y} \right) dx dy, \tag{5}$$

where the dot and cross are the Euclidean inner and vector products in $\mathbb{R}^3$. Eq. (5) is invariant under orientation-preserving reparameterizations of U and under global rotations of the Poincaré sphere (i.e., uniform retarders), and therefore provides a coordinate-free, instrument-independent definition of the optical skyrmion charge used in this work.

Supplementary Note 3. Design of the topological primitives

Each of the four primitives in our toolkit is realized by a single spatially varying retarder field, and the goal of this section is to derive the design rules that link the boundary structure of the retarder field to the change in skyrmion number it induces. We work throughout on the closed unit disk Ω with polar coordinates (r, θ), taking the device boundary at $r = 1$.

Consider a retarder field whose fast-axis distribution traces an order-n meron pattern over Ω and whose retardance varies only radially, $\delta = \delta(r)$, with boundary value $\sigma := \delta(1)$. For a uniformly right-circularly polarized input, the retarder acts at each point as a Poincaré-sphere rotation by $\delta(r)$ about the local fast axis. At the boundary, the fast axis winds n times around the equator and the rotation angle equals σ, so a direct Rodrigues calculation gives the output Stokes vector along $\partial\Omega$ as

$$\mathbf{S}_\sigma(\theta) = \begin{pmatrix} \sin(\sigma)\sin(n\theta) \\ -\sin(\sigma)\cos(n\theta) \\ \cos(\sigma) \end{pmatrix}, \tag{6}$$

a curve that traces the latitude $S_3 = \cos(\sigma)$ on the Poincaré sphere and winds around it n times as θ sweeps $\partial\Omega$ once.

To extract the skyrmion number from this boundary trace, we first note that Eq. (5) admits a geometric reading as the signed area on S^2 swept out by $\mathbf{S}(\Omega)$, normalized by 4π. The simplest extension of $\mathbf{S}_\sigma$ into the interior wraps onto the spherical cap $\{S_3 \geq \cos(\sigma)\}$ exactly n times and contributes $\frac{n}{2}(1-\cos(\sigma))$ to the integral; a general extension may additionally cover the sphere any integer number of times. The skyrmion number is therefore multi-valued as a function of the boundary, but its image under the natural projection $\mathbb{R} \to \mathbb{R}/\mathbb{Z} \cong S^1$ is single-valued. Following Refs.[1,2], we encode this boundary-to-fractional-part correspondence in a continuous map

$$\mathcal{T}: C^\infty(\partial\Omega, S^2) \longrightarrow S^1, \qquad \mathcal{T}(\Sigma) = \mathrm{e}^{2\pi\mathrm{i}\,\{N_{\mathrm{sk}}(\mathbf{S})\}}, \tag{7}$$

where $\{\cdot\}$ denotes the fractional part and $\mathbf{S}$ is any smooth extension of Σ into Ω; the integer ambiguity in N_{sk} is absorbed by the exponential, so $\mathcal{T}$ is well-defined. For the boundary curve of Eq. (6), direct substitution gives

$$\mathcal{T}(\mathbf{S}_\sigma) = \mathrm{e}^{2\pi\mathrm{i}\left\{\frac{n}{2}(1-\cos(\sigma))\right\}} = \mathrm{e}^{\pi\mathrm{i}n(1-\cos(\sigma))}. \tag{8}$$

To recover ΔN_{sk} across the full device and not merely its fractional part, we view propagation through the retarder field as a smooth homotopy. For $t \in [0,1]$, define $H: \Omega \times [0,1] \longrightarrow S^2$ by applying the retarder field with rescaled retardance $t\delta(r)$ to the input, so that $H(\cdot, 0)$ is the uniform input field and $H(\cdot, 1)$ is the full output $\mathbf{S}_\sigma$. Restricting to $\partial\Omega$ and composing with $\mathcal{T}$ yields a continuous path in S^1,

$$\mathcal{T} \circ H|_{\partial\Omega}(t) = \mathrm{e}^{\pi\mathrm{i}n(1-\cos(t\sigma))}, \tag{9}$$

whose pre-image in $\mathbb{R}$ under the universal covering map $\mathbb{R} \to S^1$, $x \mapsto \mathrm{e}^{2\pi\mathrm{i}x}$, encodes the full (non-fractional) skyrmion number along the family. By the path-lifting property of this covering, the path in Eq. (9) admits a unique continuous lift $\widetilde{N}: [0,1] \to \mathbb{R}$ once its initial

value is fixed; by Eq. (7), this lift coincides with $t \mapsto N_{\mathrm{sk}}(H(\cdot, t))$, and the initial condition $\widetilde{N}(0) = N_{\mathrm{sk}}(H(\cdot, 0)) = 0$ pins the integer offset. Evaluating at $t = 1$,

$$\Delta N_{\mathrm{sk}} = \widetilde{N}(1) - \widetilde{N}(0) = \frac{1}{2\pi} \int_0^1 (\mathcal{T} \circ H|_{\partial\Omega})^* d\varphi = \frac{n}{2}(1 - \cos(\sigma)), \tag{10}$$

where $d\varphi$ is the standard angular 1-form on S^1.

The two operating points of the toolkit follow immediately. At $\sigma = \pi/2$, Eq. (10) gives $\Delta N_{\mathrm{sk}} = n/2$, identifying the retarder field as a meron generator and as a skyrmion-to-meron interconverter. At $\sigma = \pi$, Eq. (10) gives $\Delta N_{\mathrm{sk}} = n$, identifying the same family of retarder fields as a skyrmion generator and as a skyrmion adder. The reverse direction of the converter, namely meron-to-skyrmion conversion, follows from applying the same lift argument to an incident field whose boundary is itself a meron. The register is realized by aligning the boundary fast-axis distribution of the retarder field with the incident polarization state on $\partial\Omega$. Because each boundary point then lies along the rotation axis of its local retarder, it is fixed under propagation for any retardance, and the boundary trace $H_t|_{\partial\Omega}$ is invariant in t. Consequently $\mathcal{T} \circ H_t|_{\partial\Omega}$ is constant in S^1, its lift $\widetilde{N}(t)$ is constant in $\mathbb{R}$, and $\Delta N_{\mathrm{sk}} = 0$ independently of the retardance profile $\delta(r)$.

Two design implications complete the section. First, every step in the derivation depends on the retarder field only through its restriction to $r = 1$; the homotopy that pins the integer offset enters the argument only through its continuity in t, with the spatial structure of H at intermediate t playing no role. Consequently, any perturbation of the retarder field that leaves its boundary structure intact yields the same ΔN_{sk}, and the primitives inherit a strong form of topological robustness, in close analogy with the propagation-robustness result of Ref.[3]. Second, the analysis above treats only the case of a uniformly right-circularly polarized incident boundary; generalizing to other incident fields, however, requires only minor modifications. In particular, if the boundary of the incident field is in any other polarization state, let G be a rotation matrix that maps right-circularly polarized light to that state and R be any relevant matter field described above. Then the primitive given by $GRG^{\top}$ achieves the same functionality for the altered incident boundary state (see Supplementary Note 6).

The design rules above specify each primitive individually; composing them into a multistep sequence follows directly. Since each primitive acts on the field as a Mueller matrix, a cascade of primitives transforms the input Stokes field as $\mathbf{S}_{\mathrm{out}} = M_m \cdots M_2 M_1 \mathbf{S}_{\mathrm{in}}$, the ordered product of the per-stage Mueller matrices, and the net change in skyrmion number is the sum of the per-stage contributions fixed by Eq. (10). To show all four primitives working in a single sequence, we simulated a chain combining a meron generator, a meron register, a meron-to-skyrmion converter, and a skyrmion adder (see Supplementary Fig. 1). Starting from a uniform left-circularly polarized input ($N_{\mathrm{sk}} = 0.000$), the generator produces a meron ($N_{\mathrm{sk}} = 0.500$), the register preserves it ($N_{\mathrm{sk}} = 0.500$), the converter turns it into an order-1 skyrmion ($N_{\mathrm{sk}} = 1.000$), and the adder yields a final order-5 skyrmion ($N_{\mathrm{sk}} = 5.000$), consistent with the per-stage values given by Eq. (10). The skyrmion number evolves through the predicted sequence at every stage.

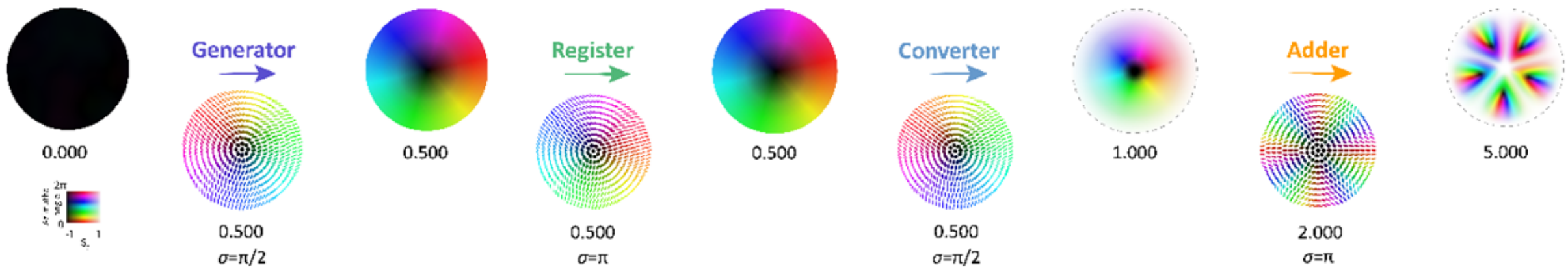

Supplementary Figure 1: Simulation of a four-stage cascade using the full primitive library. A uniform left-circularly polarized input ($N_{\mathrm{sk}} = 0.000$) is processed sequentially through a meron generator ($\sigma = \pi/2$, order 1), a meron register ($\sigma = \pi$, order 1), a meron-to-skyrmion converter ($\sigma = \pi/2$, order 1), and a skyrmion adder ($\sigma = \pi$, order 4), realizing the chain $N_{\mathrm{sk}} = 0.000$, 0.500, 0.500, 1.000, 5.000. From left to right, the input field, the fast-axis distributions of the four retarder fields, and the output Stokes field after each stage are shown in sequence. The skyrmion number is given for every field.

Supplementary Note 4. Experimental configuration and pattern synthesis

The light source was a $617\,nm$ red LED (Thorlabs M617L3). Each arbitrary elliptical retarder[4] was synthesized using three cascaded liquid-crystal-on-silicon spatial light modulators (LC-SLMs; Hamamatsu X15213-01, 1272×1024 pixels, $12.5\,\mu m$ pixel pitch, $15.9 \times 12.8\,mm$ active area), which produce the target polarization textures. The generator comprises one such triplet; for the converter, adder, and register we employ six cascaded LC-SLMs, viewing each triplet as a single arbitrary elliptical retarder, so that the first three create the target texture while the subsequent three execute the intended operation. Successive SLMs are connected by unit-magnification $(1:1)$ 4f relay systems, which image each SLM plane onto the next and keep the field in an imaging configuration throughout the cascade. The output polarization textures are imaged at unit magnification onto a monochrome CMOS camera (Basler acA4024-29um, 4024×3036 pixels, $1.85\,\mu m$ pixel pitch). In the single-channel primitive experiments (Figs. 2 and 3), each texture was imaged to fill a region of approximately 2800×2800 camera pixels, corresponding to about 414×414 SLM pixels under the unit-magnification relay. In the multichannel arrays, this same imaged region is subdivided among the channels, so that each channel occupies approximately 933×933 camera pixels (138×138 SLM pixels) in the 3×3 array and 280×280 camera pixels (41×41 SLM pixels) in the 10×10 array. Characterization of both optical and associated matter field responses is performed with a polarization state generator (PSG) and polarization state analyzer (PSA), enabling precise Mueller matrix and Stokes measurements. The full optical layout and component specifications are provided in Supplementary Fig. 2.

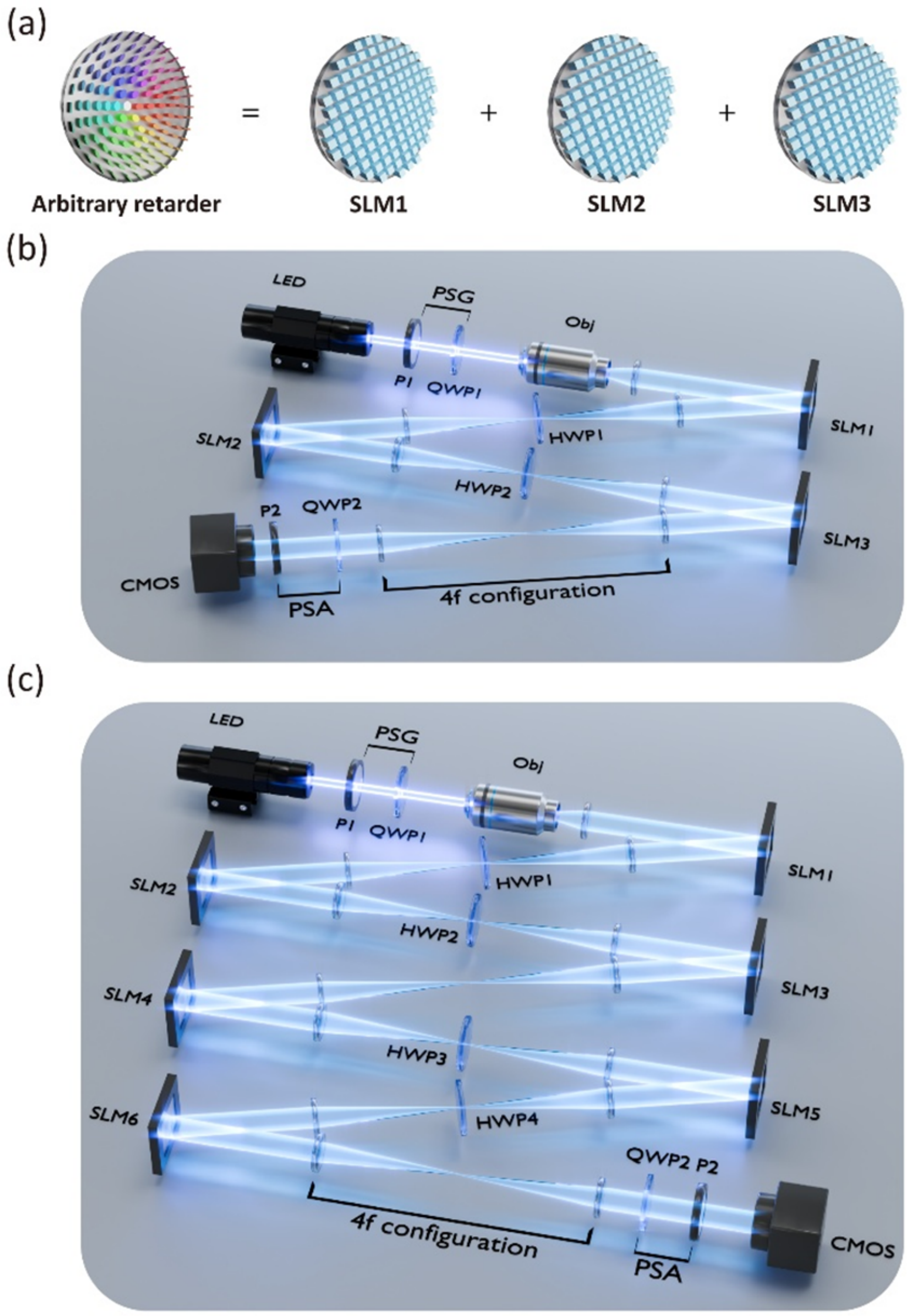

Supplementary Figure 2: Experimental setups. (a) An arbitrary retarder field implemented using three cascaded LC-SLMs. **(b)** Optical layout of the generator, comprising three cascaded LC-SLMs. **(c)** Setup for the converter, adder, and register, which employs a cascade of six LC-SLMs. In both configurations, 4f relay systems are positioned between successive SLMs to ensure precise optical mapping. The setups incorporate a PSG and a PSA for the synthesis and characterization of the target retarder field and polarization textures. HWP: half-wave plate; QWP: quarter-wave plate; P: polarizer; Obj: objective lens.

Beyond their role above, removing these 4f relays so that the field propagates freely between elements would reduce the system size but reintroduce propagation: the skyrmion number is preserved under certain free-space propagation in the paraxial, boundary-preserving regime[2], whereas outside this regime the charge can undergo controlled number transitions[5]. Whether a lens-free cascade stays within the protected regime is therefore set by how the structured field propagates, and is left to future work.

As illustrated in Supplementary Fig. 3, each operation is realized by factorizing the target Mueller matrix into three phase patterns, which are then uploaded to an SLM triplet. This modular decomposition framework is applied uniformly across all functional primitives.

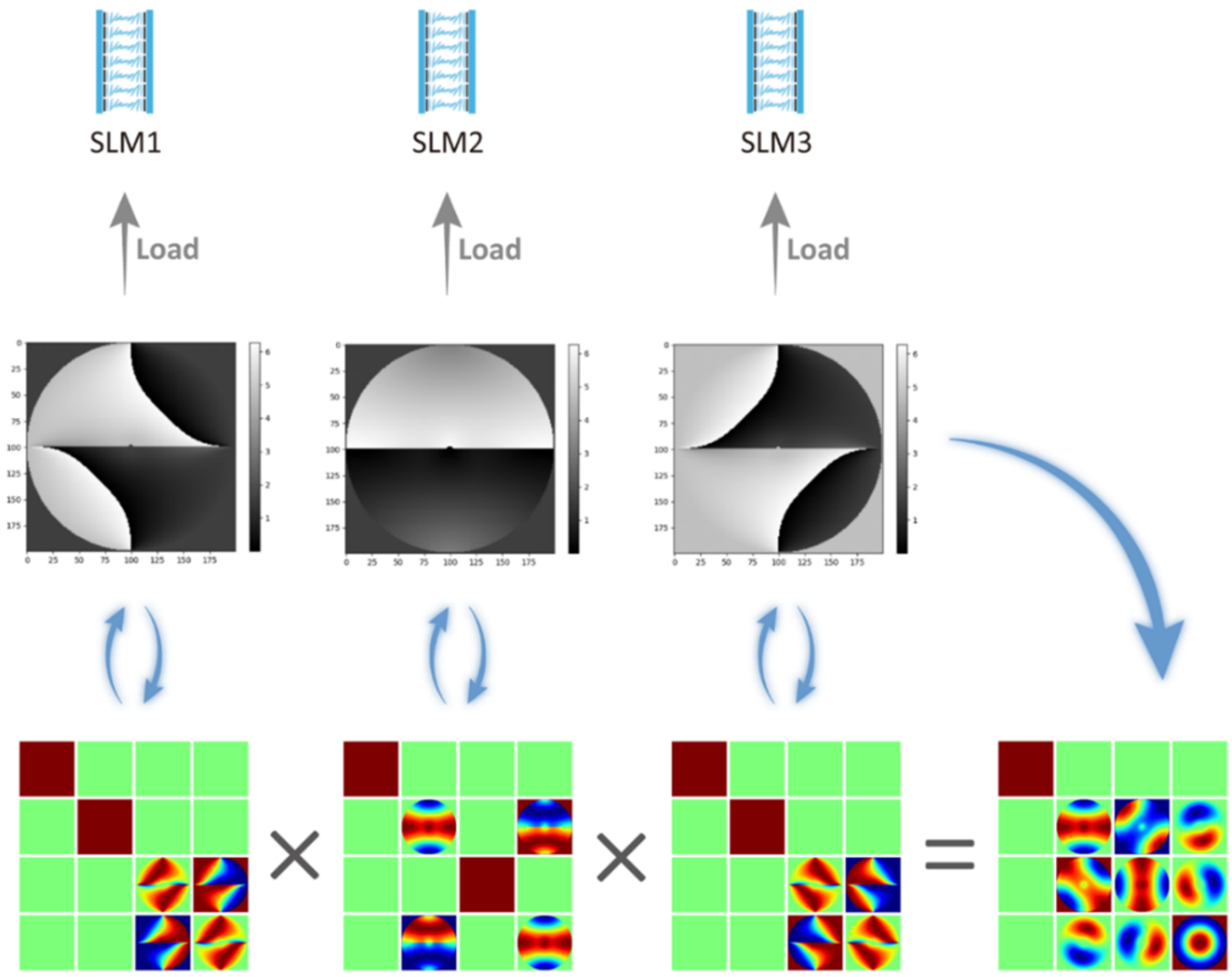

Supplementary Figure 3: LC-SLM implementation. A target Mueller matrix is factorized into three constituent Mueller matrices. These are converted into corresponding phase patterns represented as 0 to 255 grayscale values and loaded onto a triplet of LC-SLMs (SLM1, SLM2, and SLM3) to synthesize the required retarder field. This modular workflow is applied to both the generation and operation primitives.

Supplementary Note 5. Mueller matrix decomposition and retarder parametrization

We implement the Mueller matrix polar decomposition (MMPD) of Lu and Chipman[6], which we use to recover the fast-axis distribution from the measured Mueller matrix[7]. In its general form, the method factorizes a Mueller matrix into the ordered product of a diattenuator M_{D}, a retarder M_{R}, and a depolarizer M_{Δ}

$$M = M_{\mathrm{D}}\, M_{\mathrm{R}}\, M_{\Delta}. \tag{11}$$

Because our structured matter is engineered to behave as a pure retarder (no diattenuation, no depolarization), the decomposition collapses to

$$M = M_{\mathrm{R}} = \begin{bmatrix} m_{11} & 0 & 0 & 0 \\ 0 & m_{22} & m_{23} & m_{24} \\ 0 & m_{32} & m_{33} & m_{34} \\ 0 & m_{42} & m_{43} & m_{44} \end{bmatrix} = \begin{bmatrix} 1 & 0^{\top} \\ 0 & m_{\mathrm{R}} \end{bmatrix}, \tag{12}$$

where $m_{\mathrm{R}} \in SO(3)$ acts on the Stokes 3-vector as a rotation on the Poincaré sphere. Thus M_{R} encodes two physical ingredients: the retardance θ_s (rotation angle) and the fast-axis field $\mathbf{A} = (A_1, A_2, A_3)^{\top}$(fast-axis distribution).

These quantities are retrieved directly from m_{R}. Using $\mathrm{tr}(m_{\mathrm{R}}) = 1 + 2\cos\theta_s$,

$$\theta_s = \arccos\left(\frac{\mathrm{tr}(\mathrm{m_R}) - 1}{2}\right), \tag{13}$$

and from the antisymmetric part $m_{\mathrm{R}} - m_{\mathrm{R}}^{\top} = 2\sin\theta_s[\mathbf{A}]_{\times}$,

$$A_i = \frac{1}{2\sin\theta_s} \sum_{j,k=1}^{3} \varepsilon_{ijk}\, (m_{\mathrm{R}})_{jk} \;\; (i = 1,2,3), \tag{14}$$

with ε_{ijk} the Levi–Civita symbol.

Supplementary Note 6. Generalization to arbitrary incident polarization states

The definitions in Supplementary Note 1 describe representative topological textures (both skyrmions and merons) with their central states aligned to the North Pole $\mathbf{p} = (0,0,1)^{\top}$. To generate textures for an arbitrary uniform input $\mathbf{S}_{\text{in}}$, we exploit the rotational symmetry of the Poincaré sphere. Let $G \in SO(3)$ be a rotation matrix that maps the North Pole $\mathbf{p}$ to the specific input, satisfying $\mathbf{S}_{\text{in}} = G\mathbf{p}$. Physically, the generation is implemented by programming the retarder field. Let $R(r,\theta)$ denote the retarder field associated with the representative case; the target retarder field $R_{\text{tar}}(r,\theta)$ for the arbitrary input is then given by the conjugation

$$R_{\text{tar}}(r,\theta) = GR(r,\theta)G^{\top}. \tag{15}$$

Consequently, applying this field to the input $\boldsymbol{S}_{\text{in}}$ directly yields the target texture

$$\mathbf{S}_{\text{tar}}(r,\theta) = R_{\text{tar}}(r,\theta)\mathbf{S}_{\text{in}}. \tag{16}$$

This construction demonstrates that programmable anisotropy allows for the arbitrary reorientation of the topological texture, guaranteeing that the topological charge is retained.

Supplementary Note 7. Additional experimental results

This section provides further experimental characterization of various topological textures. These results supplement the data presented in the main text, covering a range of topological charges and retarder field configurations generated using our framework.

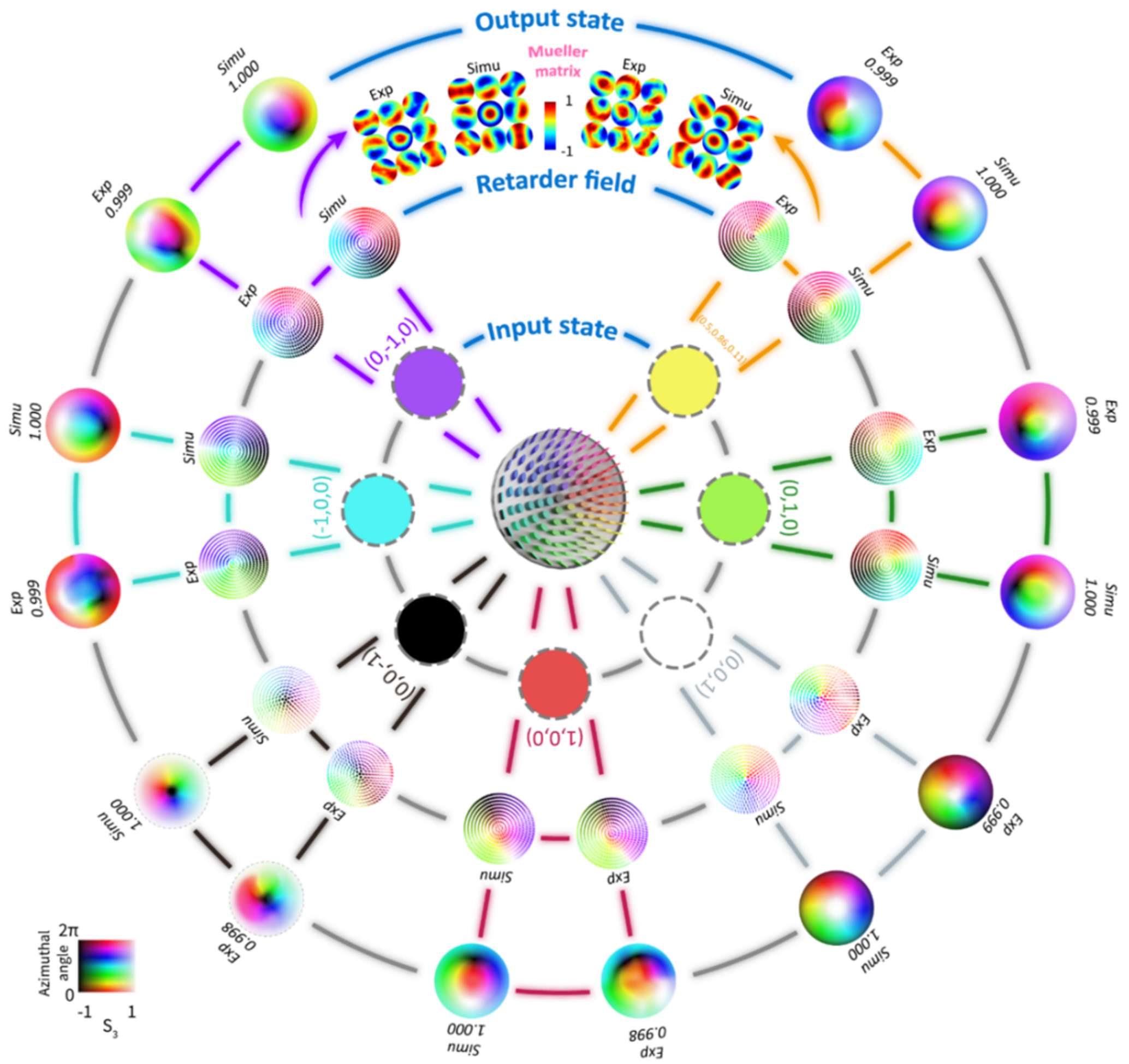

Supplementary Figure 4: Generation of skyrmions from various inputs. The figure presents a series of skyrmions with topological charge 1 produced from different uniform incident polarization states. The inner ring indicates the input state, the middle ring shows the fast-axis distribution of the retarder fields, and the outer ring represents the output skyrmions. Both simulated and experimental results are provided to show that different polarization textures can be realized within the same topological class.

The retarder-field patterns in Supplementary Fig. 4 were synthesized using 25 × 25 SLM pixels, corresponding to approximately 169 × 169 camera pixels under the unit-magnification relay, and the topological charge is still recovered correctly at this sampling.

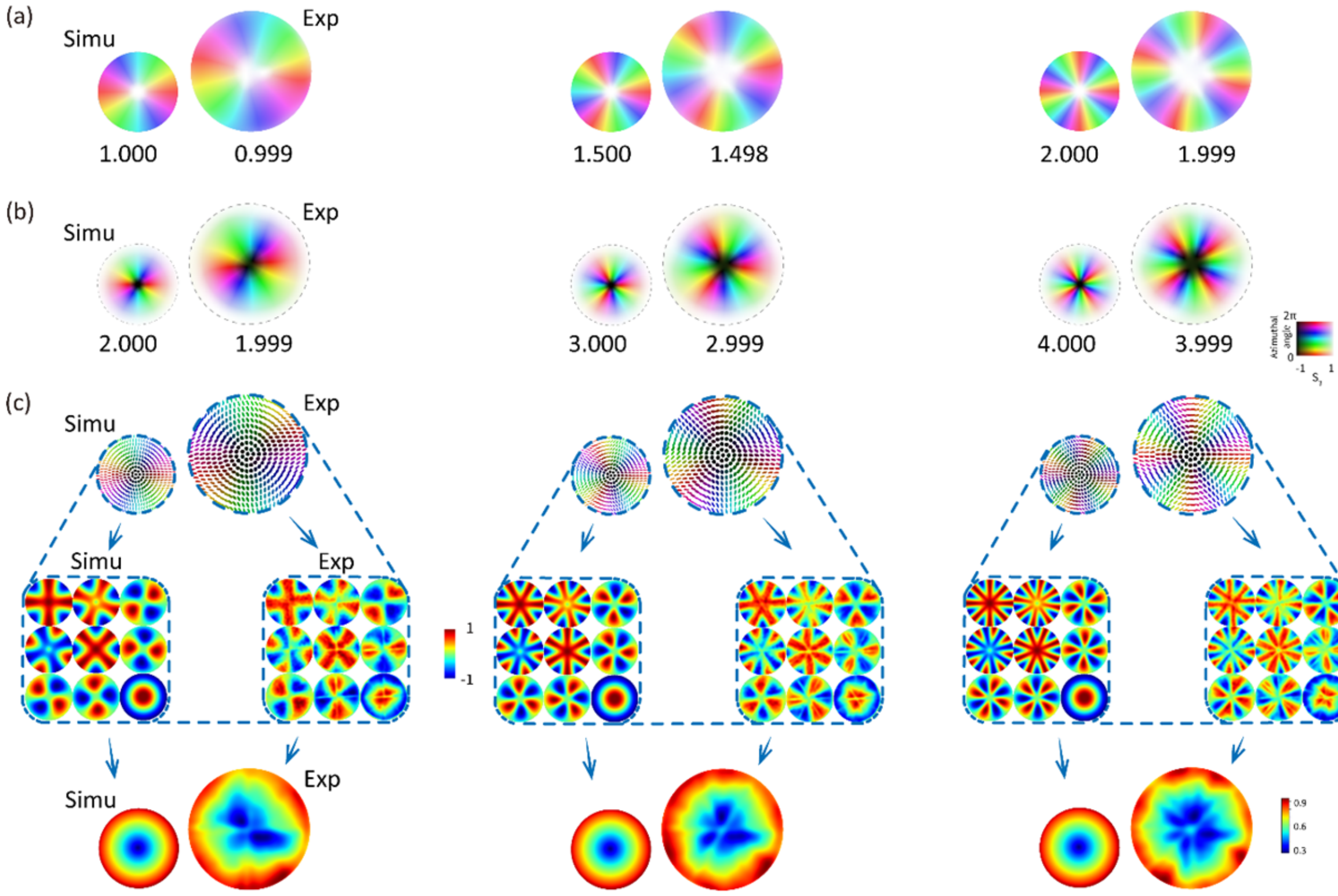

Supplementary Figure 5: Higher-order textures. (a) Simulated and experimental Stokes fields for merons of order 2, 3, and 4. **(b)** Simulated and experimental Stokes fields for skyrmions of order 2, 3, and 4. Computed topological charges are provided for each case in (a) and (b) to confirm successful generation. **(c)** Fast-axis distributions, Mueller matrices and retardance distributions for the order 2, 3, and 4 skyrmion-to-skyrmion adders introduced in the main text.

These supplementary results confirm that the operational framework maintains high fidelity even for high-order states. The accuracy of the computed charges further highlights the topological robustness of the system against experimental imperfections.

Supplementary Note 8. Topological protection of the primitives

To explicitly validate the resilience of the proposed processing architecture against perturbations, we experimentally characterized the stability of the topological charge under three distinct classes of noise, as presented in Supplementary Fig. 6. In all cases, the skyrmion generator was taken as the reference (Supplementary Fig. 6a) and the perturbation was injected directly onto the retarder field.

We first considered spatially correlated disorder that leaves the boundary unchanged. The perturbation was generated as band-limited random noise: random phases were assigned to all spatial-frequency components below a cutoff frequency $K_{\mathrm{max}} = 0.05$ (in normalized units) and transformed back to real space. The cutoff frequency was chosen to span the low-spatial-frequency band where most practical aberrations typically reside. The resulting profile is smooth and spatially correlated, and is scaled by a peak retardance Φ_{max}. Band-limited random noise of this kind is the standard model for spatially correlated disturbances, most notably atmospheric turbulence[8,9], and captures the smooth, low-spatial-frequency character shared by the dominant aberrations encountered in practice, including misalignment between cascaded elements, residual optical imperfections, and thermal or mechanical drift[10]. In the boundary-preserving case (see Supplementary Fig. 6b), the noise was modulated so as to leave the boundary of the field unperturbed while scrambling its interior. Notably, the integrated skyrmion number remains pinned at $N_{\mathrm{sk}} \approx 1.000$ across the entire range, varying by less than 0.005 (see Supplementary Data) even as Φ_{max} is increased to $20\ \mathrm{rad}$. We emphasize that this is an extreme level of disorder: a peak retardance of $20\ \mathrm{rad}$ corresponds to more than three full additional cycles of phase ($\approx 6.4\pi$) imposed locally on the field, well beyond the perturbation levels typical of practical optical systems, where aberrations usually remain at the level of a fraction of a radian[11]. That the topological charge remains intact under such a strong internal perturbation indicates that the boundary is the key to the topological protection[2].

We next applied the identical band-limited noise uniformly across the entire field, so that the boundary was no longer preserved (see Supplementary Fig. 6c). Here the skyrmion number falls monotonically with increasing Φ_{max}, reaching 0.952 at $8\ \mathrm{rad}$, 0.922 at $10\ \mathrm{rad}$, and 0.427 at $20\ \mathrm{rad}$ (see Supplementary Data), in clear contrast to the boundary-preserving case and consistent with the boundary dependence of the protection mechanism established in Supplementary Note 3. Notably, the charge remains recoverable to within 1% of unity up to $\Phi_{\mathrm{max}} = 6\ \mathrm{rad}$, itself a substantial global perturbation of nearly 2π. We note that, once the boundary is no longer preserved, the topological protection is likely to be destroyed; nonetheless, the measured skyrmion number remains stable up to this level of perturbation, beyond what is typically encountered in practice.

Finally, to probe the most stringent case of fully uncorrelated disorder, we applied pixel-wise Gaussian retardance noise across the whole field, so that the boundary was also corrupted (see Supplementary Fig. 6d). The perturbation at each pixel is drawn independently from a normal distribution of standard deviation σ_{noise}. Unlike the band-limited cases above, it has no spatial correlation and represents a worst-case stress test rather than a physically typical disturbance. We note that the two strength measures are defined differently, the band-limited cases being characterized by the peak retardance

$\Phi_{\max}$ and the Gaussian case by the standard deviation σ_{noise}, so that the horizontal axes are not directly interchangeable; nonetheless, both are expressed in radians and the qualitative comparison is unambiguous. The skyrmion number remains close to $N_{\text{sk}} \approx 1.000$ for $\sigma_{\text{noise}} \lesssim 0.25\pi\,(0.785\,\text{rad})$, begins to deviate at $\sigma_{\text{noise}} = 0.30\pi$ $(0.942\,\text{rad}, N_{\text{sk}} \approx 0.926)$, and falls thereafter, reaching 0.743 at $0.40\pi\,(1.257\,\text{rad})$ and 0.341 at $\pi\,(3.142\,\text{rad})$ (see Supplementary Data). This onset of deviation near $\sigma_{\text{noise}} \approx 0.30\pi\,(0.942\,\text{rad})$ is broadly consistent with the independently derived sufficient condition for the stability of the topological charge, the so-called $60°$ rule[7], which states that the charge is preserved provided the realized field deviates from the design everywhere by less than $60°$. The contrast with the boundary-corrupting correlated case is clear: whereas correlated disorder of $6\,\text{rad}$ leaves the charge nearly untouched, uncorrelated noise of the order of one radian is already sufficient to disrupt it. This shows the much greater destructive effect of fully uncorrelated perturbations, although such perturbations are rarely encountered in practical optical systems.

Taken together, these three experiments establish the stability of the topological charge of the proposed primitives: it withstands strong correlated disorder in the interior, yet degrades in a controlled manner once the boundary is no longer preserved, and is most sensitive to fully uncorrelated noise. Since the perturbations that dominate in real optical systems are mostly spatially correlated and typically weaker than the levels tested here, these results support the reliability of the framework under realistic operating conditions. In addition, these experiments distinguish topological protection, the invariance of the charge when the boundary is preserved (Supplementary Fig. 6b), from a more general robustness once the boundary is corrupted, where the topological protection no longer applies and the charge drifts (Supplementary Fig. 6c, d) but the readout remains correct while the perturbation stays within a certain range. Complete numerical values for all three datasets are provided in the Supplementary Data.

We also note that the same topological protection theory applies to merons, the transport states in our framework. Both skyrmions and merons belong to the fractional skyrmions defined in Ref.[2], and the topological protection theory of this class has been established in the same work: a continuous perturbation that is constant along the boundary of the field (such as the boundary-preserving disorder in Supplementary Fig. 6b) preserves the skyrmion number; applied to a meron, whose charge is $n/2$, this pins the charge at $n/2$.

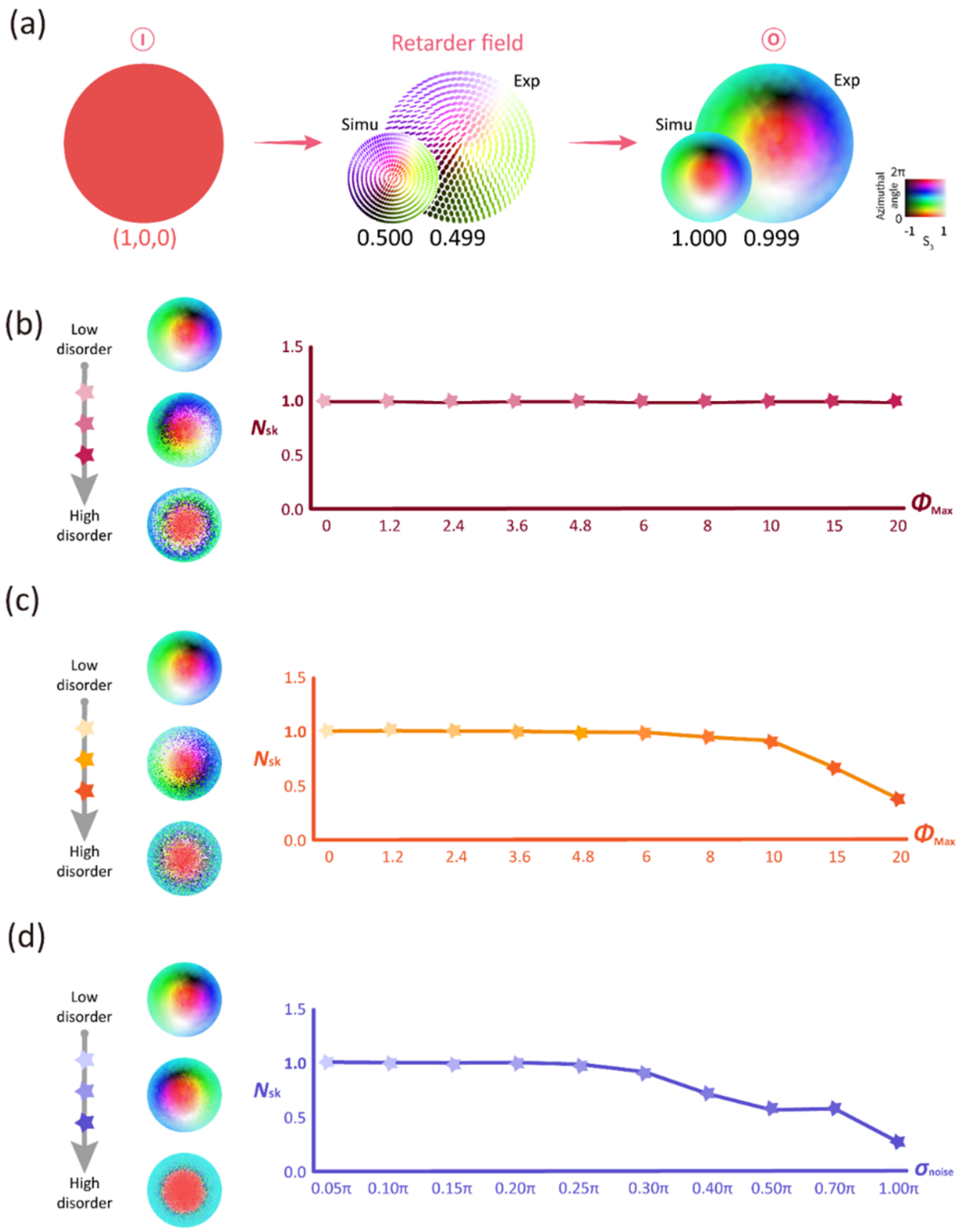

Supplementary Figure 6: Stability of the topological charge under noise. (a) Reference case: Generation of an $N_{\mathrm{sk}} = 1$ skyrmion from a horizontally polarized incident state. **(b)** Boundary-preserving, spatially correlated disorder. (Left) Representative output Stokes fields at three different noise levels, where a smooth random retardance scrambles the interior while leaving the boundary intact. (Right) Calculated skyrmion number N_{sk} across ten increasing noise levels from 0 to 20 rad. Star markers indicate the noise level, with darker colors representing higher levels of perturbation. **(c)** Boundary-corrupting, spatially correlated disorder. The same smooth random retardance applied uniformly across the entire field, so that the boundary is no longer preserved; panels follow the same layout as (b), with three representative output Stokes fields on the left and the full ten-point N_{sk} curve on the right, again spanning 0 to 20 rad. **(d)** Boundary-corrupting, fully uncorrelated Gaussian disorder. Pixel-wise uncorrelated Gaussian retardance noise applied uniformly across the field; panels follow the same layout as (b), with the noise level set by the standard deviation σ_{noise} ranging from 0 to π.

Supplementary Note 9. Large-scale demonstration

To demonstrate the scalability of the proposed framework beyond the representative 3×3 array shown in the main text (Fig. 4), we extended the architecture to a 10×10 array of independent channels. This large-scale implementation utilizes the same library of topological primitives to perform parallel generation and manipulation across 100 spatially multiplexed channels. To illustrate the functional diversity, four specific channels (circled in Supplementary Fig. 7) are highlighted; from top to bottom, these channels correspond to generator, register, register, and adder operations, respectively, with their resulting skyrmion numbers explicitly indicated. The complete results for all 100 channels are provided in the Supplementary Data.

The experimentally measured Stokes fields confirm that the framework maintains high performance even when scaled to significantly higher channel densities. Since the field fills the same camera region in every array, increasing the array from 3×3 to 10×10 reduces each channel's area by an 11-fold factor; the readout accuracy is nonetheless unchanged, yielding a maximum per-channel $|N_{\mathrm{sk,exp}} - N_{\mathrm{sk,simu}}|$ of 0.003 across all 100 channels, statistically indistinguishable from the 3×3 case (full statistics in Supplementary Note 10 and Supplementary Data).

The accurate recovery of a topological invariant places only gentle demands on spatial sampling[2]: as shown in Supplementary Note 7, the topological charge is still recovered correctly down to 25×25 SLM pixels (corresponding to about 169×169 camera pixels), well below the per-channel sampling used here (see Supplementary Note 4 for details). Beyond sampling, the topological protection of the field makes it inherently insensitive to crosstalk, leaving room for further increases in density on more compact platforms. Together, these results show that the intrinsic topological robustness persists at high channel density, making larger arrays a natural route to parallelism and increased computational power.

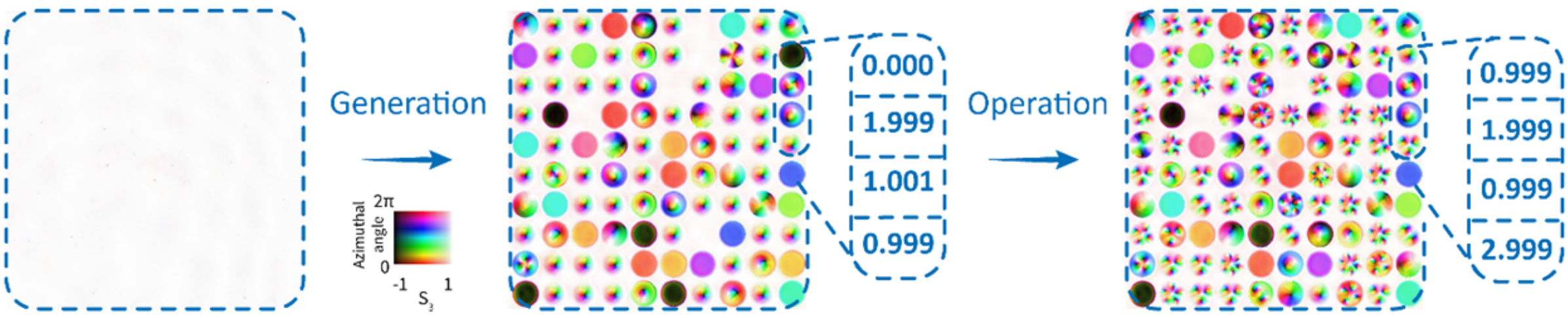

Supplementary Figure 7: Multichannel array. Experimentally measured Stokes fields of a 10×10 array of independent channels. Each channel is modulated by a different primitive in two stages, enabling rapid generation of 100 beams followed by subsequent operations on their skyrmion numbers. The skyrmion numbers of selected output units are also shown, where accurate charge readout is demonstrated.

Supplementary Note 10. Per-channel skyrmion number statistics

The per-channel readout accuracy of the 3×3 and 10×10 multichannel arrays was assessed quantitatively from the complete set of measurements reported in Supplementary Data. Per-channel deviation was defined as $|N_{\mathrm{sk,exp}} - N_{\mathrm{sk,simu}}|$, where $N_{\mathrm{sk,simu}}$ denotes the target value computed from the design and $N_{\mathrm{sk,exp}}$ denotes the value reconstructed from Stokes polarimetric measurements. Yield was defined as the fraction of channels for which the absolute deviation falls below a chosen threshold (0.01). Summary statistics are reported in Supplementary Table 1, and the spatial distribution of per-channel deviations is visualized in Supplementary Fig. 8.

**Quantity**	**3×3 array**	**10×10 array**
Number of measurements	63	300
Mean $\lvert N_{\mathrm{sk,exp}} - N_{\mathrm{sk,simu}}\rvert$	0.0009	0.0006
Std $\lvert N_{\mathrm{sk,exp}} - N_{\mathrm{sk,simu}}\rvert$	0.0007	0.0007
Max $\lvert N_{\mathrm{sk,exp}} - N_{\mathrm{sk,simu}}\rvert$	0.0030	0.0030
Yield at $\lvert\mathrm{dev}\rvert < 0.01$	100%	100%

Supplementary Table 1: Per-channel readout statistics for the 3×3 and 10×10 arrays.

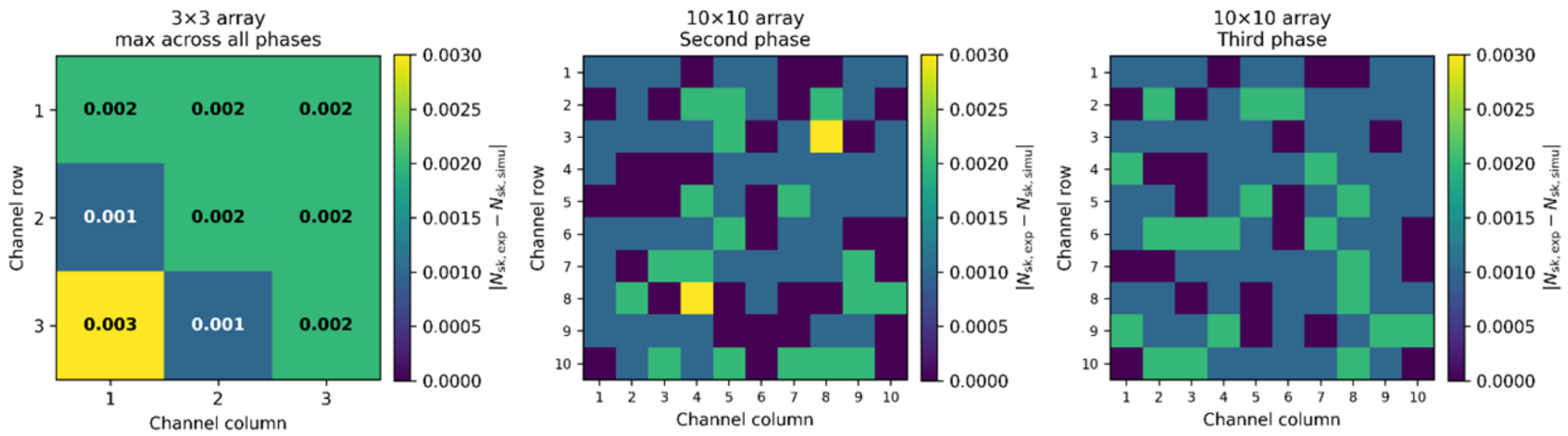

Supplementary Figure 8: Per-channel deviation heatmaps for the multichannel arrays. (a) 3×3 array: maximum $|N_{\mathrm{sk,exp}} - N_{\mathrm{sk,simu}}|$ at each spatial channel, taken across all measurements. **(b)** 10×10 array, Second phase: per-channel $|N_{\mathrm{sk,exp}} - N_{\mathrm{sk,simu}}|$. **(c)** 10×10 array, Third phase: per-channel $|N_{\mathrm{sk,exp}} - N_{\mathrm{sk,simu}}|$.

Despite an approximately 11-fold increase in channel density between the 3×3 and 10×10 arrays (since the imaged region on the SLM and camera is fixed, each channel's area shrinks by the same 11-fold factor; see Supplementary Note 4), the per-channel statistics remain essentially unchanged: for the 3×3 and 10×10 arrays respectively, the mean deviation $|N_{\mathrm{sk,exp}} - N_{\mathrm{sk,simu}}|$ is 0.0009 and 0.0006, the standard deviation is identical at 0.0007, the maximum deviation coincides at 0.003, and the yield at the $|\mathrm{dev}| < 0.01$ threshold is 100% in both cases. This consistency is also evident in the 2D deviation heatmaps (Supplementary Fig. 8), which show no pronounced systematic spatial pattern across channels; since crosstalk would produce such a pattern, this indicates that the channel crosstalk in our experiment is not significant. Underlying values are listed in Supplementary Data.

Supplementary Note 11. Implementation on alternative platforms

The retarder fields in this work are realized with an LC-SLM cascade, but several mature technologies can likewise pattern spatially varying birefringence, each subject to its own physical constraints. The noise-robustness experiments in Supplementary Note 8 relax part of these constraints for our architecture. First, the operation of each primitive is governed by the retarder field at its boundary, making it insensitive to interior imperfections. Second, the topological charge remains reliable even when the boundary itself is imperfect, provided the deviation stays within the tolerance quantified in Supplementary Note 8. Based on these two results, reproducing a primitive on another platform mainly requires the boundary to be fabricated within this tolerance, with the accuracy requirement on the interior correspondingly relaxed. We discuss three representative platforms below.

We first briefly introduce the three technologies. 1. Liquid crystal polymers form one route: a reactive mesogen is aligned into a designed director pattern, usually by photoalignment, and then fixed by polymerization into a permanent solid film that imparts a geometric phase, reaching very high efficiency with continuous phase profiles[12]. 2. Femtosecond-laser-written birefringent glasses are another: self-assembled nanogratings produce a permanent and stackable form birefringence, with the fast axis set by the writing polarization[13]. 3. Dielectric metasurfaces are a third: subwavelength structures control the phase and polarization of light, giving elements that can be made very thin[14].

We then discuss the physical constraints of each platform, and how our framework relaxes them. 1. For liquid crystal polymers, the geometric phase is set point by point by the local director orientation, so the encoded function requires director accuracy across the whole element; alignment defects tend to appear where the pattern varies rapidly or the anchoring is weak[12]. To suppress these defects, high-resolution photoalignment techniques have been developed, at the cost of more complex exposure systems and processing[12]. For our primitives, we mainly care about the director near the boundary, so the fabrication does not heavily rely on high-resolution photoalignment: defects in the interior matter much less, and the yield improves at a lower cost. 2. For femtosecond-laser-written birefringent glasses, the written retardance and fast axis drift across the device, driven by depth-dependent spherical aberration during writing[15]. To compensate for this aberration, adaptive wavefront correction has been introduced into the writing system, at the cost of extra hardware and calibration[16]. For our primitives, what matters most is the state of the boundary, so the writing does not depend strongly on adaptive wavefront correction: the drift in the interior is much less of an issue, and the fabrication becomes easier and cheaper. 3. For dielectric metasurfaces, nanometer-scale geometry errors in each meta-atom translate directly into phase errors, so the entire device must be fabricated to nanometer tolerances[14]. To relax this demand, fabrication-tolerant design strategies have been developed, at the cost of extra design effort and some loss of peak performance[17]. For our primitives, the requirement falls mainly on the meta-atoms around the boundary, so there is much less need for fabrication-tolerant design strategies: the rest of the device can be made with relaxed precision, which lowers the difficulty and cost of fabrication.

Taken together, these discussions indicate that the framework can, in most cases, be implemented on these platforms with a reduced fabrication demand.

References

1. Wang, A. A. *et al.* Generalized Skyrmions. Preprint at https://arxiv.org/abs/2409.17390 (2024).
2. Wang, A. A. *et al.* Topological protection of optical skyrmions through complex media. *Light Sci. Appl.* **13**, 314 (2024).
3. Wang, A. A. *et al.* Perturbation-resilient integer arithmetic using optical skyrmions. *Nat. Photonics* **19**, 1367–1375 (2025).
4. He, C. *et al.* A reconfigurable arbitrary retarder array as complex structured matter. *Nat. Commun.* **16**, 4902 (2025).
5. Fan, X. *et al.* Topological state and number transitions of optical skyrmions upon free-space beam propagation. *Commun. Phys.* **8**, 500 (2025).
6. Lu, S.-Y. & Chipman, R. A. Interpretation of Mueller matrices based on polar decomposition. *J. Opt. Soc. Am. A* **13**, 1106 (1996).
7. Zhang, Y. *et al.* Skyrmions based on optical anisotropy for topological encoding. *Light Sci. Appl.* **15**, 254 (2026).
8. Zhu, Z. *et al.* Compensation-free high-dimensional free-space optical communication using turbulence-resilient vector beams. *Nat. Commun.* **12**, 1666 (2021).
9. Guo, Z. *et al.* Topological robustness of classical and quantum optical skyrmions in atmospheric turbulence. *Nat. Commun.* **17**, 2085 (2026).
10. Ma, Y. *et al.* Using optical skyrmions to assess vectorial adaptive optics capabilities in the presence of complex aberrations. *Sci. Adv.* **11**, eadv7904 (2025).
11. He, C., Antonello, J. & Booth, M. J. Vectorial adaptive optics. *eLight* **3**, 23 (2023).
12. Jiang, M. *et al.* Low *f*-Number Diffraction-Limited Pancharatnam–Berry Microlenses Enabled by Plasmonic Photopatterning of Liquid Crystal Polymers. *Adv. Mater.* **31**, 1808028 (2019).
13. Beresna, M., Gecevičius, M., Kazansky, P. G. & Gertus, T. Radially polarized optical vortex converter created by femtosecond laser nanostructuring of glass. *Appl. Phys. Lett.* **98**, 201101 (2011).
14. Chen, H.-T., Taylor, A. J. & Yu, N. A review of metasurfaces: physics and applications. *Rep. Prog. Phys.* **79**, 076401 (2016).
15. Lu, J. *et al.* Tailoring chiral optical properties by femtosecond laser direct writing in silica. *Light Sci. Appl.* **12**, 46 (2023).
16. Bisch, N., Guan, J., Booth, M. J. & Salter, P. S. Adaptive optics aberration correction for deep direct laser written waveguides in the heating regime. *Appl. Phys. A* **125**, 364 (2019).
17. Wang, E. W., Sell, D., Phan, T. & Fan, J. A. Robust design of topology-optimized metasurfaces. *Opt. Mater. Express* **9**, 469 (2019).
