# Supplementary material for: Topologically robust programmable processing arrays using light and matter skyrmions
Source: Nat Commun. 2026 Oct 8;17:11470. doi: 10.1038/s41467-026-78249-7 (PMC13740274; doi:10.1038/s41467-026-78249-7)
Supplement: Supplementary file 2 [file 41467_2026_78249_MOESM2_ESM.pdf]

nature portfolio

Peer Review File

Topologically robust programmable processing arrays using light and matter skyrmions

Corresponding Author: Professor Chao He

This file contains all reviewer reports in order by version, followed by all author rebuttals in order by version.

Version 0:

Reviewer comments:

Reviewer #1

(Remarks to the Author)
General comments
This manuscript proposes a modular architecture for skyrmion-based optical logic using cascades of spatially varying retarder fields. The authors introduce four primitives (generator, converter, register, and adder) and validate them experimentally with LC-SLM cascades and Mueller-matrix polarimetry. They further present 3 × 3 and 10 × 10 multichannel demonstrations intended to support parallel operation and architectural reusability.

The manuscript has several clear strengths. First, it attempts to move the field beyond isolated demonstrations of skyrmion generation toward a more architectural language of reusable primitives. Second, the experiments are thoughtfully executed, and the measured Stokes fields agree well with simulations for the representative cases shown. Third, the work is well aligned with the recent interest in using topological invariants as robust information carriers in photonic processing.

At the same time, I do not think the current version is yet ready for acceptance in Nature Communications. In my view, the manuscript has clear potential for the journal, but it presently overstates its advance in three areas: novelty relative to the authors' own immediate prior work and closely related literature, robustness beyond the boundary-preserving regime that is actually tested, and scalability/crosstalk performance in the multichannel demonstrations. I therefore recommend major revision.
Comments:
1.The real contribution of the present manuscript, as I understand it, is the move to an architecture-level integration: a unified primitive library, the skyrmion/meron role separation, functional reuse of similar underlying retarder fields, and the reconfigurable-generation/fixed-processing split. At present, however, this distinction is not articulated sharply enough. The paper would be much stronger if the authors explicitly positioned the manuscript against the most immediate prior art and stated, in concrete terms, what is new here beyond those earlier results.

2.One of the main selling points of the paper is topological robustness. However, the actual robustness experiment (Fig. 5) is a very specific and theoretically favorable test: the authors inject strong internal disorder while explicitly preserving the boundary through a radial envelope proportional to (1−r2). This is a good and meaningful validation of the boundary-based protection mechanism, but it does not directly validate robustness against generic misalignment, boundary corruption, temperature drift, or environmental instability. I therefore suggest that the authors do one of the following: (a) provide direct quantitative tests for at least one additional practically relevant perturbation class, such as alignment offset, boundary perturbation, or temporal/thermal drift; or (b) significantly narrow the wording in the Abstract, Results, and Discussion so that the robustness claims match the regime actually demonstrated, namely boundary-preserving internal disorder.

3.The 3 × 3 and 10 × 10 demonstrations are visually compelling, but the evidence for "negligible crosstalk" and "scalable foundation" remains largely qualitative. For an architecture-oriented paper in Nature Communications, representative images and a few selected charge readouts are not sufficient to establish scalability. The authors should provide more quantitative statistics for the multichannel demonstrations. Useful additions would include, for example, channel-by-channel distributions of the measured skyrmion number, mean, and standard deviation across the array; worst-case deviations; a quantitative crosstalk metric; or a yield/accuracy map for all channels (e.g., a 2D deviation heatmap). If such data are already available, the revision may mainly require better presentation; if not, then the current wording could be toned down accordingly.

4.The manuscript repeatedly emphasizes that the primitive library can be cascaded into more complex logic. However, most experimental demonstrations still consist of two-stage operations. For a manuscript whose main claim is architectural, the case would be considerably stronger if the authors could include at least one more nontrivial composed example, such as generation →conversion → register → addition, or an equivalent multistep topological processing chain. Even if a full experimental realization is too demanding for a revision, a rigorous simulated demonstration together with a clear synthesis rule would still strengthen the architecture-level message.

Minor comments
1.In Method 1, the manuscript states that when $\sigma = \pi/2$, one has $\Delta N_{sk} = 0.5n$, which is consistent with the role of meron generators and skyrmion-to-meron converters. However, the manuscript then appears to state the same result, $\Delta N_{sk} = 0.5n$, also for $\sigma = \pi$, while simultaneously interpreting this case as skyrmion generators and skyrmion-to-skyrmion adders. I suspect this is a typo or a notational issue, but because it sits at the center of the theoretical framework, it must be corrected explicitly. The authors should carefully check the relevant expressions and ensure that all downstream descriptions remain consistent.
2.The term “register” seems somewhat stronger than the evidence shown. The experiments demonstrate topology-preserving meron transport/relay. If the authors wish to use the word register, they should clarify that this is an architectural abstraction rather than a demonstration of temporal storage in the conventional sense.
3.Likewise, the phrase “programmable logic array” may overstate the present scope. The paper demonstrates topological state generation, conversion, transport, and arithmetic, but not Boolean completeness, fan-in/fan-out, or a full digital logic abstraction. The terminology should be calibrated accordingly.
4.Since the paper relies heavily on the distinction between skyrmions and merons for function allocation, the Discussion would benefit from a clearer explanation of why this separation is advantageous beyond the pixel-resolution argument already mentioned.
5.The discussion regarding the transferability of the framework to diverse technologies appears overly simplified. While topological robustness relaxes certain fabrication errors, the authors should provide a brief qualitative analysis of the unique physical constraints inherent to each specific technological implementation, and concretely discuss the feasibility of such extensions.

Reviewer #2

(Remarks to the Author)
In the manuscript titled “Topologically robust programmable logic arrays using light and matter skyrmions” by Liu et al., the authors propose a scheme for implementing arithmetic operations using optical skyrmions and merons. Building upon their previous research, the study utilises retarders with engineered spatial profiles as key components for the generation and conversion of skyrmionic fields. A notable advancement from earlier work is the distinct roles assigned to skyrmions and merons; specifically, the conversion of skyrmions into merons and the preservation of meron states when operations are not required. Furthermore, the authors demonstrate that multiple channels can be integrated into a single optical element for parallel transmission.
I find that this work has the potential to broaden the applications of optical skyrmions and offer new directions for optical information processing. The presented data are technically sound, and the manuscript is well-organised overall. However, before recommending publication in Nature Communications, I would like the authors to address the specific concerns outlined below.

Comment #1:
Whilst the authors claim that the present approach increases design flexibility, I remain somewhat unconvinced regarding the necessity of the additional complexity involved in converting skyrmions into merons. Could the authors clarify whether a retardance profile could instead be implemented to preserve the skyrmion number whilst remaining in the skyrmion mode?

Comment #2:
Whilst integrating multiple channels onto a single optical element may be useful, the current methodology is essentially synonymous with employing multiple separate SLMs, and the results are therefore predictable. For instance, if the integration density were to be increased, the area available to each channel would necessarily decrease; to what extent would this be acceptable? At first glance, one would expect crosstalk with adjacent channels to increase significantly. Furthermore, as beam divergence would become more pronounced, would this not complicate the configuration of the 4f system, as illustrated in the supplementary material?

Comment #3:
Following on from my previous point, the current setup necessitates a 4f configuration for each retarder. I suspect that the overall system size is constrained by the focal lengths of the lenses, which may hinder the achievement of a compact design. Would it not be possible to cascade the retarders without intervening lenses, similar to passing the beam through an HWP and QWP in direct sequence? Whilst I anticipate that the results would be distorted due to diffraction, I should like the authors to clarify whether such effects might be permissible.

Comment #4:
Throughout the manuscript, simulation results are superimposed onto the experimental data, thereby obscuring portions of the primary results. I consider this practice inappropriate as it compromises the verifiability of the experimental findings. I request that the experimental results be presented in a manner that ensures they are fully visible. For instance, the

experimental result for the adder in Fig. 3b (specifically the 2.995 case) appears to be white (S_3 ~ 1) just before it is obscured by the simulation overlay; this suggests a distribution that may not align with the simulation. However, a thorough verification is currently impossible because the majority of the skyrmion on the left remains hidden.

Comment #5:
Following on from my previous point, do these plot ranges represent the integration domains used for calculating the skyrmion number? For instance, the converter results in Fig. 3b yield a remarkably ideal skyrmion number of 0.999; however, S_3 clearly does not appear to reach 1 at the boundaries. Whilst this may be an issue with the colour function, I should like to know the precise value of S_3 at the boundary. Furthermore, does the measurement system possess sufficient significant figures to justify a result of 0.999? Additionally, could the authors clarify how many pixels a single skyrmion spans in their experimental data?

Comment #6:
Finally, purely out of curiosity, I wonder whether it would be possible to transfer or mix data between channels, rather than simply processing them in parallel. For instance, could this be achieved by inserting a diffractive element or similar component into the optical path? If such inter-channel interaction were feasible, it would allow for more sophisticated calculations, which in my view would further enhance the value and impact of the proposed scheme.

Version 1:

Reviewer comments:

Reviewer #1

(Remarks to the Author)
In my opinion, the authors have satisfactorily addressed my questions and concerns. I recommend publication of the work.

Reviewer #2

(Remarks to the Author)
I would like to thank the authors for their detailed responses and for the revisions made to the manuscript. It appears that the authors have addressed the majority of the comments raised by the reviewers in the previous round. The accessibility of the manuscript has been improved, and the novelty of the work is now presented more clearly. In particular, the addition of the topological protection experiment and the per-channel deviation experiment has substantially strengthened the case for the advantages of the proposed scheme. I now believe that the work merits publication in Nature Communications, although several minor and optional revisions may be considered before publication.

The authors have revised the colour mapping of the Stokes parameters in Fig. 4 and elsewhere to improve the visibility of the near-uniform boundaries of the skyrmions. However, the saturation/value mapping of the new colour function is not linear with respect to S_3, and the region corresponding to S_3 > 0.9 is almost entirely mapped to white, making it difficult to distinguish between different physical states based on colour. With a standard 8-bit-per-channel colour representation, the values appear to become effectively indistinguishable once S_3 exceeds 0.9. Whilst this may improve the visual appearance of the figures, I believe that it compromises the accuracy of the data representation to some extent. I therefore strongly recommend that the authors readjust the colour function, for instance, so as to provide a resolution of at least 0.01 in S_3 across the relevant range.

The authors have also added an additional experiment to demonstrate the topological resilience of optical skyrmions. The result itself is convincing, however, I am curious as to whether this experiment directly demonstrates the robustness of the proposed information-processing scheme. The authors use skyrmions to demonstrate topological resilience, whereas the proposed system utilises merons for information transport. Though the experimentally demonstrated structure appears to be a bimeron configuration, which is topologically equivalent to a skyrmion under a suitable rotation of the order-parameter axis. Thus, the demonstrated topological protection is fundamentally associated with the skyrmion topology rather than with the meron character itself.
This raises the question of whether topological protection of the merons is actually necessary for the proposed scheme. In the absence of the uniform boundaries characteristic of skyrmions, the domain of an individual meron is not compactifiable in the same manner, and therefore the direct topological protection arising from the homotopy of the skyrmion texture does not apply straightforwardly. On the other hand, the results for skyrmions without boundary protection already exhibit a certain degree of robustness against perturbations, suggesting that the robustness of the information-carrying states may not necessarily rely on strict topological protection.
I therefore recommend that the authors clarify this point. In particular, it would be useful to discuss whether the robustness demonstrated in the new experiment is expected to carry over to the merons used for information transport, and, more fundamentally, whether topological protection of the merons is required for the reliable operation of the proposed scheme. Clarifying this distinction between topological protection and the more general robustness of the optical states would, in my view, make the argument for the robustness of the proposed system considerably more convincing.

Version 2:

Reviewer comments:

Reviewer #2

(Remarks to the Author)
The manuscript in its present form is clear and accessible. I believe this work opens up a new avenue for information processing using topologically structured light, and I recommend publication in Nature Communications.

Open Access This Peer Review File is licensed under a Creative Commons Attribution 4.0 International License, which permits use, sharing, adaptation, distribution and reproduction in any medium or format, as long as you give appropriate credit to the original author(s) and the source, provide a link to the Creative Commons license, and indicate if changes were made.
In cases where reviewers are anonymous, credit should be given to 'Anonymous Referee' and the source.
The images or other third party material in this Peer Review File are included in the article's Creative Commons license, unless indicated otherwise in a credit line to the material. If material is not included in the article's Creative Commons license and your intended use is not permitted by statutory regulation or exceeds the permitted use, you will need to obtain permission directly from the copyright holder.
To view a copy of this license, visit https://creativecommons.org/licenses/by/4.0/

Response to Reviewers

Manuscript Number: NCOMMS-26-025583

Title: Topologically robust programmable processing arrays using light and matter skyrmions

We sincerely thank both reviewers for their constructive and positive feedback, which we believe has significantly enhanced the quality and clarity of our manuscript.

Our responses are provided below in blue following the comments raised by each reviewer (shown in italics). We provide a **Point-to-Point Reply to All Comments of Reviewers**, which we hope will be beneficial in clarifying the main changes made to the manuscript and addressing overarching points raised during the review. Revisions to the Main article and Supplementary Notes are indicated in **Purple** text in this response below.

<u>Reply to Reviewer #1</u>

This manuscript proposes a modular architecture for skyrmion-based optical logic using cascades of spatially varying retarder fields. The authors introduce four primitives (generator, converter, register, and adder) and validate them experimentally with LC-SLM cascades and Mueller-matrix polarimetry. They further present 3 × 3 and 10 × 10 multichannel demonstrations intended to support parallel operation and architectural reusability.

The manuscript has several clear strengths. First, it attempts to move the field beyond isolated demonstrations of skyrmion generation toward a more architectural language of reusable primitives. Second, the experiments are thoughtfully executed, and the measured Stokes fields agree well with simulations for the representative cases shown. Third, the work is well aligned with the recent interest in using topological invariants as robust information carriers in photonic processing.

At the same time, I do not think the current version is yet ready for acceptance in Nature Communications. In my view, the manuscript has clear potential for the journal, but it presently overstates its advance in three areas: novelty relative to the authors' own immediate prior work and closely related literature, robustness beyond the boundary-preserving regime that is actually tested, and scalability/crosstalk performance in the multichannel demonstrations. I therefore recommend major revision.

We sincerely thank the reviewer for their careful reading of our manuscript and for their constructive evaluation of our work. In particular, we are grateful to the reviewer for highlighting that our manuscript attempts to "move the field beyond isolated demonstrations of skyrmion generation toward a more architectural language of reusable primitives", and that the work is "well aligned with the recent interest in using topological invariants as robust information carriers in photonic processing". We are also grateful for the reviewer's view that the manuscript has clear potential for *Nature Communications*.

We apologize that the previous version fell short in its presentation of these three areas. We have therefore carefully revised the manuscript to address each of the reviewer's concerns, with details provided below in a point-by-point manner.

Comment 1: *The real contribution of the present manuscript, as I understand it, is the move to an architecture-level integration: a unified primitive library, the skyrmion/meron role separation, functional reuse of similar underlying retarder fields, and the reconfigurable-generation/fixed-processing split. At present, however, this distinction is not articulated sharply enough. The paper would be much stronger if the authors explicitly positioned the manuscript against the most immediate prior art and stated, in concrete terms, what is new here beyond those earlier results.*

Response 1: Many thanks for the comment. We agree that the most immediate prior art was not explicitly positioned, and that the key novelty of this study was not stated in sufficiently concrete terms. To address this point, we have revised two major parts of the manuscript.

1. First, in the Introduction we revised the related paragraph to survey what recent works on skyrmion manipulation have achieved, including our own most immediate prior work on skyrmion-based arithmetic [1], and then clarify what remains unresolved. The revised text reads:

"Despite these advances, from a functional perspective, most studies still focus on generating specific types of optical skyrmions, such as fixed Néel-type [2][3] or Bloch-type [4] textures, or focus on complex beams themselves as input and output [5]. A sophisticated framework for matter field design that prescribes how to generate textures of arbitrary type and order remains lacking. Generation alone, moreover, is not enough: to use skyrmions as information carriers one must also manipulate the generated textures and perform programmable operations on their topological charge. Recent works have begun to do this, securing topological resilience in paraxial classical [6] and non-local quantum [7][8] skyrmions, exploiting skyrmionic textures for noise-tolerant information transfer and readout [9][10], generalizing the skyrmion number to higher-dimensional integer tuples to increase encoding capacity [11], and, most recently, modifying the skyrmion number in a perturbation-resilient way without external energy to realize integer arithmetic [1]. In each, however, the skyrmion mostly serves as a single device-level resource, with each function realized on its own; how such functions can be organized, reused and scaled has been less explored. In particular, multichannel, parallel processing remains an important open goal for a scalable architecture."

2. Second, the Introduction now states directly what the present work contributes beyond these previous studies. The relevant text reads:

"Taken together, these considerations motivate the central question addressed in this paper: how can we devise a broadly applicable framework for the programmable manipulation of polarization textures, encompassing both their generation and a library of topological processing operations, and ultimately extend these concepts to multichannel, parallel architectures? In this work, we aim to address this by developing an architecture-level framework for topological processing."

1. *A. A. Wang, Y. Ma, Y. Zhang, Z. Zhao, Y. Cai, X. Qiu, B. Dong, C. He, Perturbation-resilient integer arithmetic using optical skyrmions. Nat. Photonics 19, 1367-1375 (2025).*
2. *S. Tsesses, E. Ostrovsky, K. Cohen, B. Gjonaj, N. H. Lindner, G. Bartal, Optical skyrmion lattice in evanescent electromagnetic fields. Science 361, 993-996 (2018).*
3. *X. Lei, A. Yang, P. Shi, Z. Xie, L. Du, A. V. Zayats, X. Yuan, Photonic Spin Lattices: Symmetry Constraints for Skyrmion and Meron Topologies. Phys. Rev. Lett. 127, 237403 (2021).*
4. *S. Gao, F. C. Speirits, F. Castellucci, S. Franke-Arnold, S. M. Barnett, J. B. Götte, Paraxial skyrmionic beams. Phys. Rev. A 102, 053513 (2020).*
5. *C. He et al., A reconfigurable arbitrary retarder array as complex structured matter. Nat. Commun. 16, 4902 (2025).*
6. *A. A. Wang, Z. Zhao, Y. Ma, Y. Cai, R. Zhang, X. Shang, Y. Zhang, J. Qin, Z.-K. Pong, T. Marozsák, B. Chen, H. He, L. Luo, M. J. Booth, S. J. Elston, S. M. Morris, C. He, Topological protection of optical skyrmions through complex media. Light Sci. Appl. 13, 314 (2024).*
7. *P. Ornelas, I. Nape, R. de Mello Koch, A. Forbes, Non-local skyrmions as topologically resilient quantum entangled states of light. Nat. Photonics 18, 258-266 (2024).*
8. *P. Ornelas, I. Nape, R. de Mello Koch, A. Forbes, Topological rejection of noise by quantum skyrmions. Nat. Commun. 16, 2934 (2025).*
9. *C. Peters, K. Everts, T. Kleine, P. Ornelas, A. Forbes, Seeing through randomness with topological light. arXiv:2508.12305 (2025).*
10. *H. Bezuidenhout, C. Peters, R. Kumar, A. Forbes, I. Nape, Deep diffractive optical neural networks for detecting skyrmionic topologies of light. arXiv:2512.11115 (2025).*
11. *A. A. Wang et al., Generalized skyrmions. arXiv:2409.17390 (2024).*

Comment 2: *One of the main selling points of the paper is topological robustness. However, the actual robustness experiment (Fig. 5) is a very specific and theoretically favorable test: the authors inject strong internal disorder while explicitly preserving the boundary through a radial envelope proportional to (1−r2). This is a good and meaningful validation of the boundary-based protection mechanism, but it does not directly validate robustness against generic misalignment, boundary corruption, temperature drift, or environmental instability. I therefore suggest that the authors do one of the following: (a) provide direct quantitative tests for at least one additional practically relevant perturbation class, such as alignment offset, boundary perturbation, or temporal/thermal drift; or (b) significantly narrow the wording in the Abstract, Results, and Discussion so that the robustness claims match the regime actually demonstrated, namely boundary-preserving internal disorder.*

Response 2: We thank the reviewer for this insightful comment. We acted on both of the reviewer's suggestions: we ran additional perturbation experiments (option a), and we narrowed the wording to match the regime actually tested (option b).

1. For option (a), we expanded the study from one perturbation class to three. The three differ in two key physical respects: whether the boundary is preserved or corrupted, and whether the disorder is spatially correlated or pixel-wise random. Alongside the original boundary-preserving correlated case (Fig. 6b), we added a boundary-corrupting correlated case (Fig. 6c), as the reviewer required, and a boundary-corrupting, fully uncorrelated Gaussian case as a worst-case stress test (Fig. 6d). Methods 2 now includes all three perturbation classes and reads:

"To explicitly validate the resilience of the proposed processing architecture against perturbations, we experimentally characterized the stability of the topological charge under three distinct classes of noise, as presented in Fig. 6. In all cases, the skyrmion generator was taken as the reference (Fig. 6a) and the perturbation was injected directly onto the retarder field.

We first considered spatially correlated disorder that leaves the boundary unchanged. The perturbation was generated as band-limited random noise: random phases were assigned to all spatial-frequency components below a cutoff frequency $K_{\max} = 0.05$ (in normalized units) and transformed back to real space. The cutoff frequency was chosen to span the low-spatial-frequency band where most practical aberrations typically reside. The resulting profile is smooth and spatially correlated, and is scaled by a peak retardance $\Phi_{\max}$. Band-limited random noise of this kind is the standard model for spatially correlated disturbances, most notably atmospheric turbulence [12][13], and captures the smooth, low-spatial-frequency character shared by the dominant aberrations encountered in practice, including misalignment between cascaded elements, residual optical imperfections, and thermal or mechanical drift [14]. In the boundary-preserving case (see Fig. 6b), the noise was modulated so as to leave the boundary of the field unperturbed while scrambling its interior. Notably, the integrated skyrmion number remains pinned at $N_{\mathrm{sk}} \approx 1.000$ across the entire range, varying by less than 0.005 (see Supplementary Data) even as $\Phi_{\max}$ is increased to 20 rad. We emphasize that this is an extreme level of disorder: a peak retardance of 20 rad corresponds to more than three full additional cycles of phase ($\approx 6.4\pi$) imposed locally on the field, well beyond the perturbation levels typical of practical optical systems, where aberrations usually remain at the level of a fraction of a radian [15]. That the topological charge remains intact under such a strong internal perturbation

indicates that the boundary is the key to the topological protection [6].

We next applied the identical band-limited noise uniformly across the entire field, so that the boundary was no longer preserved (see Fig. 6c). Here the skyrmion number falls monotonically with increasing $\Phi_{\max}$, reaching 0.952 at $8\,\mathrm{rad}$, 0.922 at $10\,\mathrm{rad}$, and 0.427 at $20\,\mathrm{rad}$ (see Supplementary Data), in clear contrast to the boundary-preserving case and consistent with the boundary dependence of the protection mechanism established in Methods 1. Notably, the charge remains recoverable to within 1% of unity up to $\Phi_{\max} = 6$ rad, itself a substantial global perturbation of nearly 2π. We note that, once the boundary is no longer preserved, the topological protection is likely to be destroyed; nonetheless, the measured skyrmion number remains stable up to this level of perturbation, beyond what is typically encountered in practice.

Finally, to probe the most stringent case of fully uncorrelated disorder, we applied pixel-wise Gaussian retardance noise across the whole field, so that the boundary was also corrupted (see Fig. 6d). The perturbation at each pixel is drawn independently from a normal distribution of standard deviation σ_{noise}. Unlike the band-limited cases above, it has no spatial correlation and represents a worst-case stress test rather than a physically typical disturbance. We note that the two strength measures are defined differently, the band-limited cases being characterized by the peak retardance $\Phi_{\max}$ and the Gaussian case by the standard deviation σ_{noise}, so that the horizontal axes are not directly interchangeable; nonetheless, both are expressed in radians and the qualitative comparison is unambiguous. The skyrmion number remains close to $N_{\mathrm{sk}} \approx 1.000$ for $\sigma_{\mathrm{noise}} \lesssim 0.25\pi$ $(0.785\,\mathrm{rad})$, begins to deviate at $\sigma_{\mathrm{noise}} = 0.30\pi$ $(0.942\,\mathrm{rad}, N_{\mathrm{sk}} \approx 0.926)$, and falls thereafter, reaching 0.743 at 0.40π $(1.257\ \mathrm{rad})$ and 0.341 at π $(3.142\ \mathrm{rad})$ (see Supplementary Data). This onset of deviation near $\sigma_{\mathrm{noise}} \approx 0.30\pi\,(0.942\ \mathrm{rad})$ is broadly consistent with the independently derived sufficient condition for the stability of the topological charge, the so-called 60° rule [16], which states that the charge is preserved provided the realized field deviates from the design everywhere by less than $60°$. The contrast with the boundary-corrupting correlated case is clear: whereas correlated disorder of 6 rad leaves the charge nearly untouched, uncorrelated noise of the order of one radian is already sufficient to disrupt it. This shows the much greater destructive effect of fully uncorrelated perturbations, although such perturbations are rarely encountered in practical optical systems.

Taken together, these three experiments establish the stability of the topological charge of the proposed primitives: it withstands strong correlated disorder in the interior, yet degrades in a controlled manner once the boundary is no longer preserved, and is most sensitive to fully uncorrelated noise. Since the perturbations that dominate in real optical systems are mostly spatially correlated and typically weaker than the levels tested here, these results support the reliability of the framework under realistic operating conditions. Complete numerical values for all three datasets are provided in the Supplementary Data.”

The revised Fig. 6 is reproduced below for the reviewer's convenience:

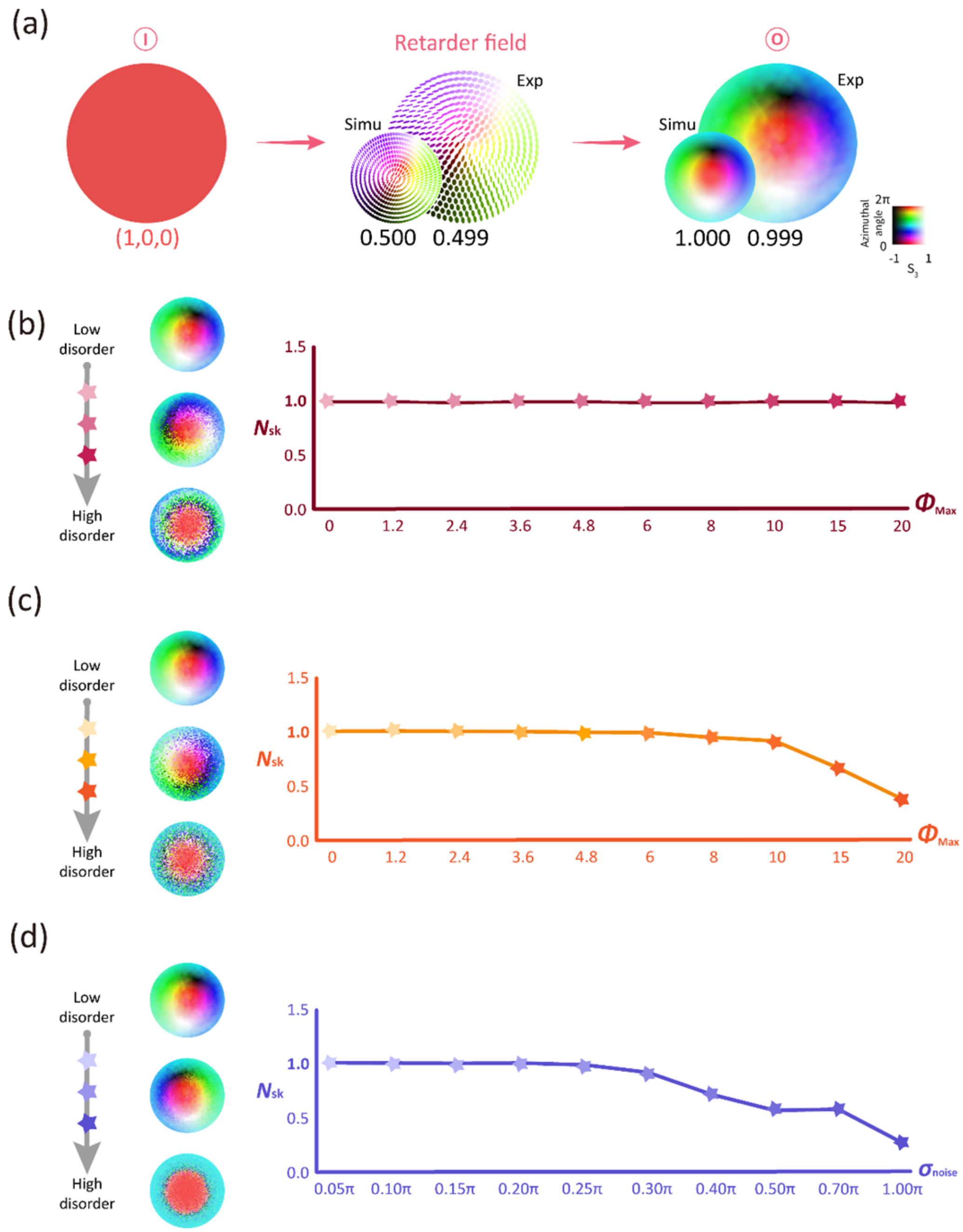

Figure 6: Stability of the topological charge under noise. (a) Reference case: Generation of an $N_{\mathrm{sk}} = 1$ skyrmion from a horizontally polarized incident state. **(b)** Boundary-preserving, spatially correlated disorder. (Left) Representative output Stokes fields at three different noise levels, where a smooth random retardance scrambles the interior while leaving the boundary intact. (Right) Calculated skyrmion number N_{sk} across ten increasing noise levels from 0 to 20 rad. Star markers indicate the noise level, with darker colors representing higher levels of perturbation. **(c)** Boundary-corrupting, spatially correlated disorder. The same smooth random retardance applied uniformly across the entire field, so that the boundary is no longer preserved; panels follow the same layout as (b), with three representative output Stokes fields on the left and the full ten-point N_{sk} curve on the right, again spanning 0 to 20 rad. **(d)** Boundary-corrupting, fully uncorrelated Gaussian disorder. Pixel-wise uncorrelated Gaussian retardance noise applied uniformly across the field; panels follow the same layout as (b), with the noise level set by the standard deviation σ_{noise} ranging from 0 to π.

2. For option (b), we narrowed down the wording of the robustness claims so that they match

what the experiments support. In the Abstract, "high robustness against alignment errors and environmental noise" now reads "robustness against perturbations in practical optical systems". In the Introduction, "accurate charge readout under systematic and random errors (such as complex aberrations and noise)" now reads "accurate charge readout under the aberrations and noise characteristic of practical operation". In the Discussion, the corresponding sentence now reads: "our experiments confirm that the operations remain reliable despite a certain level of practical imperfections inevitably present in our setup, including misalignment between cascaded devices, retardance and angular errors, the temperature dependence of liquid crystal devices and ambient mechanical vibrations, which fall within the regime where topological resilience is maintained."

12. Z. Zhu, M. Janasik, A. Fyffe, D. Hay, Y. Zhou, B. Kantor, T. Winder, R. W. Boyd, G. Leuchs, Z. Shi, Compensation-free high-dimensional free-space optical communication using turbulence-resilient vector beams. Nat. Commun. 12, 1666 (2021).
13. Z. Guo, C. Peters, N. Mata-Cervera, A. N. Vetlugin, R. Guo, P. Zhang, A. Forbes, Y. Shen, Topological robustness of classical and quantum optical skyrmions in atmospheric turbulence. Nat. Commun. 17, 2085 (2026).
14. Y. Ma, Z. Zhao, J. Antonello, M. J. Booth, S. J. Elston, S. M. Morris, C. He, Using optical skyrmions to assess vectorial adaptive optics capabilities in the presence of complex aberrations. Sci. Adv. 11, eadv7904 (2025).
15. C. He, J. Antonello, M. J. Booth, Vectorial adaptive optics. eLight 3, 23 (2023).
16. Y. Zhang, A. A. Wang, R. Zhang, Z. Zhao, Y. Ma, R. Liu, Z.-K. Pong, Y. Cai, C. He, Skyrmions based on optical anisotropy for topological encoding. Light Sci. Appl. 15, 254 (2026).

Comment 3: *The 3 × 3 and 10 × 10 demonstrations are visually compelling, but the evidence for "negligible crosstalk" and "scalable foundation" remains largely qualitative. For an architecture-oriented paper in Nature Communications, representative images and a few selected charge readouts are not sufficient to establish scalability. The authors should provide more quantitative statistics for the multichannel demonstrations. Useful additions would include, for example, channel-by-channel distributions of the measured skyrmion number, mean, and standard deviation across the array; worst-case deviations; a quantitative crosstalk metric; or a yield/accuracy map for all channels (e.g., a 2D deviation heatmap). If such data are already available, the revision may mainly require better presentation; if not, then the current wording could be toned down accordingly.*

Response 3: We thank the reviewer for this comment. The underlying measurements were already collected during our experiments but were not presented in sufficient quantitative detail. We apologize for not including them in the previous version.

In the revised version, we have addressed each of the five quantitative items suggested by the reviewer, and have also moderated the corresponding wording in the main text: (1) Channel-by-channel distributions: the complete per-channel ($N_{\mathrm{sk,simu}}$, $N_{\mathrm{sk,exp}}$) values for every measurement across the 3×3 and 10×10 arrays. (2) Mean and standard deviation across the array. (3) Worst-case deviation. (4) Per-channel relative deviation, $|N_{\mathrm{sk,exp}} - N_{\mathrm{sk,simu}}|/|N_{\mathrm{sk,simu}}|$. (5) Per-channel yield, i.e. whether each channel's deviation falls below a chosen threshold (0.01). The raw values in (1) are provided as Supplementary Data; the statistics in (2), (3), (4) and (5) are summarized in Supplementary Table 1, and the per-channel deviation heatmaps are shown in Supplementary Figure 5. All are described in Supplementary Note 7.

Supplementary Table 1 and Supplementary Figure 5 (see Supplementary Note 7) are reproduced below for the reviewer's convenience:

Quantity	3×3 array	10×10 array
Number of measurements	63	300
Mean $\lvert N_{sk,exp} - N_{sk,simu} \rvert$	0.0009	0.0006
Std $\lvert N_{sk,exp} - N_{sk,simu} \rvert$	0.0007	0.0007
Max $\lvert N_{sk,exp} - N_{sk,simu} \rvert$	0.0030	0.0030
Mean relative deviation (%)	0.181	0.106
Max relative deviation (%)	0.600	0.600
Yield at $\lvert dev \rvert < 0.01$	100%	100%

Supplementary Table 1: Per-channel readout statistics for the 3×3 and 10×10 arrays.

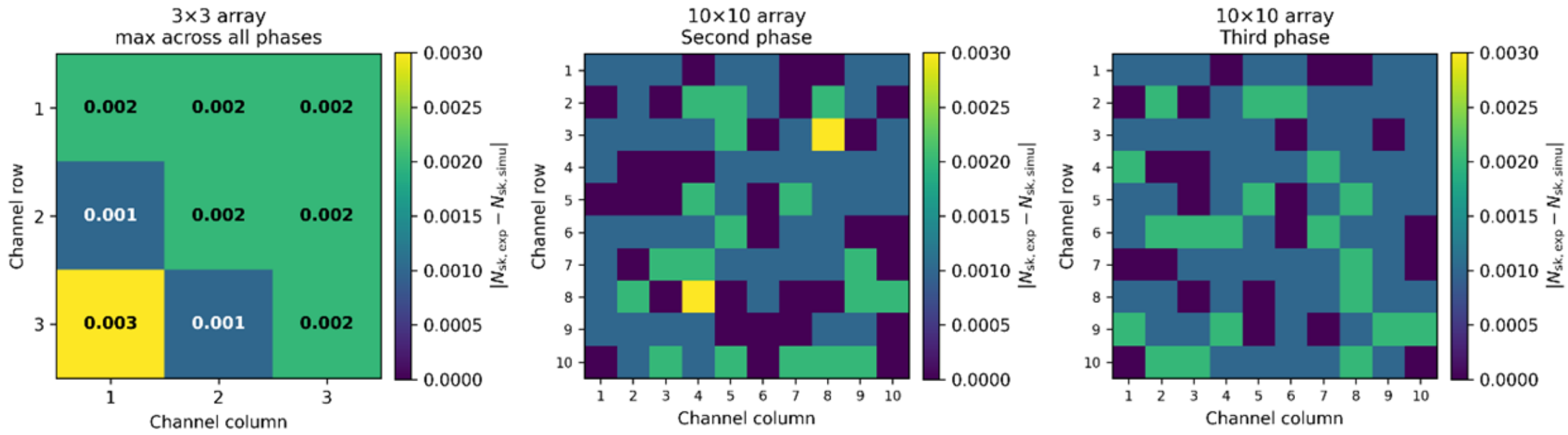

Supplementary Figure 5: Per-channel deviation heatmaps for the multichannel arrays. (a) 3×3 array: maximum $\lvert N_{sk,exp} - N_{sk,simu} \rvert$ at each spatial channel, taken across all measurements. **(b)** 10×10 array, Second phase: per-channel $\lvert N_{sk,exp} - N_{sk,simu} \rvert$. **(c)** 10×10 array, Third phase: per-channel $\lvert N_{sk,exp} - N_{sk,simu} \rvert$.

The complete statistics are now summarized in Supplementary Note 7, which reads:

"Despite an approximately 11-fold increase in channel density between the 3×3 and 10×10 arrays (since the imaged region on the SLM and camera is fixed, each channel's area shrinks by the same 11-fold factor; see Supplementary Note 3), the per-channel statistics remain essentially unchanged: for the 3×3 and 10×10 arrays respectively, the mean deviation $\lvert N_{sk,exp} - N_{sk,simu} \rvert$ is 0.0009 and 0.0006, the standard deviation is identical at 0.0007, the maximum deviation coincides at 0.003, the mean relative deviation is 0.181% and 0.106%, the maximum relative deviation coincides at 0.600%, and the yield at the $\lvert dev \rvert < 0.01$ threshold is 100% in both cases. This consistency is also evident in the 2D deviation heatmaps (Supplementary Fig. 5), which show no pronounced systematic spatial pattern across channels; since crosstalk would produce such a pattern, this indicates that the channel crosstalk in our experiment is not significant. Underlying values are listed in Supplementary Data."

Accordingly, the main text wording has been changed to replace qualitative expressions of crosstalk performance with quantitative statements. The revised passage in the Results section describing the 3×3 array now reads:

"...also serve as quantitative confirmation of independent channel configuration, with the per-channel deviation $\lvert N_{sk,exp} - N_{sk,simu} \rvert$ not exceeding 0.003 across all 9 channels (full statistics

in Supplementary Note 7 and Supplementary Data)."

The revised passage in Methods 3 describing the 10×10 array now reads:

"The experimentally measured Stokes fields confirm that the framework maintains high performance even when scaled to significantly higher channel densities. Since the field fills the same camera region in every array, increasing the array from 3×3 to 10×10 reduces each channel's area by an 11-fold factor; the readout accuracy is nonetheless unchanged, yielding a maximum per-channel $|N_{\text{sk,exp}} - N_{\text{sk,simu}}|$ of 0.003 across all 100 channels, statistically indistinguishable from the 3×3 case (full statistics in Supplementary Note 7 and Supplementary Data)."

Comment 4: *The manuscript repeatedly emphasizes that the primitive library can be cascaded into more complex logic. However, most experimental demonstrations still consist of two-stage operations. For a manuscript whose main claim is architectural, the case would be considerably stronger if the authors could include at least one more nontrivial composed example, such as generation →conversion → register → addition, or an equivalent multistep topological processing chain. Even if a full experimental realization is too demanding for a revision, a rigorous simulated demonstration together with a clear synthesis rule would still strengthen the architecture-level message.*

Response 4: We thank the reviewer for this suggestion. Following the comment, we have added both a synthesis rule and a simulated demonstration to the end of Methods 1. For the demonstration, we simulated a four-stage cascade that runs all four primitives in one chain: a meron generator, a meron register, a meron-to-skyrmion converter, and a skyrmion adder. The skyrmion number evolves as $N_{\text{sk}} = 0.000, 0.500, 0.500, 1.000, 5.000$ (see Fig. 5). The added passage reads:

"The design rules above specify each primitive individually; composing them into a multistep sequence follows directly. Since each primitive acts on the field as a Mueller matrix, a cascade of primitives transforms the input Stokes field as $S_{\text{out}} = M_m \cdots M_2 M_1 S_{\text{in}}$, the ordered product of the per-stage Mueller matrices, and the net change in skyrmion number is the sum of the per-stage contributions fixed by Eq. (6). To show all four primitives working in a single sequence, we simulated a chain combining a meron generator, a meron register, a meron-to-skyrmion converter, and a skyrmion adder (see Fig. 5). Starting from a uniform left-circularly polarized input ($N_{\text{sk}} = 0.000$), the generator produces a meron ($N_{\text{sk}} = 0.500$), the register preserves it ($N_{\text{sk}} = 0.500$), the converter turns it into an order-1 skyrmion ($N_{\text{sk}} = 1.000$), and the adder yields a final order-5 skyrmion ($N_{\text{sk}} = 5.000$), consistent with the per-stage values given by Eq. (6). The skyrmion number evolves through the predicted sequence at every stage."

Fig. 5 is reproduced below for the reviewer's convenience:

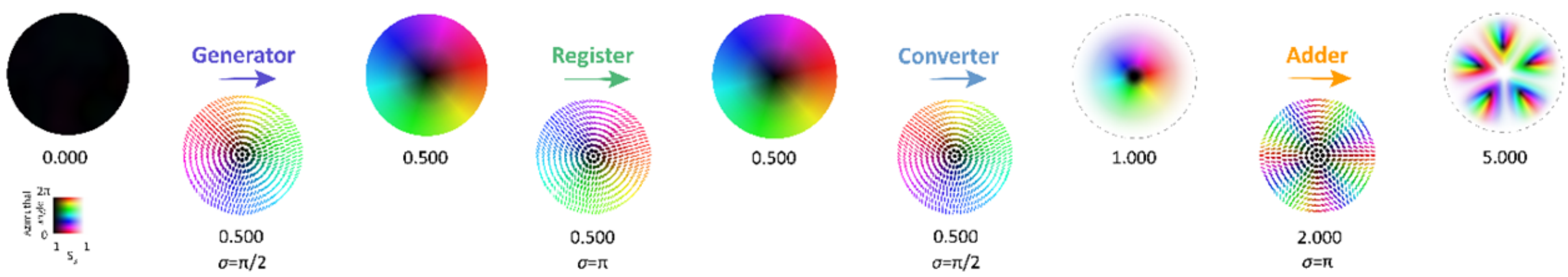

Figure 5: Simulation of a four-stage cascade using the full primitive library. A uniform left-circularly polarized input ($N_{\rm sk} = 0.000$) is processed sequentially through a meron generator ($\sigma = \pi/2$, order 1), a meron register ($\sigma = \pi$, order 1), a meron-to-skyrmion converter ($\sigma = \pi/2$, order 1), and a skyrmion adder ($\sigma = \pi$, order 4), realizing the chain $N_{\rm sk} = 0.000, 0.500, 0.500, 1.000, 5.000$. From left to right, the input field, the fast-axis distributions of the four retarder fields, and the output Stokes field after each stage are shown in sequence. The skyrmion number is given for every field.

Minor Comment 1: *In Method 1, the manuscript states that when σ = π/2, one has ΔNsk = 0.5n, which is consistent with the role of meron generators and skyrmion-to-meron converters. However, the manuscript then appears to state the same result, ΔNsk = 0.5n, also for σ = π, while simultaneously interpreting this case as skyrmion generators and skyrmion-to-skyrmion adders. I suspect this is a typo or a notational issue, but because it sits at the center of the theoretical framework, it must be corrected explicitly. The authors should carefully check the relevant expressions and ensure that all downstream descriptions remain consistent.*

Response (Minor Comment 1): We thank the reviewer for catching this error. The reviewer is correct that the original expression for $\sigma = \pi$ was a typo: the correct value is $\Delta N_{\rm sk} = n$, not $\Delta N_{\rm sk} = n/2$. We apologize for the confusion this caused, and have rewritten Methods 1 to fix the typo and to state the two operating points of the framework clearly. The revised passage now reads:

"The two operating points of the toolkit follow immediately. At $\sigma = \pi/2$, Eq. (6) gives $\Delta N_{\rm sk} = n/2$, identifying the retarder field as a meron generator and as a skyrmion-to-meron interconverter. At $\sigma = \pi$, Eq. (6) gives $\Delta N_{\rm sk} = n$, identifying the same family of retarder fields as a skyrmion generator and as a skyrmion adder."

We have also gone through every downstream usage of these two relations in the main text, the Methods, the Supplementary Information, and the figure captions to ensure full consistency with the corrected expressions.

Minor Comment 2: *The term "register" seems somewhat stronger than the evidence shown. The experiments demonstrate topology-preserving meron transport/relay. If the authors wish to use the word register, they should clarify that this is an architectural abstraction rather than a demonstration of temporal storage in the conventional sense.*

Response (Minor Comment 2): We thank the reviewer for this observation and agree that the original wording could be read as temporal storage in the digital electronics sense, which we did not intend. We have addressed this in two ways.

1. First, at the point where the register primitive is first introduced in the Introduction, we have explicitly stated that the term "register" is used as an architectural abstraction for a topology-preserving meron relay, and not as a temporal storage element in the conventional sense. The revised sentence reads:

"the meron-to-meron register (henceforth the register, used here as an architectural abstraction for a topology-preserving meron relay rather than as a temporal storage element in the conventional sense) is devoted exclusively to transporting merons without changing their boundary states and topological charges."

2. Second, where the four operations of the framework are listed at the function level, we have replaced "storage" with "transport" to align with the reviewer's accurate terminology ("topology-preserving meron transport/relay") and to avoid any unintended suggestion of temporal latching. The revised sentence in the Discussion now reads:

"In this work, we present and experimentally validate a platform-independent operational framework for manipulating the topological charge of polarization textures, enabling skyrmion-based optical processing encompassing generation, conversion, transport, and arithmetic."

Minor Comment 3: *Likewise, the phrase "programmable logic array" may overstate the present scope. The paper demonstrates topological state generation, conversion, transport, and arithmetic, but not Boolean completeness, fan-in/fan-out, or a full digital logic abstraction. The terminology should be calibrated accordingly.*

Response (Minor Comment 3): We thank the reviewer for this observation and agree that the term "programmable logic array" may cause unexpected confusion. To address this, we have changed the terminology throughout the manuscript and have added an explicit statement of scope in the Discussion.

1. The title of the manuscript has been changed from "Topologically robust programmable logic arrays using light and matter skyrmions" to "Topologically robust programmable processing arrays using light and matter skyrmions". The word "processing" accurately encompasses the four operations demonstrated in this study, namely generation, conversion, transport and arithmetic.

2. Following the same principle, we have replaced "logic" with "topological processing", "processing", "cascaded operations" or "operation-level reconfigurability" in eleven further locations across the Abstract, the Introduction, the Results, the figure captions and the Discussion, in each case selecting the replacement that most accurately reflects the operation being described.

3. Finally, we have added a fourth point to the extensions paragraph of the Discussion that frames Boolean completeness and fan-in/fan-out routing as a direction for future work:

"Fourth, a promising direction is to align this optical framework with the full digital logic abstraction of conventional electronics, such as Boolean operations and fan-in / fan-out routing. One route to multi-input fan-in and multi-output fan-out is to introduce a diffractive element into the optical path to couple otherwise independent channels [10][17]; bridging toward Boolean logic [18-20] is also a further step along this direction. Together these point to a wide space for future topological processing architectures."

17. X. Lin, Y. Rivenson, N. T. Yardimci, M. Veli, Y. Luo, M. Jarrahi, A. Ozcan, All-optical machine learning using diffractive deep neural networks. Science 361, 1004-1008 (2018).
18. X. Zhang, M. Ezawa, Y. Zhou, Magnetic skyrmion logic gates: conversion, duplication and merging of skyrmions. Sci. Rep. 5, 9400 (2015).
19. N. Sisodia, J. Pelloux-Prayer, L. D. Buda-Prejbeanu, L. Anghel, G. Gaudin, O. Boulle, Robust and programmable logic-in-memory devices exploiting skyrmion confinement and channeling using local energy barriers. Phys. Rev. Appl. 18, 014025 (2022).
20. D. Yu, H. Yang, M. Chshiev, A. Fert, Skyrmions-based logic gates in one single nanotrack completely reconstructed via

chirality barrier. Natl. Sci. Rev. 9, nwac021 (2022).

Minor Comment 4: *Since the paper relies heavily on the distinction between skyrmions and merons for function allocation, the Discussion would benefit from a clearer explanation of why this separation is advantageous beyond the pixel-resolution argument already mentioned.*

Response (Minor Comment 4): We thank the reviewer for this observation. We have added a paragraph to the Discussion, explaining the advantages of the skyrmion–meron role separation beyond the pixel-resolution argument. The new paragraph reads:

"This framework builds on a natural division of labor between skyrmions and merons. Beyond the information-density argument introduced in the Results, the skyrmion–meron role separation provides further advantages. The main advantage is reuse through unification: the generator, converter and adder share a common pattern, a meron fast-axis distribution derived in Methods 1, differing only in the scalar retardance δ. A meron register naturally keeps this shared pattern, whereas a skyrmion register would require a skyrmion fast-axis distribution that breaks it. Thus, using a meron for transport keeps all four primitives in one matter-design family and enables field reuse. Furthermore, because the primitives differ only in δ, a tunable-retardance device [21] could in principle collapse the whole library onto a single element. We also note that a further advantage arises in confined-mode platforms such as waveguides, where the natural propagating state is often a meron rather than a skyrmion [22]. Treating merons as equal carriers to skyrmions thus matches what these platforms support."

21. A. M. Shaltout, V. M. Shalaev, M. L. Brongersma, Spatiotemporal light control with active metasurfaces. Science 364, eaat3100 (2019).
22. A. A. Wang, Y. Ma, Y. Cai, J. Qin, B. Dong, C. He, Optical skyrmions in waveguides. arXiv:2505.06735 (2025).

Minor Comment 5: *The discussion regarding the transferability of the framework to diverse technologies appears overly simplified. While topological robustness relaxes certain fabrication errors, the authors should provide a brief qualitative analysis of the unique physical constraints inherent to each specific technological implementation, and concretely discuss the feasibility of such extensions.*

Response (Minor Comment 5): We thank the reviewer for this comment. We have added a dedicated Supplementary section, "Implementation on alternative platforms," which addresses each technology in turn. It reads:

"The retarder fields in this work are realized with an LC-SLM cascade, but several mature technologies can likewise pattern spatially varying birefringence, each subject to its own physical constraints. The noise-robustness experiments in Methods 2 relax part of these constraints for our architecture. First, the operation of each primitive is governed by the retarder field at its boundary, making it insensitive to interior imperfections. Second, the topological charge remains reliable even when the boundary itself is imperfect, provided the deviation stays within the tolerance quantified in Methods 2. Based on these two results, reproducing a primitive on another platform mainly requires the boundary to be fabricated within this tolerance, with the accuracy requirement on the interior correspondingly relaxed. We discuss three representative platforms below.

We first briefly introduce the three technologies. 1. Liquid crystal polymers form one route: a reactive mesogen is aligned into a designed director pattern, usually by photoalignment, and then fixed by polymerization into a permanent solid film that imparts a geometric phase, reaching very high efficiency with continuous phase profiles [23]. 2. Femtosecond-laser-written birefringent glasses are another: self-assembled nanogratings produce a permanent and stackable form birefringence, with the fast axis set by the writing polarization [24]. 3. Dielectric metasurfaces are a third: subwavelength structures control the phase and polarization of light, giving elements that can be made very thin [27].

We then discuss the physical constraints of each platform, and how our framework relaxes them. 1. For liquid crystal polymers, the geometric phase is set point by point by the local director orientation, so the encoded function requires director accuracy across the whole element; alignment defects tend to appear where the pattern varies rapidly or the anchoring is weak [23]. To suppress these defects, high-resolution photoalignment techniques have been developed, at the cost of more complex exposure systems and processing [23]. For our primitives, we mainly care about the director near the boundary, so the fabrication does not heavily rely on high-resolution photoalignment: defects in the interior matter much less, and the yield improves at a lower cost. 2. For femtosecond-laser-written birefringent glasses, the written retardance and fast axis drift across the device, driven by depth-dependent spherical aberration during writing [25]. To compensate for this aberration, adaptive wavefront correction has been introduced into the writing system, at the cost of extra hardware and calibration [26]. For our primitives, what matters most is the state of the boundary, so the writing does not depend strongly on adaptive wavefront correction: the drift in the interior is much less of an issue, and the fabrication becomes easier and cheaper. 3. For dielectric metasurfaces, nanometer-scale geometry errors in each meta-atom translate directly into phase errors, so the entire device must be fabricated to nanometer tolerances [27]. To relax this demand, fabrication-tolerant design strategies have been developed, at the cost of extra design effort and some loss of peak performance [28]. For our primitives, the requirement falls mainly on the meta-atoms around the boundary, so there is much less need for fabrication-tolerant design strategies: the rest of the device can be made with relaxed precision, which lowers the difficulty and cost of fabrication.

Taken together, these discussions indicate that the framework can, in most cases, be implemented on these platforms with a reduced fabrication demand."

The Discussion also points to this section:

"Beyond reliable operation, this robustness also lowers the fabrication demands of each platform, such as liquid crystal polymers [23][29], femtosecond-laser-written birefringent glasses [24][30], and dielectric metasurfaces [27], making the framework easier to implement across different technologies (see Supplementary Note 8). Such platform flexibility means the approach can move toward more compact, integrated photonic processing circuits."

23. M. Jiang, Y. Guo, H. Yu, Z. Zhou, T. Turiv, O. D. Lavrentovich, Q.-H. Wei, Low f-number diffraction-limited Pancharatnam-Berry microlenses enabled by plasmonic photopatterning of liquid crystal polymers. Adv. Mater. 31, 1808028 (2019).
24. M. Beresna, M. Gecevičius, P. G. Kazansky, T. Gertus, Radially polarized optical vortex converter created by femtosecond laser nanostructuring of glass. Appl. Phys. Lett. 98, 201101 (2011).
25. J. Lu, J. Tian, B. Poumellec, E. Garcia-Caurel, R. Ossikovski, X. Zeng, M. Lancry, Tailoring chiral optical properties by femtosecond laser direct writing in silica. Light Sci. Appl. 12, 46 (2023).

26. N. Bisch, J. Guan, M. J. Booth, P. S. Salter, Adaptive optics aberration correction for deep direct laser written waveguides in the heating regime. Appl. Phys. A 125, 364 (2019).
27. H.-T. Chen, A. J. Taylor, N. Yu, A review of metasurfaces: physics and applications. Rep. Prog. Phys. 79, 076401 (2016).
28. E. W. Wang, D. Sell, T. Phan, J. A. Fan, Robust design of topology-optimized metasurfaces. Opt. Mater. Express 9, 469-482 (2019).
29. C. C. Tartan, P. S. Salter, T. D. Wilkinson, M. J. Booth, S. M. Morris, S. J. Elston, Generation of 3-dimensional polymer structures in liquid crystalline devices using direct laser writing. RSC Adv. 7, 507-511 (2017).
30. J. Allison, et al., Laser writing in glass for dense, fast and efficient archival data storage. Nature 650, 606-612 (2026).

We thank the reviewer once again for all of these valuable and constructive comments, and we hope that the revised manuscript now meets with the reviewer's approval.

Reply to Reviewer #3

In the manuscript titled "Topologically robust programmable logic arrays using light and matter skyrmions" by Liu et al., the authors propose a scheme for implementing arithmetic operations using optical skyrmions and merons. Building upon their previous research, the study utilises retarders with engineered spatial profiles as key components for the generation and conversion of skyrmionic fields. A notable advancement from earlier work is the distinct roles assigned to skyrmions and merons; specifically, the conversion of skyrmions into merons and the preservation of meron states when operations are not required. Furthermore, the authors demonstrate that multiple channels can be integrated into a single optical element for parallel transmission.

I find that this work has the potential to broaden the applications of optical skyrmions and offer new directions for optical information processing. The presented data are technically sound, and the manuscript is well-organised overall. However, before recommending publication in Nature Communications, I would like the authors to address the specific concerns outlined below.

We sincerely thank the reviewer for their careful reading of our manuscript and for their positive evaluation of our work. We are particularly grateful to the reviewer for noting that "this work has the potential to broaden the applications of optical skyrmions and offer new directions for optical information processing". We also appreciate the reviewer's recognition of the skyrmion–meron role separation and the multichannel integration as notable advances.

We apologize that the original version of the manuscript did not address several specific points with sufficient clarity, and have carefully revised the manuscript and the Supplementary Information to address each of the reviewer's concerns. Please allow us to provide a point-by-point response below.

Comment 1: *Whilst the authors claim that the present approach increases design flexibility, I remain somewhat unconvinced regarding the necessity of the additional complexity involved in converting skyrmions into merons. Could the authors clarify whether a retardance profile could instead be implemented to preserve the skyrmion number whilst remaining in the skyrmion mode?*

Response 1: We thank the reviewer for this question. The reviewer is right that a designed retardance profile can preserve the skyrmion number while staying in the skyrmion mode, which would be a skyrmion-mode register. Such a register is feasible on its own; the reason we instead route the transport role through merons is architectural, not a matter of single-primitive feasibility.

To make this explicit, we have added a paragraph to the Discussion setting out the advantages and necessity of the skyrmion–meron role separation:

"This framework builds on a natural division of labor between skyrmions and merons. Beyond

the information-density argument introduced in the Results, the skyrmion–meron role separation provides further advantages. The main advantage is reuse through unification: the generator, converter and adder share a common pattern, a meron fast-axis distribution derived in Methods 1, differing only in the scalar retardance δ. A meron register naturally keeps this shared pattern, whereas a skyrmion register would require a skyrmion fast-axis distribution that breaks it. Thus, using a meron for transport keeps all four primitives in one matter-design family and enables field reuse. Furthermore, because the primitives differ only in δ, a tunable-retardance device [1] could in principle collapse the whole library onto a single element. We also note that a further advantage arises in confined-mode platforms such as waveguides, where the natural propagating state is often a meron rather than a skyrmion [2]. Treating merons as equal carriers to skyrmions thus matches what these platforms support."

Beyond this architectural role, the introduction of merons also brings the information-density advantage noted in the Results. The relevant passage reads:

"increasing N_{sk}, whether integer or fractional, leads to increasing complexity of the underlying polarization field. This, in turn, results in practical engineering limits due to limited resolution of the underlying matter field arising from finite pixel size. Adopting merons and skyrmions together thus helps overcome these real-world issues. For any given maximum topological charge, three times as many distinct units become available. The same operation can then be realized with only a third of the maximum charge, which reduces the overall system complexity. Naturally, other fractional units could be employed to further increase the number of computational units, and we leave a detailed investigation of their design as a possible extension of this work."

Concretely, the factor of three arises because merons add half-integer units between the integer skyrmion states: up to a maximum charge of one, a skyrmion-only scheme offers a single unit ($N_{\mathrm{sk}} = 1$), whereas adding merons gives three (an order-1 meron at $1/2$, an order-1 skyrmion at 1, and an order-2 meron at 1), tripling the number of distinct units available at the same maximum charge. The framework thus retains the possibility of finer fractional subdivision; our adoption of the meron here is the joint outcome of the considerations above, including its compatibility with waveguides [2], rather than being dictated by any single factor.

1. A. M. Shaltout, V. M. Shalaev, M. L. Brongersma, Spatiotemporal light control with active metasurfaces. Science 364, eaat3100 (2019).
2. A. A. Wang, Y. Ma, Y. Cai, J. Qin, B. Dong, C. He, Optical skyrmions in waveguides. arXiv:2505.06735 (2025).

Comment 2: *Whilst integrating multiple channels onto a single optical element may be useful, the current methodology is essentially synonymous with employing multiple separate SLMs, and the results are therefore predictable. For instance, if the integration density were to be increased, the area available to each channel would necessarily decrease; to what extent would this be acceptable? At first glance, one would expect crosstalk with adjacent channels to increase significantly. Furthermore, as beam divergence would become more pronounced, would this not complicate the configuration of the 4f system, as illustrated in the supplementary material?*

Response 2: We thank the reviewer for these questions, which prompted us to make the per-channel-area scaling of our data explicit and to state directly what sets the density limit. We

address the premise first, then channel area, crosstalk, and the 4f system in turn.

1. *On the premise*. The hardware is indeed a cascade of SLMs, but our contribution lies not in the hardware but in platform-independent architecture. The SLM cascade is simply a reconfigurable, high-degree-of-freedom platform for validating this architecture, which let us realize all four primitives and run the 3×3 and 10×10 arrays on one setup. The whole array follows from a single retarder-field design family, in which all four primitives share the order-n meron fast-axis distribution derived in Methods 1. Because the operations share one fast-axis pattern, this single matter design can be fixed into one processing layer, with the operation of each channel set by its incident state (see Fig. 4) rather than by a separately engineered device per channel. That the behavior is predictable is, to us, a strength rather than a limitation: it follows from explicit design rules, and being platform-independent, this rule-governed framework scales in density and can later be moved to the compact platforms.

2. *On channel area*. To the reviewer's question of how far the per-channel area can shrink: in our data the topological charge is still read out correctly down to 25×25 SLM pixels per channel, the smallest we have verified. As stated in Methods 3: “The accurate recovery of a topological invariant places only gentle demands on spatial sampling [3]: as shown in Supplementary Note 6, the topological charge is still recovered correctly down to 25×25 SLM pixels (corresponding to about 169×169 camera pixels)”. We note this figure is not a fundamental limit but simply the smallest we demonstrated here, corresponding to a physical channel size of about $313 \times 313\ \mu m^2$ given the $12.5\ \mu m$ SLM pixel pitch (see Supplementary Note 3). In fact, preliminary results from our group show that the same skyrmion textures can be generated on a dielectric metasurface with a device diameter of around $100\ \mu m$, roughly three times smaller in linear size, suggesting that the per-channel footprint can be pushed substantially further. A systematic study of this miniaturized platform is beyond the scope of the present work and is left for future work.

3. *On crosstalk*. Increasing the array from 3×3 to 10×10 is itself a direct test, since it reduces the per-channel area to the smallest we demonstrate in the main text. As emphasized in Methods 3, “since the field fills the same camera region in every array, increasing the array from 3×3 to 10×10 reduces each channel's area by an 11-fold factor”, and Supplementary Note 3 shows the sizes of both arrays: “each channel occupies approximately 933×933 camera pixels (138×138 SLM pixels) in the 3×3 array and 280×280 camera pixels (41×41 SLM pixels) in the 10×10 array”. To quantify the crosstalk, we added a dedicated section, Supplementary Note 7 (including Supplementary Table 1 and Supplementary Figure 5).

Supplementary Figure 5 is reproduced below for the reviewer's convenience:

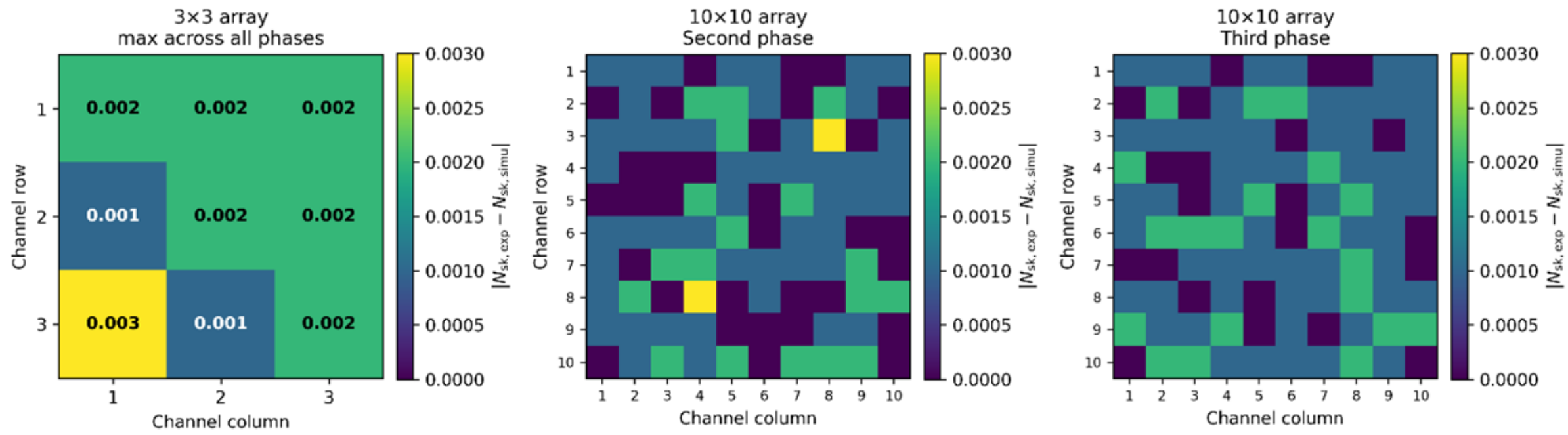

Supplementary Figure 5: Per-channel deviation heatmaps for the multichannel arrays. (a) 3×3 array: maximum $|N_{\mathrm{sk,exp}} - N_{\mathrm{sk,simu}}|$ at each spatial channel, taken across all measurements. **(b)** 10×10 array, Second phase: per-channel $|N_{\mathrm{sk,exp}} - N_{\mathrm{sk,simu}}|$. **(c)** 10×10 array, Third phase: per-channel $|N_{\mathrm{sk,exp}} - N_{\mathrm{sk,simu}}|$.

As reported in Supplementary Note 7: "Supplementary Figure 5 shows no pronounced systematic spatial pattern across channels; since crosstalk would produce such a pattern, this indicates that the channel crosstalk in our experiment is not significant". The expected growth of crosstalk with density is therefore not seen even at the smallest channel area we reach. This robustness follows from the topological protection of the field, which makes the readout inherently insensitive to crosstalk.

4. *On the 4f system*. In our setup, the beam divergence does not grow with the channel density. As described in Supplementary Note 3: "Successive SLMs are connected by unit-magnification (1:1) 4f relay systems, which image each SLM plane onto the next and keep the field in an imaging configuration throughout the cascade." Each relay images the entire SLM plane, of which the channels are subregions defined simply by the displayed pattern rather than by physical apertures, so the imaged aperture stays the size of the full plane and adding channels only subdivides it more finely, without changing the 4f configuration, and divergence stays suppressed along the cascade, since the field remains in an imaging configuration rather than propagating freely. Increasing the density therefore does not complicate the 4f system.

3. A. A. Wang, Z. Zhao, Y. Ma, Y. Cai, R. Zhang, X. Shang, Y. Zhang, J. Qin, Z.-K. Pong, T. Marozsák, B. Chen, H. He, L. Luo, M. J. Booth, S. J. Elston, S. M. Morris, C. He, Topological protection of optical skyrmions through complex media. Light Sci. Appl. 13, 314 (2024).

Comment 3: *Following on from my previous point, the current setup necessitates a 4f configuration for each retarder. I suspect that the overall system size is constrained by the focal lengths of the lenses, which may hinder the achievement of a compact design. Would it not be possible to cascade the retarders without intervening lenses, similar to passing the beam through an HWP and QWP in direct sequence? Whilst I anticipate that the results would be distorted due to diffraction, I should like the authors to clarify whether such effects might be permissible.*

Response 3: We thank the reviewer for raising both the question of system size and the possibility of a lens-free cascade, and we address them in turn.

1. *On system size*: yes, as the reviewer has mentioned, the lenses make the present setup not compact. Its size is set by the focal lengths of the 4f relays between successive modulators. This

is by design, as the setup is a high-precision, fully programmable platform for validating the architecture and its primitives, for which the reconfigurability of the LC-SLMs is suited. A compact realization could come not from this bulk-optical setup but from the static devices discussed in Supplementary Note 8. These more compact technologies are also noted in the Discussion: "...such as liquid crystal polymers [4][5], femtosecond-laser-written birefringent glasses [6][7], and dielectric metasurfaces [8] ... Such platform flexibility means the approach can move toward more compact, integrated photonic processing circuits." How the 4f relays are then handled depends on the platform: femtosecond-laser-written birefringent glass can in principle be stacked along the propagation direction within a single substrate, largely removing the need for intermediate imaging [6][7], whereas discrete planar devices such as metasurfaces [9][10] or liquid crystal polymers [11][12] generally still require relaying between elements, which connects to the second question.

2. *On removing the lenses*: in the current setup we keep the lenses; whether they can be removed depends on the propagation regime. Without the imaging relays, the field propagates freely between elements and is subject to diffraction, as the reviewer has mentioned. We have added this to Supplementary Note 3:

"removing these 4f relays so that the field propagates freely between elements would reduce the system size but reintroduce propagation: the skyrmion number is preserved under certain free-space propagation in the paraxial, boundary-preserving regime [3], whereas outside this regime the charge can undergo controlled number transitions [13]. Whether a lens-free cascade stays within the protected regime is therefore set by how the structured field propagates, and is left to future work."

In the Discussion, we now flag this as a future direction:

"First, while the present work operates in free space and keeps each layer in an imaging configuration via 4f relays, moving toward more integrated implementations requires relaying the field between elements through free-space propagation or confined media such as waveguides [2], where preserving the topological charge becomes the key question. Addressing this needs engineering the inter-element relay and/or developing advanced topological frameworks [14] that remain robust under propagation, making the preservation of topological charge an important extension of this work."

4. M. Jiang, Y. Guo, H. Yu, Z. Zhou, T. Turiv, O. D. Lavrentovich, Q.-H. Wei, Low f-number diffraction-limited Pancharatnam-Berry microlenses enabled by plasmonic photopatterning of liquid crystal polymers. Adv. Mater. 31, 1808028 (2019).
5. C. C. Tartan, P. S. Salter, T. D. Wilkinson, M. J. Booth, S. M. Morris, S. J. Elston, Generation of 3-dimensional polymer structures in liquid crystalline devices using direct laser writing. RSC Adv. 7, 507-511 (2017).
6. M. Beresna, M. Gecevičius, P. G. Kazansky, T. Gertus, Radially polarized optical vortex converter created by femtosecond laser nanostructuring of glass. Appl. Phys. Lett. 98, 201101 (2011).
7. J. Allison, et al., Laser writing in glass for dense, fast and efficient archival data storage. Nature 650, 606-612 (2026).
8. H.-T. Chen, A. J. Taylor, N. Yu, A review of metasurfaces: physics and applications. Rep. Prog. Phys. 79, 076401 (2016).
9. R. C. Devlin, M. Khorasaninejad, W. T. Chen, J. Oh, F. Capasso, Broadband high-efficiency dielectric metasurfaces for the visible spectrum. Proc. Natl. Acad. Sci. U.S.A. 113, 10473-10478 (2016).
10. N. Yu, P. Genevet, M. A. Kats, F. Aieta, J.-P. Tetienne, F. Capasso, Z. Gaburro, Light propagation with phase discontinuities: generalized laws of reflection and refraction. Science 334, 333-337 (2011).
11. H. Zeng, P. Wasylczyk, C. Parmeggiani, D. Martella, M. Burresi, D. S. Wiersma, High-resolution 3D direct laser writing for liquid-crystalline elastomer microstructures. Adv. Mater. 26, 2319-2322 (2014).
12. C. C. Tartan, P. S. Salter, M. J. Booth, S. M. Morris, S. J. Elston, Read on demand images in laser-written polymerizable liquid crystal devices. Adv. Opt. Mater. 6, 1800515 (2018).

13. X. Fan, X. Wu, K. Ren, L. Zhou, X. Guo, B. Wei, Y. Zhang, S. Liu, P. Li, J. Zhao, Topological state and number transitions of optical skyrmions upon free-space beam propagation. Commun. Phys. 8, 500 (2025).
14. A. A. Wang et al., Generalized skyrmions. arXiv:2409.17390 (2024).

Comment 4: *Throughout the manuscript, simulation results are superimposed onto the experimental data, thereby obscuring portions of the primary results. I consider this practice inappropriate as it compromises the verifiability of the experimental findings. I request that the experimental results be presented in a manner that ensures they are fully visible. For instance, the experimental result for the adder in Fig. 3b (specifically the 2.995 case) appears to be white (S_3 ~ 1) just before it is obscured by the simulation overlay; this suggests a distribution that may not align with the simulation. However, a thorough verification is currently impossible because the majority of the skyrmion on the left remains hidden.*

Comment 5: *Following on from my previous point, do these plot ranges represent the integration domains used for calculating the skyrmion number? For instance, the converter results in Fig. 3b yield a remarkably ideal skyrmion number of 0.999; however, S_3 clearly does not appear to reach 1 at the boundaries. Whilst this may be an issue with the colour function, I should like to know the precise value of S_3 at the boundary. Furthermore, does the measurement system possess sufficient significant figures to justify a result of 0.999? Additionally, could the authors clarify how many pixels a single skyrmion spans in their experimental data?*

Response 4 and 5: We thank the reviewer for these two comments, which we address together as both concern the presentation of Fig. 3 and the quantitative reliability of the reported skyrmion numbers. We have revised both the presentation and the supporting analysis as detailed below.

1. *Presentation and visibility (Comment 4)*. We have re-plotted Fig. 3 so that the experimental and simulated fields are shown separately, with the experimental results presented in full and no longer obscured by any overlay. The same change has been applied to the corresponding figures throughout the manuscript. The revised Fig. 3 is reproduced below.

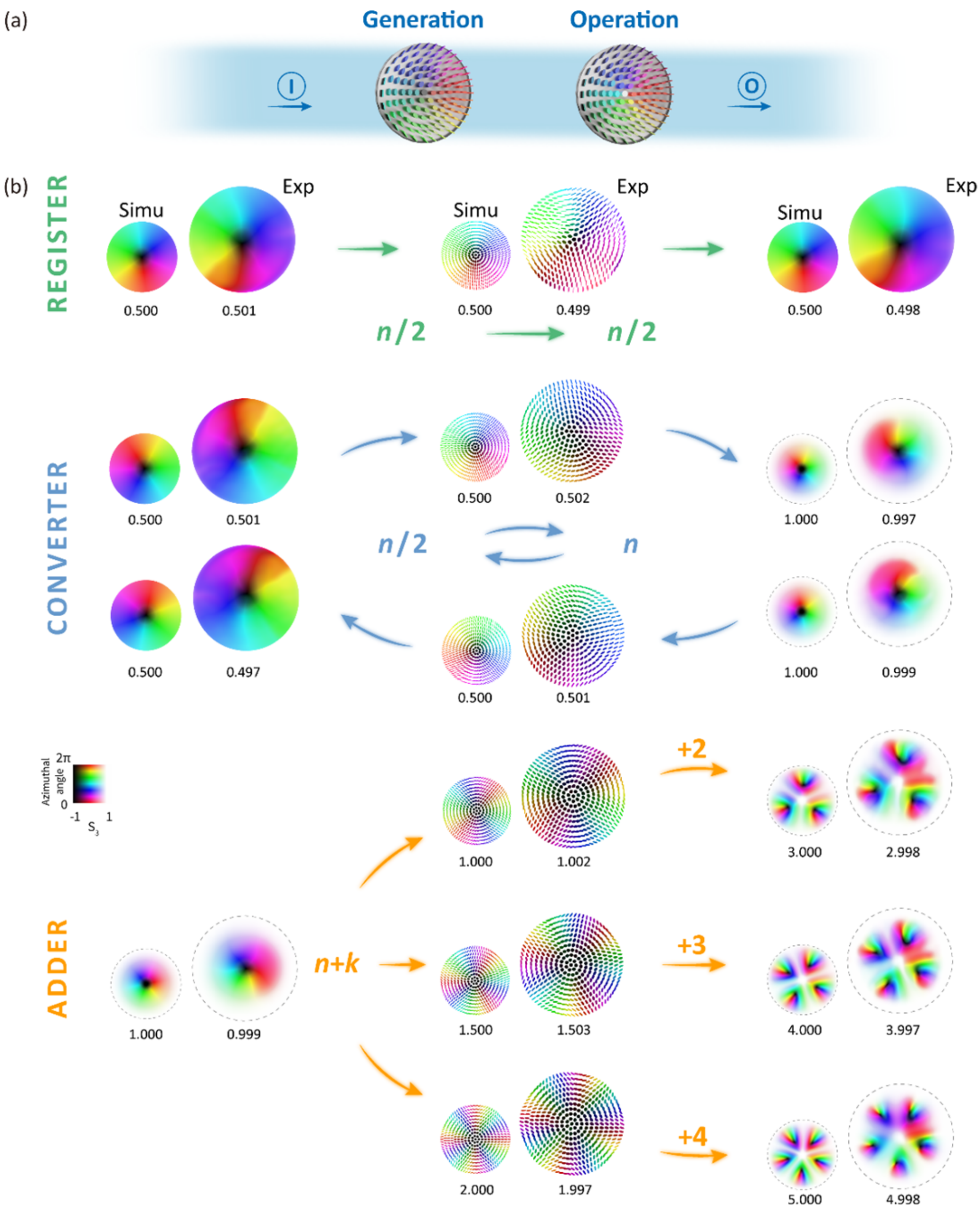

2. *The apparent white region in the adder case (Comment 4)*. Following the reviewer's comment, we clarify that the white region is in fact correct rather than a sign of a mismatch; in addition, the boundary is expected to be white and remains unchanged throughout the operation. The reviewer refers to a region in the interior of the experimental field, close to the part obscured by the overlay, that appears white while the simulation at the same location does not, suggesting that the two might not align. This region corresponds to a location where S_3 approaches $+1$, which maps to white in our color scheme, and is therefore exactly as expected: both the experiment and the simulation should be white there. In the original color function the white region corresponding to S_3 approaching $+1$ was not clearly visible, so that states with S_3 near the poles appeared with low contrast. We have adopted an adjusted color function that instead shows states with S_3 approaching $+1$ clearly in both the simulated and the experimental fields, and we now include the corresponding color bar in the figure, together with a gray dashed circle on each skyrmion result to mark the integration-domain boundary.

3. *Re-acquisition of the data (Comment 4 and 5).* Beyond adjusting the color function, we re-acquired the experiments for the cases concerned using the new color function, and the experimental results in Fig. 3 have been updated accordingly. The original experimental data remain valid. To make this explicit, we provide below a dedicated comparison figure for the reviewer, showing the old and new color bars together with the old and revised simulations and the old and new experimental results side by side for representative skyrmion and adder cases, including the 2.995 case. The white region becomes clearly visible under the adjusted color function in every case.

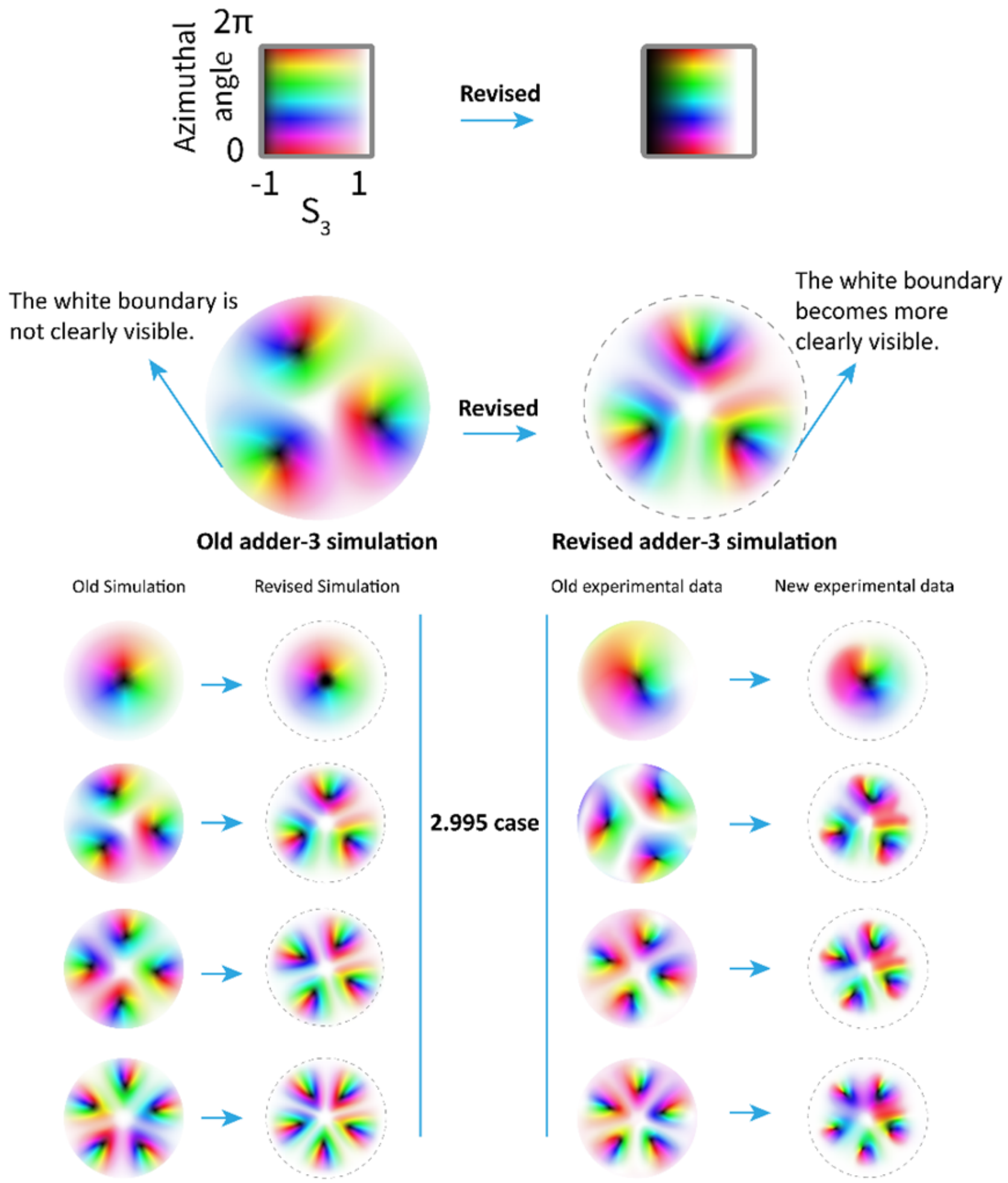

Comparison Figure: Comparison of the original and adjusted color functions, each shown with its color bar, simulations, and experimental results. Under the adjusted color function the white regions are clearly visible.

4. *Texture deviation versus topological charge (Comment 4).* This also addresses the reviewer's underlying concern directly, since the comparison being drawn is between the textures of experiment and simulation. Close agreement of the full Stokes texture is of course desirable and reflects a high-quality measurement. However, the quantity that carries the information, the skyrmion number, is mainly determined by the boundary of the field and not by pointwise agreement of the interior texture, so that local deviations in the interior between measurement and simulation rarely affect the measured skyrmion number [3]. This is the topological protection that the present framework relies on, and it is why the skyrmion number, rather than

the detailed texture, is the right quantity to compare. We have made this point explicit in the main text, adding to the Results that "this charge is mainly determined by the boundary of the field rather than by the pointwise interior texture, so that local deviations in the interior texture between measurement and simulation rarely affect the measured skyrmion number, consistent with the topological protection established in Methods 1 and 2."

5. *Plot range and integration domain (Comment 5).* Yes. The plotted region corresponds to the integration domain used to compute the skyrmion number, and in the revised skyrmion results this domain is now marked explicitly by the gray dashed circle described above.

6. *Precise value of S_3 at the boundary (Comment 5).* We evaluated the mean S_3 around the integration-domain boundary for the converter skyrmion fields in Fig. 3b and obtained 0.99986 and 0.99913 for the lower and upper fields respectively. The boundary is therefore at S_3 approaching $+1$ to within about 10^{-3}, consistent with the white boundary expected for these states. The reviewer's intuition that this might be a color-function issue was exactly right: the earlier impression that S_3 did not reach unity at the boundary was the color-compression artifact described above and not a property of the field.

7. *Significant figures and the value 0.999 (Comment 5).* The skyrmion number is obtained by numerically integrating the Stokes field over the full skyrmion, which is sampled by of order 10^6 camera pixels (see Supplementary Note 3) in the main-text experiments and by about 2.8×10^4 pixels even at the reduced 169 by 169 sampling verified in Supplementary Note 6, so the numerical precision of the integral is well below 10^{-3} and the third decimal figure is meaningful. We further note that the reported figures are conservative: as with the boundary S_3 values above, measured values typically fall within about 10^{-3} of the ideal integer, and a value of, for example, 0.99993 is reported as 0.999 rather than rounded to an idealized 1.000, so as to reflect the true experimental deviation rather than overstate the accuracy.

8. *Pixels spanned by a single skyrmion (Comment 5).* In the experiments reported in the main text, each skyrmion is imaged to fill a region of approximately 2800 by 2800 camera pixels, corresponding to about 414 by 414 SLM pixels under the unit-magnification relay. We have further verified in Supplementary Note 6 that the topological charge is recovered correctly down to 25 by 25 SLM pixels per channel (approximately 169 by 169 camera pixels). The full optical layout and component specifications, including these pixel counts, are now given in Supplementary Note 3.

Comment 6: *Finally, purely out of curiosity, I wonder whether it would be possible to transfer or mix data between channels, rather than simply processing them in parallel. For instance, could this be achieved by inserting a diffractive element or similar component into the optical path? If such inter-channel interaction were feasible, it would allow for more sophisticated calculations, which in my view would further enhance the value and impact of the proposed scheme.*

Response 6: We thank the reviewer for this stimulating suggestion, which points to a valuable direction for extending the scheme. Indeed, our recent work has already taken a first step in this direction [15], as mentioned later in this response.

1. We would first note that the independence of the channels in the present work is a deliberate

design choice rather than a limitation: it is what allows the array to perform many operations in parallel with negligible crosstalk, as quantified in our responses to Comment 2. Introducing controlled interaction between channels, as the reviewer suggests, is a natural extension of the same framework rather than something it precludes.

2. For the diffractive element, we agree that such an element placed in the optical path couples spatially separate channels through diffraction, so that multiple channels can be brought into a joint computation in which the outputs depend on several inputs at once. This is precisely the operating principle of diffractive optical networks, in which stacked passive diffractive layers couple many spatial points into a collective, all-optical computation [16]. Such diffractive processing has already been applied to skyrmionic fields, where a diffractive network was used to read out skyrmionic topologies of light [17].

3. In the language of our framework, such coupling would correspond to multi-input, multi-output topological operations, in which the topological charges carried by two or more channels are combined or redistributed across multiple outputs, extending the single-input, single-output primitives demonstrated here to multiple inputs and outputs. We have added this route directly to the Discussion, where a diffractive element is now named as a way to support fan-in and fan-out: "One route to multi-input fan-in and multi-output fan-out is to introduce a diffractive element into the optical path to couple otherwise independent channels [16][17]". The inter-channel interaction the reviewer envisages can be supported by this physical route. A concrete step in this direction is our recent work [15], where a single passive retarder–diattenuator–retarder cascade is designed to satisfy three arbitrary input–output relations at once, so that one element realizes three distinct conversions and supports both time-division and wavelength-division multiplexing.

15. R. Zhang, T. Marozsák, A. A. Wang, Y. Zhang, Y. Ma, T. Gao, H. Geng, Q. Han, B. Dai, S. J. Elston, S. M. Morris, C. He, Multiplexed vector beam conversion via complex structured matter. arXiv:2512.22980 (2025).
16. X. Lin, Y. Rivenson, N. T. Yardimci, M. Veli, Y. Luo, M. Jarrahi, A. Ozcan, All-optical machine learning using diffractive deep neural networks. Science 361, 1004-1008 (2018).
17. H. Bezuidenhout, C. Peters, R. Kumar, A. Forbes, I. Nape, Deep diffractive optical neural networks for detecting skyrmionic topologies of light. arXiv:2512.11115 (2025).

We thank the reviewer once again for these thoughtful and constructive comments, which have substantially improved the manuscript, and we hope the revised version now meets with the reviewer's approval.

<u>Response to Reviewers</u>

Manuscript Number: NCOMMS-26-025583

Title: Topologically robust programmable processing arrays using light and matter skyrmions

We sincerely thank both reviewers for their constructive and positive feedback, which we believe has significantly enhanced the quality and clarity of our manuscript.

Our responses are provided below in blue following the comments raised by each reviewer (shown in italics). We provide a **<u>Point-to-Point Reply to All Comments of Reviewers</u>**, which we hope will be beneficial in clarifying the main changes made to the manuscript and addressing overarching points raised during the review. Revisions to the Main article and Supplementary Notes are indicated in **Purple** text in this response below.

Reply to Reviewer #1

In my opinion, the authors have satisfactorily addressed my questions and concerns. I recommend publication of the work.

We sincerely thank the reviewer for the encouraging feedback and for recommending our work for publication. We also appreciate the reviewer's constructive input throughout the review process.

Reply to Reviewer #2

I would like to thank the authors for their detailed responses and for the revisions made to the manuscript. It appears that the authors have addressed the majority of the comments raised by the reviewers in the previous round. The accessibility of the manuscript has been improved, and the novelty of the work is now presented more clearly. In particular, the addition of the topological protection experiment and the per-channel deviation experiment has substantially strengthened the case for the advantages of the proposed scheme. I now believe that the work merits publication in Nature Communications, although several minor and optional revisions may be considered before publication.

We sincerely thank the reviewer for their careful reading of our revised manuscript and for their positive evaluation of our work. We are particularly grateful to the reviewer for noting that the additional experiments have "substantially strengthened the case for the advantages of the proposed scheme", and for the reviewer's view that the work now merits publication in Nature Communications.

We have carefully considered both comments raised by the reviewer and revised the manuscript accordingly. Please allow us to provide a point-by-point response below.

Comment 1: *The authors have revised the colour mapping of the Stokes parameters in Fig. 4 and elsewhere to improve the visibility of the near-uniform boundaries of the skyrmions. However, the saturation/value mapping of the new colour function is not linear with respect to S_3, and the region corresponding to S_3 > 0.9 is almost entirely mapped to white, making it difficult to distinguish between different physical states based on colour. With a standard 8-bit-per-channel colour representation, the values appear to become effectively indistinguishable once S_3 exceeds 0.9. Whilst this may improve the visual appearance of the figures, I believe that it compromises the accuracy of the data representation to some extent. I therefore strongly recommend that the authors readjust the colour function, for instance, so as to provide a resolution of at least 0.01 in S_3 across the relevant range.*

Response 1: We thank the reviewer for this valuable comment. We have re-plotted all figures containing Stokes fields using a color function that is **linear** in S_3, and updated the corresponding color bars.

In the new color function, the hue encodes the azimuthal angle on the Poincaré sphere as before, and the saturation mapping is now linear in S_3. Under a standard 8-bit-per-channel representation, a difference of 0.01 in S_3 corresponds to a change of approximately 2.6 counts (255×0.01), so the resolution of about 0.01 suggested by the reviewer should be satisfied across the relevant range. Meanwhile, $S_3 \approx 1.000$ remains close to pure white, so the visibility of the near-uniform boundary raised in the previous round is also retained.

A comparison of the three color functions across the revisions is provided below for reference.

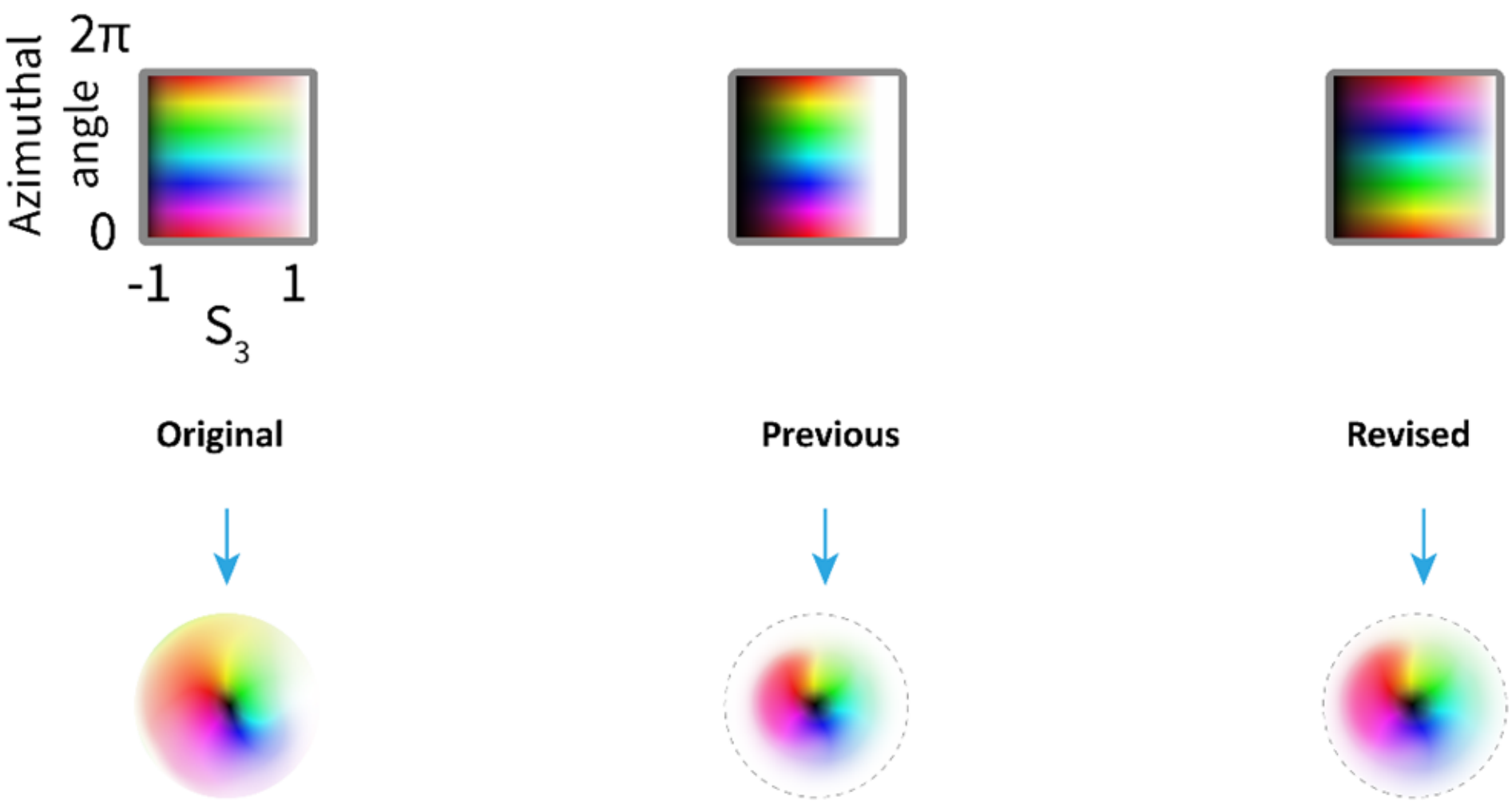

Comparison Figure: The three color functions used across the revisions (top) and an order-1 skyrmion displayed with each (bottom).

The revised Fig. 3 is reproduced below for the reviewer's convenience.

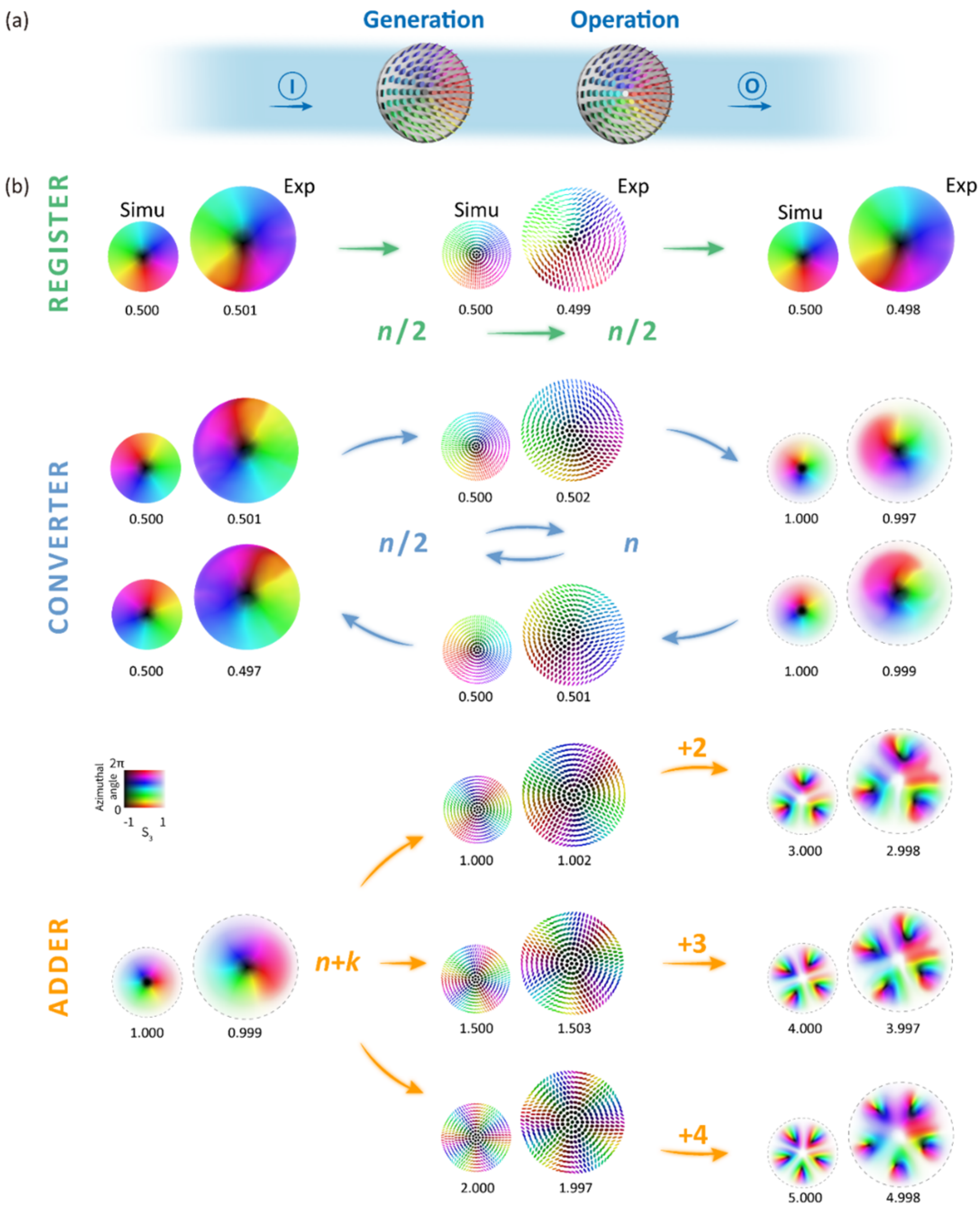

Comment 2: *The authors have also added an additional experiment to demonstrate the topological resilience of optical skyrmions. The result itself is convincing, however, I am curious as to whether this experiment directly demonstrates the robustness of the proposed information-processing scheme. The authors use skyrmions to demonstrate topological resilience, whereas the proposed system utilises merons for information transport. Though the experimentally demonstrated structure appears to be a bimeron configuration, which is topologically equivalent to a skyrmion under a suitable rotation of the order-parameter axis. Thus, the demonstrated topological protection is fundamentally associated with the skyrmion topology rather than with the meron character itself.*

This raises the question of whether topological protection of the merons is actually necessary for the proposed scheme. In the absence of the uniform boundaries characteristic of skyrmions, the domain of an individual meron is not compactifiable in the same manner, and therefore the

direct topological protection arising from the homotopy of the skyrmion texture does not apply straightforwardly. On the other hand, the results for skyrmions without boundary protection already exhibit a certain degree of robustness against perturbations, suggesting that the robustness of the information-carrying states may not necessarily rely on strict topological protection.

I therefore recommend that the authors clarify this point. In particular, it would be useful to discuss whether the robustness demonstrated in the new experiment is expected to carry over to the merons used for information transport, and, more fundamentally, whether topological protection of the merons is required for the reliable operation of the proposed scheme. Clarifying this distinction between topological protection and the more general robustness of the optical states would, in my view, make the argument for the robustness of the proposed system considerably more convincing.

Response 2: We thank the reviewer for this insightful comment. We agree that those points deserve explicit clarification.

We first note that the topological protection observed in our experiments is determined by whether the boundary of the field is preserved (for a non-preserved boundary with protection, please refer to Ref. [1]), rather than by whether the field can be compactified. The former is used to indicate that merons can also have topological protection properties (see Ref. [1] for fractional skyrmion definitions and the theory of protection); the latter is used to define which fields could be regarded as optical skyrmions featuring topological properties (the concept of 'compactification'; see Ref. [1]). Second, we note that even if the field's topological number is relatively stable under the condition that the boundary is corrupted, the topological property may be destroyed.

We then address the reviewer's points below, and have added a paragraph at the end of Methods 2 accordingly.

1. *On whether the demonstrated protection carries over to merons*. Yes, the same topological protection theory applies to merons. As the reviewer notes, the bimeron in Fig. 6a is homotopic to a standard skyrmion, so Fig. 6 directly demonstrates the protection of the skyrmion. A meron, with charge $n/2$, belongs to the fractional skyrmions defined in Ref. [1] (we note that a skyrmion also belongs to the fractional skyrmions, as the compactifiable case), and the topological protection theory of this class has been established in the same work Ref. [1]: a continuous perturbation that is constant along the boundary of the field preserves the skyrmion number, which includes the boundary-preserving disorder in Fig. 6b. Under this condition, the charge of an order-n meron, being $n/2$, therefore remains pinned at $n/2$, just as the charge of an order-n skyrmion remains pinned at n. In our framework, the topological protection therefore holds for merons as long as the equatorial boundary is preserved. The practical cost is that maintaining an equatorial boundary is more complex than maintaining a uniform one, which is why boundary quality is listed as a challenge in the Discussion.

2. *On whether topological protection of the merons is necessary for the scheme*. Strictly speaking, topological protection is not necessary for every successful readout, since, as shown in Fig. 6c, d, the information can still be correctly retrieved within a certain perturbation range

even after the boundary is corrupted. However, topological protection is essential to the topology-enabled robustness of our scheme and is the very reason we chose skyrmions and merons as the information carriers. When the boundary is preserved, the encoded topological charge is guaranteed to remain invariant under continuous perturbations, rather than merely remaining approximately stable within a finite perturbation range. The topological protection of merons is detailed in Point 1 above.

3. *On the distinction between topological protection and general robustness*. Many thanks. We agree that this distinction is worth making explicit. In our experiments (in this paper), topological protection refers to the invariance of the charge when the boundary is preserved: in Fig. 6b, under the boundary-preserving correlated disorder, the measured charge stays at $N_{\mathrm{sk}} \approx 1.000$ up to a peak retardance of $20\ rad$. On the other hand, in our case, general robustness refers to how the charge behaves once the boundary is corrupted (not in a way that can still be protected; see Ref. [1] for details): the topological protection no longer applies, and the charge drifts continuously with perturbation strength (Fig. 6c, d), yet the readout remains correct while the perturbation stays within a certain range. The robustness the reviewer points to in the boundary-corrupting data is of this second kind.

Following the above discussions, we have added the following text at the end of Methods 2:

"In addition, these experiments distinguish topological protection, the invariance of the charge when the boundary is preserved (Fig. 6b), from a more general robustness once the boundary is corrupted, where the topological protection no longer applies and the charge drifts (Fig. 6c, d) but the readout remains correct while the perturbation stays within a certain range.

We also note that the same topological protection theory applies to merons, the transport states in our framework. Both skyrmions and merons belong to the fractional skyrmions defined in Ref. [1], and the topological protection theory of this class has been established in the same work: a continuous perturbation that is constant along the boundary of the field (such as the boundary-preserving disorder in Fig. 6b) preserves the skyrmion number; applied to a meron, whose charge is $n/2$, this pins the charge at $n/2$."

1. A. A. Wang, Z. Zhao, Y. Ma, Y. Cai, R. Zhang, X. Shang, Y. Zhang, J. Qin, Z.-K. Pong, T. Marozsák, B. Chen, H. He, L. Luo, M. J. Booth, S. J. Elston, S. M. Morris, C. He, Topological protection of optical skyrmions through complex media. Light Sci. Appl. 13, 314 (2024).

We are grateful to the reviewer for the careful reading and constructive suggestions throughout both rounds of review, which have substantially improved this work, and we hope the revised manuscript now meets the reviewer's expectations.
